# Lisavanbulin (BAL101553), a novel microtubule inhibitor, plus radiation in patients with newly diagnosed, MGMT promoter unmethylated glioblastoma

**DOI:** 10.1093/noajnl/vdae150

**Published:** 2024-08-28

**Authors:** Matthias Holdhoff, Xiaobu Ye, Roy E Strowd, Burt Nabors, Tobias Walbert, Frank S Lieberman, Stephen J Bagley, John B Fiveash, Joy D Fisher, Serena Desideri, Trisha Surakus, Marc Engelhardt, Thomas Kaindl, Heidi A Lane, Karine Litherland, Stuart A Grossman, Lawrence R Kleinberg

**Affiliations:** The Sidney Kimmel Comprehensive Cancer Center at Johns Hopkins, Baltimore, Maryland, USA; The Sidney Kimmel Comprehensive Cancer Center at Johns Hopkins, Baltimore, Maryland, USA; Wake Forest University School of Medicine, Winston-Salem, North Carolina, USA; University of Alabama, Birmingham, Alabama, USA; Henry Ford Hospital, Detroit, Michigan, USA; University of Pittsburgh, Pittsburgh, Pennsylvania, USA; University of Pennsylvania, Philadelphia, Pennsylvania, USA; University of Alabama, Birmingham, Alabama, USA; The Sidney Kimmel Comprehensive Cancer Center at Johns Hopkins, Baltimore, Maryland, USA; The Sidney Kimmel Comprehensive Cancer Center at Johns Hopkins, Baltimore, Maryland, USA; The Sidney Kimmel Comprehensive Cancer Center at Johns Hopkins, Baltimore, Maryland, USA; Basilea Pharmaceutica International Ltd, Allschwil, Switzerland; Basilea Pharmaceutica International Ltd, Allschwil, Switzerland; Basilea Pharmaceutica International Ltd, Allschwil, Switzerland; Basilea Pharmaceutica International Ltd, Allschwil, Switzerland; The Sidney Kimmel Comprehensive Cancer Center at Johns Hopkins, Baltimore, Maryland, USA; The Sidney Kimmel Comprehensive Cancer Center at Johns Hopkins, Baltimore, Maryland, USA

**Keywords:** glioblastoma, Lisavanbulin, microtubule inhibitor, MGMT promoter unmethylated, radiation

## Abstract

**Background:**

Lisavanbulin (BAL101553) is a small, lipophilic, oral microtubule destabilizer with promising antitumoral activity observed in preclinical glioblastoma (GBM) models.

**Methods:**

This multicenter phase 1 study sought to determine the MTD of oral Lisavanbulin in combination with standard RT (60 Gy/30 fractions) but without temozolomide in patients with newly diagnosed MGMT promoter unmethylated GBM (uGBM). Dose escalation followed a modified 3 + 3 design. Secondary objectives included estimation of OS and PFS and pharmacokinetic analysis.

**Results:**

Twenty-six patients with uGBM (median age, 63 years, 42.3% male, 61.5% with gross total resection, median Karnofsky performance status 80) were enrolled; 2 tumors had an IDH1 mutation. Predefined dose levels of Lisavanbulin, administered daily concomitantly with RT, were: 4 mg (5 pts), 6 mg (5 pts), 8 mg (7 pts), 12 mg (5 pts), and 15 mg (4 pts). The initial starting dose was 8 mg. Due to grade 4 aseptic meningoencephalitis in the first patient, the dose was decreased to 4 mg. Dose escalation resumed and continued to 15 mg with dose-limiting toxicities of grade 2 confusion and memory impairment observed at 12 mg. Avanbulin exposures increased in a relatively dose-proportional manner with increasing oral dose of Lisavanbulin from 4 to 15 mg.

**Conclusions:**

Lisavanbulin in combination with RT was considered safe up to the highest predefined oral dose level of 15 mg daily.

## Lay Summary

Lisavanbulin is a new drug that shows promise for treating glioblastoma, a serious type of brain cancer, in early lab studies. The authors of this study wanted to see if the drug was safe for patients with glioblastoma. To do this, they tested the drug at doses from 4 to 15 mg per day in combination with radiation in 26 patients who had been recently diagnosed with glioblastoma. While some patients experienced side effects, most were not severe. The authors considered the drug to be safe in patients up to a dose of 15 mg per day.

**Key Points**

- Lisavanbulin is an oral microtubule destabilizer with preclinical activity in glioblastoma, including in MGMT promoter unmethylated models.
- Combination of Lisavanbulin with standard radiation in newly diagnosed unmethylated glioblastoma was found safe up to a dose of 15 mg po daily.

**Importance of the Study**

Microtubule-targeted drugs have been of interest in the treatment of glioblastoma (GBM); however, lack of sufficient drug delivery across the blood-brain barrier and toxicities have hindered further development of these agents in primary brain cancers. Lisavanbulin, an oral, small lipophilic microtubule destabilizer showed promising activity in preclinical models of GBM, including in combination with radiation in MGMT promoter unmethylated tumors. This study demonstrated the safety of combining Lisavanbulin with standard radiation in patients with newly diagnosed GBM up to the highest predefined oral dose level of 15 mg per day.

Glioblastoma (GBM) is the most common primary brain cancer in adults. Overall survival has remained poor, and almost all patients die of their disease. Hence, novel treatment options to improve outcomes for these patients are urgently needed. Maximal safe surgical resection followed by treatment with radiation and temozolomide (TMZ), with or without tumor treatment fields is the current yet inadequate standard of care.^1,2^ However, the benefit from TMZ in patients with O^6^-methylguanine-DNA methyltransferase (MGMT) unmethylated GBM (uGBM) is limited,^3–5^ and the omission of TMZ in patients with newly diagnosed uGBM in clinical trials is commonly considered acceptable if novel agents are tested in this patient population *en lieu* of TMZ.^6,7^

Microtubule-targeted drugs play a key role in the treatment of multiple solid tumors and are used as monotherapy, in combination with other systemic drugs, and in combination with RT. However, this class of drugs has not yet been successfully employed in the treatment of primary brain cancers, mainly due to limited drug delivery across the blood-brain barrier as well as concerns of neurotoxicity that were observed in earlier trials with paclitaxel with convection-enhanced delivery.^8–10^

Lisavanbulin (BAL101553), a lysine prodrug of Avanbulin (BAL27862), is a novel, oral microtubule destabilizer that leads to tumor cell death mediated by modulating the spindle assembly checkpoint.^11^ Preclinical data showed promising antitumoral activity in several cancers, including GBM.^12,13^ The drug demonstrated significant activity in orthotopic GBM models including in uGBM. Lisavanbulin is an attractive candidate for drug development in GBM also due to its chemical structure and preclinical evidence suggesting that Avanbulin can cross the blood-brain barrier. Avanbulin is a lipophilic and small molecule (molecular weight: 387 g/mol), and data in rodents showed a 1:1 brain:plasma ratio demonstrating excellent brain penetration.

Microtubule-targeted drugs, such as Lisavanbulin, can be effective in the treatment of multiple solid tumors as monotherapy, in combination with other systemic drugs, and in combination with RT as there appears to be significant synergy.^14–16^ Although preclinical data suggested these drugs could be effective for GBM when administered along with radiotherapy, this class of drugs has not yet been shown to be effective potentially due to limited drug delivery across the blood-brain barrier.^8–10,17,18^ There remains much interest in developing effective strategies to deploy microtubule agents in the therapy of GBM,^19^ and Lisavanbulin is attractive for this purpose as there is preclinical evidence that it is cytotoxic to GBM, a radiosensitizing agent, crosses the blood-brain barrier,^20^ and is suitable for investigation of continuous daily oral administration during radiotherapy to achieve optimal synergy.

In this study, we aimed to identify the maximum tolerated dose of Lisavanbulin in combination with RT in uGBM, without TMZ, during radiation (NCT03250299) as a building block for the development of this drug in newly diagnosed GBM. This is the first human trial of Lisavanbulin in combination with definitive RT in GBM. A prospective clinical trial with Lisavanbulin as a single agent in recurrent GBM was ongoing in Europe by the time our study commenced (NCT02490800), and safety data from that trial guided the selection of the initial dose level of this drug in combination with RT for our study. The MTD in that trial was finally set at 30 mg/day, with no DLTs occurring in any of the 6 patients in this dose cohort.^21^

## Materials and Methods

### Study Design

This was a phase 1, open-label, multicenter study of Lisavanbulin (BAL101553) when administered in combination with RT in patients with newly diagnosed uGBM. The study was sponsored by the Cancer Therapy Evaluation Program (CTEP) of the National Cancer Institute (NCI), and it was conducted by the Adult Brain Tumor Consortium (ABTC). The study was completed prior to and then published after the termination of the ABTC consortium. The primary objective of the study was to determine the highest tolerated dose of Lisavanbulin in combination with standard radiation (30 fractions, 60 Gy) in uGBM using predefined levels of dose escalation. TMZ in combination with RT was omitted to study the effect of Lisavanbulin without the potential additive toxicities from TMZ. The rationale for the omission of TMZ was the overall limited benefit from TMZ in uGBM; the study population was thus patients with newly diagnosed uGBM. Secondary objectives included the estimation of safety and tolerability, determination of overall and progression-free survival, and to assess the pharmacokinetics of Lisavanbulin and Avanbulin (BAL27862). In addition, an exploratory objective was to test tumor tissue for expression of EB1, which was considered a potential biomarker at the time of study initiation.^21^

### Study Eligibility

All patients had histological confirmation of GBM at the time of enrollment (based on WHO Classification of CNS Tumors 2016),^22^ either by biopsy or resection, and tumor tissue needed to be negative for MGMT promoter methylation per the institutions’ standard testing. At the time of final data analysis, diagnoses were adjusted to the WHO Classification of CNS Tumors 2021.^23^ Other eligibility criteria included acceptable organ and marrow function within 14 days prior to starting the study drug, and Karnofsky performance status ≥60%. Patients being treated with steroids had to be on a stable or decreasing dose 5 days before the baseline MRI. Key exclusion criteria were the presence of ataxia or peripheral neuropathy ≥CTCAE grade 2, blood pressure ≥140/90 mmHg, treatment with more than 2 antihypertensive medications, or significant cardiac disease or abnormalities.

### Study Procedures

After eligibility was confirmed, and after patients signed informed consent, patients received standard radiation for 6 weeks (60 Gy in 30 fractions), as well as daily oral Lisavanbulin. The toxicity evaluation period ended after 4 weeks of a rest period after completion of radiation and Lisavanbulin. Patients could continue further treatment as deemed reasonable by the treating physician, for example, by receiving adjuvant temozolomide. Dose-limiting toxicities (DLT) had to be considered at least possibly related to Lisavanbulin or the combination of Lisavanbulin and radiation (see DLT definition in the Protocol, Supplementary Data). Adverse events were recorded and graded according to CTCAEv.4. It is of note that tissue testing for MGMT promoter methylation and IDH mutations was performed as per the individual institutions’ standard testing. Central testing for MGMT promoter methylation or IDH mutations was not performed.

The study was reviewed and approved by all contributing sites’ Institutional Review Boards, and the study was conducted in accordance with the Declaration of Helsinki.

### Pharmacokinetic Analysis

Blood samples were collected to assess the dose proportionality and the single- and multiple-dose PK of Avanbulin after oral administration of Lisavanbulin.

Serial samples were collected on days 1 and 22: pre-dose (0), and 0.5, 1, 2, 4, 6, and 24 hours post-Lisavanbulin administration. The collection window for the samples scheduled for 0.5, 1, 4, and 6 hours post-dose was ±15 minutes. The collection window for the 24-hour sample was ±1 hour.

Plasma concentrations of Lisavanbulin and Avanbulin were determined using validated liquid chromatography with tandem mass spectroscopy (LC-MS/MS). Concentration-time profiles of Lisavabulin and Avanbulin were analyzed by non-compartmental methods using Phoenix WinNonlin (Build 8.3.3.33). Pharmacokinetic parameters and variables were calculated for Lisavanbulin (when/if applicable) and Avanbulin according to standard equations using actual sampling times from the time of oral administration.

Following administration, C_max_ and T_max_ were obtained directly from the experimental observations. If multiple maxima occurred at equal concentrations, the first temporal value was taken.

AUC was calculated using the linear trapezoidal linear interpolation, using actual elapsed time since the start of drug oral administration.

The number of data points included in the regression for determination of λ_z_ and T_½_ after single-dose was determined by visual inspection, but a minimum of 3 data points in the terminal phase, excluding C_max_, were required to estimate λ_z_.

The proportion of AUC_inf_ due to extrapolation (AUC_extr_) was also calculated and expressed as a percentage. If AUC_extr_ was higher than 20%, then the values of AUC_inf_,

CL/F, and Vd/F were considered unreliable and therefore excluded from the summaries.

Dose proportionality analyses were performed on AUC_0-24h_ and C_max_ following PK analysis from data collected on days 1 and on 22 of multiple oral administrations of Lisavanbulin (dose levels of 4 to 15 mg, cohorts 2–6) and were separately examined using the power model.

### Tissue Analysis

Slides were to be prepared from archival FFPE tumor blocks when available and stained for EB1 using a CE-marked immunohistochemistry clinical trial assay. The interpretative criteria for positive EB1-staining were defined as moderate or strong cytoplasmic staining in more than 50% of GBM cells.

### Statistical Considerations

The study was designed as a multicenter, open-label phase I trial to define a maximum tolerated dose with a targeted DLT rate of 33%. The trial safety evaluation period was 10 weeks from initiation of the treatment in combination with 6 weeks of standard radiation therapy. A modified 3 + 3 design was used for the dose-finding with 5 patients per dose cohort to accommodate the long safety evaluation period. The 5 patients per dose cohort would ensure a minimum of 3 evaluable patients who might maintain compliance with the protocol without dropout due to early progression of the disease. The study also was designed to assess the overall safety of the treatment and to describe the pharmacokinetics of Lisavanbulin and Avanbulin in combination with RT. Progression-free survival and overall survival were the secondary objectives of the trial. Progression-free survival and overall survival were measured from the date of trial registration to the date that the event occurred or was censored at the time of database locking. All patients who had received one dose of Lisavanbulin were included in the analysis. Descriptive statistics were used to summarize patient characteristics, toxicity data, and pharmacokinetics. Survival probability was estimated using the Kaplan–Meier method.^24^ The confidence interval of median survival time was constructed by the method of Brookmeyer-Crowley (1982).^25^ All analyses were conducted using the SAS software (version 9.2, SAS Institute).

## Results

### Patient Population

A total of 26 patients with histological diagnosis of uGBM were enrolled in this study between December 2017 and March 2022. The median age of study participants was 63 years (range, 36–76), of which 42.3% were male, and 61.5% had a gross total resection. Baseline demographics are summarized in Table 1. Eighteen of the 26 patients (69%) had died by the time of data analysis. Of note, 2 of the patients had IDH1-mutant tumors that would now be classified as astrocytoma, IDH-mutant, WHO grade 4, not as GBM, based on the WHO 2021 classification of tumors of the CNS.^23^

**Table 1.** Demographics

Patient baseline characteristics	All patients (*N* = 26)
Age: median (range)	63 (36–76)
Race:	
White: no. (%)	23 (88%)
Gender: male no. (%)	11 (42%)
KPS:	
90–100: no. (%)	10 (38%)
70–80: no. (%)	16 (62%)
MGMT unmethylated: no. (%)	26 (100%)
IDH-mutant: no. (%) *	2 (8%)
Measurable disease: no. (%)	19 (73%)
Steroids: Yes: no. (%)	14 (54%)
Anticonvulsant Yes: no. (%)	17 (65%)
Histology:	
Glioblastoma, WHO grade IV: No. (%) *	26 (100%)
Surgery:	
Biopsy: no. (%)	4 (15%)
Subtotal resection: no. (%)	6 (23%)
Gross total resection: no. (%)	16 (62%)
Prior surgery: median (range)	1 (1–2)

### Dose Escalation and Maximum Tolerated Dose

**Figure 1.**
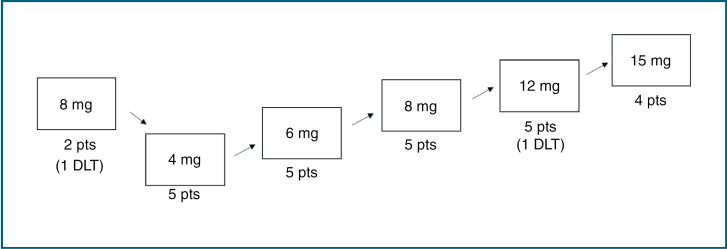
Illustration of dose-finding for Lisavanbulin in combination with standard radiation in newly diagnosed GBM. After the first dose-limiting toxicity at the first dose level (meningoencephalitis), the Lisavanbulin dose was reduced to 4 mg po daily. An additional dose level of 6 mg po daily was added for safety prior to dose escalation back to the initial dose level of 8 mg po daily.

All patients completed their full course of RT as planned. The first patient enrolled in this study was treated with Lisavanbulin 8 mg daily (dose level 1) in combination with RT. During chemoradiation, the patient developed aseptic meningoencephalitis that required hospitalization. Comprehensive clinical work-up, including CSF (cerebrospinal fluid) analysis, did not reveal a clear diagnosis, and the symptoms were therefore attributed to being possibly related to the study drug and considered a DLT, CTCAE (Common Terminology Criteria for Adverse Events) grade 4. Treatment with Lisavanbulin was stopped after 37 days. The patient completed radiation, and the symptoms eventually resolved. Given this event, the study team, with guidance from the Data Safety Monitoring Board, decided to restart enrolling at a lower dose of Lisavanbulin 4 mg daily, with an additional planned dose level of Lisavanbulin 6 mg daily prior to dose escalation back to 8 mg daily. A mandatory safety assessment was introduced and performed 3 weeks after each first patient at each dose level. A second DLT at Lisavanbulin 12 mg was grade 2 confusion and memory impairment; neurocognitive changes in the study were defined as a DLT per protocol, even if the adverse event was graded as CTCAE grade 2. The dose escalation continued to the planned maximum dose of 15 mg daily (Figure 1). The number of patients enrolled at each dose level were Lisavanbulin 4 mg (5 pts), 6 mg (5 pts), 8 mg (7 pts), 12 mg (5 pts), and 15 mg (4 pts). The maximum dose of Lisavanbulin that was tested in combination with RT in newly diagnosed uGBM and that was considered safe was 15 mg daily during radiation.

The planned expansion cohort at the MTD could not be performed due to the termination of funding for the ABTC consortium.

### Safety and Tolerability

Adverse events deemed related to Lisavanbulin or RT, are summarized in Table 2. Grade 3 AEs were hypertension (2), seizure (2), cognitive disturbance (1), cerebral edema (1), hyponatremia (1), and lymphopenia (1). One patient experienced grade 4 aseptic meningoencephalitis (no additional grade 4 AE was observed other than the 1 case of aseptic meningoencephalitis at Lisavanbulin 8 mg po daily). A summary of all related AEs per Lisavanbulin dose level is provided in Supplementary Table 1.

**Table 2.** Toxicity (Adverse Events Attributed to Lisavanbulin and/or Radiation)

Adverse events with all doses combined *N* (% of patients):	Grade 1	Grade 2	Grade 3	Grade 4	Total (*N* = 26)
2 red line to left side of temple	1				1 (4%)
Aseptic meningoencephalitis				1	1 (4%)
Alanine aminotransferase increased	5				5 (19%)
Alopecia	11				11 (42%)
Anemia	3				3 (12%)
Anorexia	4				4 (15%)
Aspartate aminotransferase increased	1				1 (4%)
Balance impairment		1			1 (4%)
Blurred vision	1				1 (4%)
Burn	1				1 (4%)
Carbon dioxide level increased	1				1 (4%)
Splitting of skin on both hands		1			1 (4%)
Cardiac troponin I increased	1				1 (4%)
Cognitive disturbance		1	1		2 (8%)
Confusion	1	1			2 (8%)
Constipation		1			1 (4%)
Creatinine increased	1				1 (4%)
Decreased PO intake	1				1 (4%)
Dehydration		1			1 (4%)
Dermatitis radiation	3	1			4 (15%)
Diarrhea	4				4 (15%)
Dry eye	1				1 (4%)1
Dysarthria		1			1 (4%)
Dysgeusia	1				1 (4%)
Dysphasia		1			1 (4%)
Epilation	2				2 (8%)
Edema cerebral	1		1		2 (8%)
Edema face	1				1 (4%)
Facial rash	1				1 (4%)
Facial muscle weakness		1			1 (4%)
Facial nerve disorder		1			1 (4%)
Fatigue	8	9			17 (65%)
Fever		1			1 (4%)
Heart burn	1				1 (4%)
Headache		2			2 (8%)
Hypercalcemia	3				3 (12%)
Hyperkalemia	1				1 (4%)
Hypermagnesemia	1				1 (4%)
Hyperphosphatemia	2				2 (8%)
Hypertension			2		2 (8%)
Hyponatremia	2	2	1		5 (19%)
Hypophosphatemia	2				2 (8%)
Low chloride level	1				1 (4%)
Lethargy		2			2 (8%)
Lymphocyte count decreased		1	1		2 (8%)
Memory impairment	2	1			3 (12%)
Muscle cramp		1			1 (4%)
Muscle weakness left-sided	1				1 (4%)
Muscle weakness lower limb	1				1 (4%)
Muscle weakness upper limb		1			1 (4%)
Myocarditis		1			1 (4%)
Nausea	6				6 (23%)
Oral dysesthesia	1				1 (4%)
Pain of skin	1				1 (4%)
Peripheral sensory neuropathy		1			1 (4%)
Platelet count decreased	2	1			3 (12%)
Right side lip numbness	1				1 (4%)
Stomach cramps	1				1 (4%)1
Seizure	1		2		3 (12%)
Sinus tachycardia	1				1 (4%)
Urinary incontinence		1			1 (4%)
Vasogenic edema		1			1 (4%)
Vomiting	2				2 (8%)
White blood cell decreased	2				2 (8%)

### Pharmacokinetics

There were 25 patients with evaluable Avanbulin PK profiles for day 1 and 24 patients with evaluable Avanbulin PK profiles for Day 22. There were no evaluable Lisavanbulin PK profiles for Day 1 and Day 22 as Lisavanbulin concentrations were below the lower limit of quantitation.

**Figure 2.**
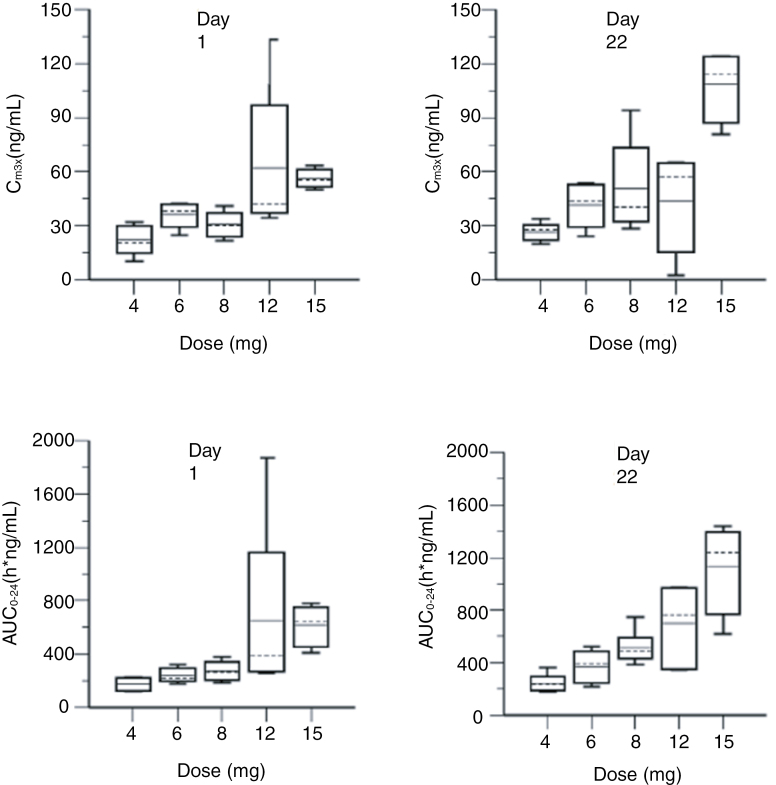
Pharmacokinetics. Box plots of Avanbulin pharmacokinetic parameters (C_max_ and AUC_0–24 h_) versus dose by PK day (days 1 and 22; Linear scale).

Figure 2 presents the box plots for Avanbulin pharmacokinetic parameters (C_max_ and AUC_0-24h_) versus dose by PK day (days 1 and 22). Supplementary Table 2 presents the summary statistics of Avanbulin PK parameters. Supplementary Table 3 presents the intra-patient accumulation ratios for C_max_ and AUC_0-24h_ by dose group. Following oral administration of doses of 4 to 15 mg Lisavanbulin on days 1 and 22, Avanbulin geometric mean C_max_ ranged from 20.6 to 55.9 ng/mL and from 26.2 to 107 ng/mL, and geometric mean AUC_0–24h_ ranged from 170 to 600 h*ng/mL and from 234 to 1080 h*ng/mL, respectively. When comparing exposures on day 22 to those observed on day 1, the data show no significant drug accumulation over time. Avanbulin apparent T_1/2_ was estimated to range from 9.53 to 9.65 hours following oral administration of Lisavanbulin. The geometric mean CL/F for Avanbulin was estimated to range from 18.4 to 22.8 L/h.

Based on the results from the power model and visual inspection, Avanbulin exposure (based on C_max_ and AUC_0–24 h_ on days 1 and 22) increased in a relatively dose-proportional manner with increasing oral dose of Lisavanbulin from 4 to 15 mg (Supplementary Table 4).

### Immunohistochemistry for EB1

EB1 was tested as an exploratory biomarker for response to treatment with Lisavanbulin. FFPE (Formalin Fixed Paraffin Embedded) tissue of 13 of the 26 patients in this study were analyzed, and all stainings were negative. EB1 was not found to be a valid biomarker of response in the European trial with Lisavanbulin in recurrent GBM.^26^ Therefore, IHC for EB1 expression of the 13 remaining tissues was omitted.

### Survival Data

Assessment of overall and progression-free survival was a secondary objective of this study. Data are presented in the Supplementary Data (Supplementary Figure 1).

## Discussion

In this study, we demonstrated the safety and tolerability of this drug when combined with RT in patients with newly diagnosed GBM. We enrolled patients with uGBM to initially study the safety of combination with RT alone.

Microtubule-targeted drugs have been of significant interest in brain cancer research, and preclinical data on the efficacy of these drugs exist; however, drug delivery and toxicity have previously been roadblocks for further development of this class of anticancer agents. Lisavanbulin is an interesting compound for combination with standard therapy in newly diagnosed GBM for several reasons. Preclinical data showed excellent activity in both MGMT methylated and unmethylated GBM models, and the drug has molecular features and preclinical data supporting appropriate drug delivery across the blood-brain barrier.^11–13^

In this study, we obtained comprehensive pharmacokinetic data of Lisavanbulin at dose levels of 4, 6, 8, 12, and 15 mg daily during RT. Avanbulin exposure (based on C_max_ and AUC_0-24h_ on days 1 and 22) increased in a relatively dose-proportional manner with increasing oral dose of Lisavanbulin from the 4 to 15 mg dose level. No significant drug accumulation was observed.

Limitations of this study included the lack of a dose expansion cohort at the highest tolerated dose level, which had been planned, but which could not be pursued due to the expiration of NCI funding of the ABTC. In this context, it is worth noting that in patients with recurrent GBM, the MTD of Lisavanbulin given as monotherapy was set at 30 mg/day.^21^ Also, MGMT promoter methylation testing was not performed centrally due to concerns about the delay of the start of radiation in these patients with newly diagnosed GBM. Central review is commonly preferred to assure homogeneity among the patient population as low-level MGMT promoter methylation, not captured by all MGMT promoter tests, may confer some benefit from TMZ for patients with uGBM; however, this would not have affected the primary endpoint of our study, which was safety and tolerability. In addition, at the start of this study, the diagnosis of GBM was based on the 2016 version of the WHO classification. Two of the patients enrolled had tumors that harbored an IDH1 mutation and which would currently not be classified as GBM but as astrocytoma, IDH-mutant, WHO grade 4 based on the WHO 2021 classification of tumors of the CNS.^23^ This, however, did not affect the primary objective of this study which was safety and tolerability, and this study demonstrated the safety of combining Lisavanbulin and RT in newly diagnosed uGBM, up to the predefined dose level of Lisavanbulin 15 mg daily. In addition, treatment after the end of the study evaluation period, i.e. after 4 weeks post radiation and Lisavanbulin treatment was not systemically recorded; further treatment was at the discretion of the treating physician.

OS and PFS observed in this study, which was secondary, not primary objectives (Supplementary Data) were similar to and not better than that reported in the literature for uGBM.^3^

It is of note that Lisavanbulin has not yet been studied in combination with TMZ, including in MGMT promoter methylated GBM. If further drug development of this drug were considered in GBM in the future, the addition of Lisavanbulin to RT and TMZ as well as to standard adjuvant TMZ (150-200 mg/m^2^ for 5 days every 28 days) may be considered. This, however, would require an initial safety assessment with dose escalations for Lisavanbulin both during chemoradiation and in combination with adjuvant TMZ.

## Supplementary Material

vdae150_suppl_Supplementary_Materials

## Data Availability

The participants of this study did not give written consent for their data to be shared publicly, so due to the sensitive nature of the research supporting data cannot be shared.
