# Supplementary material for: Lisavanbulin (BAL101553), a novel microtubule inhibitor, plus radiation in patients with newly diagnosed, MGMT promoter unmethylated glioblastoma
Source: Neurooncol Adv. 2024 Aug 28;6(1):vdae150. doi: 10.1093/noajnl/vdae150 (PMC11450402; doi:10.1093/noajnl/vdae150)
Supplement: vdae150_suppl_Supplementary_Materials [file vdae150_suppl_Supplementary_Materials.zip › Supplementary Files R1.docx]

*Holdhoff M et al. Lisavanbulin (BAL101553), a novel microtubule inhibitor plus radiation in patients with newly diagnosed, MGMT promoter unmethylated glioblastoma*

**Supplementary Data Files**

**Supplementary Table 1: All AEs Related to the Study Drugs by dose levels.**

| **Dose level: 4mg**  **Adverse Events** N (% of patients)**:** | **Grade 1** | **Grade 2** | **Grade 3** | **Grade 4** | **Total**  **(n=5)** |
| --- | --- | --- | --- | --- | --- |
| Alanine aminotransferase increased | 1 |  |  |  | 1 (20%) |
| Alopecia | 3 |  |  |  | 3 (60%) |
| Anemia | 1 |  |  |  | 1 (20%) |
| Anorexia | 1 |  |  |  | 1 (20%) |
| Balance impairment |  | 1 |  |  | 1 (20%) |
| Burn | 1 |  |  |  | 1 (20%) |
| Splitting of skin on both hands |  | 1 |  |  | 1 (20%) |
| Creatinine increased | 1 |  |  |  | 1 (20%) |
| Decreased PO intake | 1 |  |  |  | 1 (20%) |
| Diarrhea | 2 |  |  |  | 2 (40%) |
| Dysarthria |  | 1 |  |  | 1 (20%) |
| Dysgeusia | 1 |  |  |  | 1 (20%) |
| Dysphasia |  | 1 |  |  | 1 (20%) |
| Edema cerebral |  |  | 1 |  | 1 (20%) |
| Facial muscle weakness |  | 1 |  |  | 1 (20%) |
|  |  |  |  |  |  |
| Facial nerve disorder |  | 1 |  |  | 1 (20%) |
| Fatigue | 1 | 3 |  |  | 4 (80%) |
| Heart burn | 1 |  |  |  | 1 (20%) |
| Hyperkalemia | 1 |  |  |  | 1 (20%) |
| Hyperphosphatemia | 1 |  |  |  | 1 (20%) |
| Lymphocyte count decreased |  | 1 |  |  | 1 (20%) |
| Memory impairment | 2 |  |  |  | 2 (40%) |
| Muscle cramp |  | 1 |  |  | 1 (20%) |
| Muscle weakness left-sided | 1 |  |  |  | 1 (20%) |
| Muscle weakness lower limb | 1 |  |  |  | 1 (20%) |
| Muscle weakness upper limb |  | 1 |  |  | 1 (20%) |
| Nausea | 3 |  |  |  | 3 (60%) |
| Pain of skin | 1 |  |  |  | 1 (20%) |
| Peripheral sensory neuropathy |  | 1 |  |  | 1 (20%) |
| Right side lip numbness | 1 |  |  |  | 1 (20%) |
| Stomach Cramps | 1 |  |  |  | 1 (20%) |
| Seizure | 1 |  |  |  | 1 (20%) |
| Vomiting | 2 |  |  |  | 2 (40%) |

| **Dose level: 6mg**  **Adverse Events** N (% of patients)**:** | **Grade 1** | **Grade 2** | **Grade 3** | **Grade 4** | **Total**  **(n=5)** |
| --- | --- | --- | --- | --- | --- |
| Alanine aminotransferase increased | 1 |  |  |  | 1 (20%) |
| Alopecia | 3 |  |  |  | 3 (60%) |
| Anorexia | 1 |  |  |  | 1 (20%) |
| Carbon dioxide level increased | 1 |  |  |  | 1 (20%) |
| Cognitive disturbance |  |  | 1 |  | 1 (20%) |
| Confusion | 1 |  |  |  | 1 (20%) |
| Dehydration |  | 1 |  |  | 1 (20%) |
| Dermatitis radiation | 1 | 1 |  |  | 2 (40%) |
| Diarrhea | 1 |  |  |  | 1 (20%) |
| Facial rash | 1 |  |  |  | 1 (20%) |
| Fatigue | 3 | 1 |  |  | 4 (80%) |
| Headache |  | 1 |  |  | 1 (20%) |
| Hypermagnesemia | 1 |  |  |  | 1 (20%) |
| Hyponatremia |  | 1 |  |  | 1 (20%) |
| Low chloride level | 1 |  |  |  | 1 (20%) |
| Lethargy |  | 2 |  |  | 2 (40%) |
| Nausea | 1 |  |  |  | 1 (20%) |
| Platelet count decreased | 1 | 1 |  |  | 2 (40%) |
| Seizure |  |  | 1 |  | 1 (20%) |
| Vasogenic edema |  | 1 |  |  | 1 (20%) |

| **Dose level: 8mg**  **Adverse Events** N (% of patients)**:** | **Grade 1** | **Grade 2** | **Grade 3** | **Grade 4** | **Total**  **(n=7)** |
| --- | --- | --- | --- | --- | --- |
| 2 red lines to 2 left side of temple | 1 |  |  |  | 1 (14%) |
| Aseptic meningoencephalitis |  |  |  | 1 | 1 (14%) |
| Alanine aminotransferase increased | 1 |  |  |  | 1 (14%) |
| Alopecia | 3 |  |  |  | 3 (43%) |
| Anemia | 2 |  |  |  | 2 (29%) |
| Anorexia | 1 |  |  |  | 1 (14%) |
| Blurred vision | 1 |  |  |  | 1 (14%) |
| Constipation |  | 1 |  |  | 1 (14%) |
| Dermatitis radiation | 1 |  |  |  | 1 (14%) |
| Dry eye | 1 |  |  |  | 1 (14%) |
| Edema cerebral | 1 |  |  |  | 1 (14%)1 |
| Edema face | 1 |  |  |  | 1 (14%) |
| Fatigue | 3 | 1 |  |  | 4 (57%) |
| Fever |  | 1 |  |  | 1 (14%) |
| Headache |  | 1 |  |  | 1 (14%) |
| Hyperphosphatemia | 1 |  |  |  | 1 (14%) |
| Hypertension |  |  | 1 |  | 1 (14%) |
| Hyponatremia | 1 |  | 1 |  | 2 (29%) |
| Hypophosphatemia | 2 |  |  |  | 2 (29%) |
| Lymphocyte count decreased |  |  | 1 |  | 1 (14%) |
| Nausea | 1 |  |  |  | 1 (14%) |
| Oral dysesthesia | 1 |  |  |  | 1 (14%) |
| Seizure |  |  | 1 |  | 1 (14%) |
| Sinus tachycardia | 1 |  |  |  | 1 (14%) |
| White blood cell decreased | 1 |  |  |  | 1 (14%) |

| **Dose level: 12mg**  **Adverse Events** N (% of patients)**:** | **Grade 1** | **Grade 2** | **Grade 3** | **Grade 4** | **Total**  **(n=5)** |
| --- | --- | --- | --- | --- | --- |
| Alanine aminotransferase increased | 1 |  |  |  | 1 (20%) |
| Alopecia | 1 |  |  |  | 1 (20%) |
| Anorexia | 1 |  |  |  | 1 (20%) |
| Cognitive disturbance |  | 1 |  |  | 1 (20%) |
| Confusion |  | 1 |  |  | 1 (20%) |
| Dermatitis radiation | 1 |  |  |  | 1 (20%) |
| Diarrhea | 1 |  |  |  | 1 (20%) |
| Fatigue |  |  | 3 |  | 3 (60%) |
| Hypercalcemia | 2 |  |  |  | 2 (40%) |
| Hypertension |  |  | 1 |  | 1 (20%)1 |
| Hyponatremia | 1 | 1 |  |  | 2 (40%) |
| Memory impairment |  | 1 |  |  | 1 (20%) |
| Nausea | 1 |  |  |  | 1 (20%) |
| Urinary incontinence |  | 1 |  |  | 1 (20%) |

| **Dose level: 15mg**  **Adverse Events** N (% of patients)**:** | **Grade 1** | **Grade 2** | **Grade 3** | **Grade 4** | **Total**  **(n=4)** |
| --- | --- | --- | --- | --- | --- |
| Alanine aminotransferase increased | 1 |  |  |  | 1 |
| Alopecia | 1 |  |  |  | 1 |
| Aspartate aminotransferase increased | 1 |  |  |  | 1 |
| Cardiac troponin I increased | 1 |  |  |  | 1 |
| EPILATION | 2 |  |  |  | 2 |
| Fatigue | 1 | 1 |  |  | 2 |
| Myocarditis |  | 1 |  |  | 1 |
| Platelet count decreased | 1 |  |  |  | 1 |
| White blood cell decreased | 1 |  |  |  | 1 |

**Supplementary Table 2.** Summary statistics of plasma Avanbulin pharmacokinetic parameters by dose group

| **Parameter** | **Statistic** | **Cohort 2**  **4 mg** | | **Cohort 3**  **6 mg** | | **Cohort 4**  **8 mg** | | **Cohort 5**  **12 mg** | | **Cohort 6**  **15 mg** | |
| --- | --- | --- | --- | --- | --- | --- | --- | --- | --- | --- | --- |
|  |  | Day 1 | Day 22 | Day 1 | Day 22 | Day 1 | Day 22 | Day 1 | Day 22 | Day 1 | Day 22 |
| C_max_  (ng/mL) | N  Mean  SD  Min  Median  Max  GeoMean  CV% GM | 5  22.1  8.46  10.6  20.4  32.3  20.6  45.4 | 5  26.7  5.34  19.9  27.8  34.0  26.2  20.5 | 5  35.9  7.17  25.1  37.9  42.3  35.3  21.9 | 5  41.6  12.5  24.4  43.7  53.9  39.9  34.0 | 6  30.6  7.88  21.6  30.2  41.2  29.7  26.5 | 6  50.6  25.0  28.2  40.6  93.7  46.3  47.5 | 5  62.0  41.0  34.6  42.1  133  54.1  58.9 | 4  53.7  17.8  27.5  60.9  65.3  50.8  43.1 | 4  56.1  5.45  49.9  55.7  63.1  55.9  9.69 | 4  108  20.3  80.7  114  124  107  20.3 |
| C_max_/Dose  ((ng/mL)/(mg)) | N  Mean  SD  Min  Median  Max  GeoMean  CV% GM | 5  5.53  2.11  2.65  5.10  8.08  5.16  45.4 | 5  6.67  1.33  4.98  6.95  8.50  6.56  20.5 | 5  5.99  1.19  4.18  6.32  7.05  5.88  21.9 | 5  6.93  2.08  4.07  7.28  8.98  6.65  34.0 | 6  3.82  0.984  2.70  3.77  5.15  3.72  26.5 | 6  6.33  3.12  3.53  5.07  11.7  5.78  47.5 | 5  5.16  3.41  2.88  3.51  11.1  4.51  58.9 | 4  4.47  1.48  2.29  5.08  5.44  4.23  43.1 | 4  3.74  0.363  3.33  3.71  4.21  3.73  9.69 | 4  7.21  1.35  5.38  7.60  8.27  7.11  20.3 |
| T_max_ (h) | N  Mean  SD  Min  Median  Max  GeoMean  CV% GM | 5  2.27  1.10  1.00  1.98  4.00  2.06  53.4 | 5  1.32  0.427  0.833  1.33  1.92  1.26  33.8 | 5  1.30  0.502  0.900  1.00  2.08  1.24  36.9 | 5  1.43  0.592  0.850  1.18  2.13  1.34  43.2 | 6  1.85  1.37  0.500  1.50  3.95  1.43  96.7 | 6  1.49  0.654  0.500  1.64  2.17  1.34  60.9 | 5  1.64  0.719  0.800  2.00  2.43  1.49  53.4 | 4  2.15  1.55  0.750  1.93  4.00  1.71  95.9 | 4  3.08  1.11  1.75  3.29  4.00  2.92  41.4 | 4  1.61  0.402  1.05  1.69  2.00  1.57  28.5 |
| AUC_0-last_  (h*ng/mL) | N  Mean  SD  Min  Median  Max  GeoMean  CV% GM | 5  141  61.9  63.9  121  225  129  50.9 | 5  240  72.4  173  234  359  233  28.6 | 5  239  53.2  179  220  312  234  22.3 | 5  350  157  126  394  506  314  62.1 | 6  276  71.6  185  271  375  269  27.0 | 6  521  125  381  506  745  510  23.0 | 5  653  707  254  379  1910  469  97.6 | 4  635  282  347  614  966  587  49.3 | 4  631  170  411  668  776  612  30.1 | 4  1160  370  617  1280  1450  1100  40.6 |
| T_last_ (h) | N  Mean  SD  Min  Median  Max  GeoMean  CV% GM | 5  20.1  8.03  5.77  23.7  23.9  17.9  70.2 | 5  23.6  0.480  22.8  23.8  24.0  23.6  2.05 | 5  23.7  0.714  22.5  23.9  24.2  23.7  3.07 | 5  20.0  7.98  5.78  23.3  24.2  17.8  69.7 | 6  24.6  1.11  23.7  24.1  26.1  24.6  4.44 | 6  24.6  1.72  23.7  24.0  28.1  24.5  6.65 | 5  23.6  0.741  23.0  23.5  24.8  23.6  3.10 | 4  23.7  0.168  23.5  23.8  23.9  23.7  0.711 | 4  25.2  2.00  24.0  24.3  28.2  25.1  7.68 | 4  24.8  2.76  22.4  24.1  28.8  24.7  10.8 |
| AUC_0-24_ or AUC_0-T_  (h*ng/mL) | N  Mean  SD  Min  Median  Max  GeoMean  CV% GM | 3  175  52.1  121  178  226  170  32.0 | 5  241  71.7  176  235  359  234  28.1 | 5  241  56.4  179  220  322  236  23.3 | 5  372  128  221  392  521  353  38.2 | 6  274  72.3  186  267  375  266  27.2 | 6  516  127  383  488  744  504  23.4 | 5  648*  687  256  385  1870  471  95.6 | 3  696  318  348  767  972  638  57.8 | 4  617  160  411  640  776  600  28.6 | 4  1130  358  619  1240  1440  1080  39.4 |
| AUC_0-24_/Dose  (h*ng/mL/mg) | N  Mean  SD  Min  Median  Max  GeoMean  CV% GM | 3  43.8  13.0  30.4  44.6  56.4  42.4  32.0 | 5  60.4  17.9  44.0  58.7  89.9  58.5  28.1 | 5  40.1  9.40  29.9  36.7  53.6  39.3  23.3 | 5  62.0  21.4  36.8  65.3  86.8  58.9  38.2 | 6  34.2  9.04  23.2  33.3  46.9  33.2  27.2 | 6  64.5  15.9  47.9  61.0  93.0  63.0  23.4 | 5  54.0  57.3  21.3  32.1  156  39.3  95.6 | 3  58.0  26.5  29.0  63.9  81.0  53.2  57.8 | 4  41.1  10.6  27.4  42.7  51.7  40.0  28.6 | 4  75.6  23.9  41.3  82.5  96.0  72.0  39.4 |
| AUC_0-∞_  (h*ng/mL) | N  Mean  SD  Min  Median  Max  GeoMean  CV% GM | 1  197  NA  197  197  197  197  NA | 3  247  41.1  213  235  293  245  16.4 | 1  203  NA  203  203  203  203  NA | 1  316  NA  316  316  316  316  NA | 1  341  NA  341  341  341  341  NA | NA  NA  NA  NA  NA  NA  NA  NA | 2  308  31.6  286  308  331  308  10.3 | NA  NA  NA  NA  NA  NA  NA  NA | 1  919  NA  919  919  919  919  NA | NA  NA  NA  NA  NA  NA  NA  NA |
| AUC_0-∞_/Dose  (h*ng/mL/mg) | N  Mean  SD  Min  Median  Max  GeoMean  CV% GM | 1  49.4  NA  49.4  49.4  49.4  49.4  NA | 3  61.7  10.3  53.3  58.7  73.2  61.2  16.4 | 1  33.9  NA  33.9  33.9  33.9  33.9  NA | 1  52.7  NA  52.7  52.7  52.7  52.7  NA | 1  42.6  NA  42.6  42.6  42.6  42.6  NA | NA  NA  NA  NA  NA  NA  NA  NA | 2  25.7  2.63  23.8  25.7  27.6  25.6  10.3 | NA  NA  NA  NA  NA  NA  NA  NA | 1  61.3  NA  61.3  61.3  61.3  61.3  NA | NA  NA  NA  NA  NA  NA  NA  NA |
| Apparent T_1/2_ (h) | N  Mean  SD  Min  Median  Max  GeoMean  CV% GM | 1  7.63  NA  7.63  7.63  7.63  7.63  NA | 2  9.66  0.277  9.46  9.66  9.85  9.65  2.87 | 1  8.12  NA  8.12  8.12  8.12  8.12  NA | 1  9.53  NA  9.53  9.53  9.53  9.53  NA | 1  10.9  NA  10.9  10.9  10.9  10.9  NA | NA  NA  NA  NA  NA  NA  NA  NA | 2  9.00  1.09  8.23  9.00  9.77  8.97  12.1 | NA  NA  NA  NA  NA  NA  NA  NA | 1  11.5  NA  11.5  11.5  11.5  11.5  NA | NA  NA  NA  NA  NA  NA  NA  NA |
| CL/F (L/h) | N  Mean  SD  Min  Median  Max  GeoMean  CV% GM | 1  22.4  NA  22.4  22.4  22.4  22.4  NA | 2  18.6  2.99  16.4  18.6  20.7  18.4  16.3 | 1  33.5  NA  33.5  33.5  33.5  33.5  NA | 1  22.8  NA  22.8  22.8  22.8  22.8  NA | 1  29.6  NA  29.6  29.6  29.6  29.6  NA | NA  NA  NA  NA  NA  NA  NA  NA | 2  45.1  2.58  43.3  45.1  46.9  45.1  5.73 | NA  NA  NA  NA  NA  NA  NA  NA | 1  21.4  NA  21.4  21.4  21.4  21.4  NA | NA  NA  NA  NA  NA  NA  NA  NA |
| V_d_/F (L) | N  Mean  SD  Min  Median  Max  GeoMean  CV% GM | 1  247  NA  247  247  247  247  NA | 2  259  49.0  224  259  294  257  19.2 | 1  392  NA  392  392  392  392  NA | 1  314  NA  314  314  314  314  NA | 1  466  NA  466  466  466  466  NA | NA  NA  NA  NA  NA  NA  NA  NA | 2  584  37.2  557  584  610  583  6.39 | NA  NA  NA  NA  NA  NA  NA  NA | 1  353  NA  353  353  353  353  NA | NA  NA  NA  NA  NA  NA  NA  NA |

**Patient CDI-CS-004-WF-1601-0299 had higher AUC_0-24_ exposure on Day 1 compared to the other patients in cohort 5 (12 mg). After excluding this patient, mean C_max_ would be 44.2 ng/mL and mean AUC_0-24_ would be 343 h*ng/mL.*

**Supplementary Table 3. Mean C_max_ and AUC_0-24h_ accumulation ratios (Racc) by dose group**

| **Dose**  **(mg)** | **Subject ID** | **Day 1**  **C_max_**  **(ng/mL)** | **Day 22**  **C_max_**  **(ng/mL)** | **Racc**  **C_max_** | **Mean** | **SD** | **Day 1**  **AUC_0-24_ (h*ng/mL)** | **Day 22**  **AUC_0-24_ (h*ng/mL)** | **Racc**  **AUC_0-24_** | **Mean** | **SD** |
| --- | --- | --- | --- | --- | --- | --- | --- | --- | --- | --- | --- |
| 4 | CDI-CS-004-HF-1601-0563 | 20.4 | 28.2 | 1.38 | 1.40 | 0.727 |  | 243 | NC | 1.38 | 0.262 |
|  | CDI-CS-004-HF-1601-0564 | 19.0 | 19.9 | 1.05 |  |  | 121 | 176 | 1.45 |  |  |
|  | CDI-CS-004-HF-1601-0565 | 28.2 | 34.0 | 1.21 |  |  | 226 | 359 | 1.59 |  |  |
|  | CDI-CS-004-JH-1601-0757 | 10.6 | 27.8 | 2.62 |  |  |  | 235 | NC |  |  |
|  | CDI-CS-004-UA-1601-0227 | 32.3 | 23.4 | 0.724 |  |  | 178 | 194 | 1.09 |  |  |
| 6 | CDI-CS-004-JH-1601-0765 | 25.1 | 24.4 | 0.972 | 1.14 | 0.199 | 179 | 263 | 1.47 | 1.52 | 0.290 |
|  | CDI-CS-004-PT-1601-0061 | 32.7 | 43.7 | 1.34 |  |  | 211 | 221 | 1.05 |  |  |
|  | CDI-CS-004-UA-1601-0230 | 42.3 | 53.9 | 1.27 |  |  | 220 | 392 | 1.78 |  |  |
|  | CDI-CS-004-UP-1601-0233 | 37.9 | 33.8 | 0.892 |  |  | 273 | 465 | 1.71 |  |  |
|  | CDI-CS-004-WF-1601-0260 | 41.7 | 52.0 | 1.25 |  |  | 322 | 521 | 1.62 |  |  |
| 8 | CDI-CS-004-JH-1601-0773 |  | 93.7 | NC | 1.30 | 0.327 |  | 744 | NC | 1.65 | 0.261 |
|  | CDI-CS-004-PT-1601-0066 | 41.2 | 67.1 | 1.63 |  |  | 375 | 549 | 1.46 |  |  |
|  | CDI-CS-004-UA-1601-0236 | 24.2 | 40.2 | 1.66 |  |  | 337 | 526 | 1.58 |  |  |
|  | CDI-CS-004-UA-1601-0237 | 25.4 | 28.2 | 1.11 |  |  | 211 | 441 | 2.09 |  |  |
|  | CDI-CS-004-WF-1601-0252 | 34.9 | 40.9 | 1.17 |  |  | 263 | 383 | 1.46 |  |  |
|  | CDI-CS-004-WF-1601-0255 | 21.6 |  | NC |  |  | 186 |  | NC |  |  |
|  | CDI-CS-004-WF-1601-0279 | 36.1 | 33.6 | 0.931 |  |  | 271 | 450 | 1.66 |  |  |
| 12 | CDI-CS-004-JH-1601-0776 | 42.1 | 65.3 | 1.55 | 1.23 | 0.428 | 454 | 972 | 2.14 | 1.83 | 0.413 |
|  | CDI-CS-004-JH-1601-0781 | 61.0 | 57.3 | 0.939 |  |  | 256 | 348 | 1.36 |  |  |
|  | CDI-CS-004-UA-1601-0240 | 39.2 | 64.5 | 1.65 |  |  | 385 | 767 | 1.99 |  |  |
|  | CDI-CS-004-WF-1601-0298 | 34.6 | 27.5 | 0.795 |  |  | 277 |  | NC |  |  |
|  | CDI-CS-004-WF-1601-0299 | 133 |  | NC |  |  | 1870 |  | NC |  |  |
| 15 | CDI-CS-004-UA-1601-0243 | 54.9 | 80.7 | 1.47 | 1.94 | 0.410 | 411 | 619 | 1.51 | 1.82 | 0.298 |
|  | CDI-CS-004-UA-1601-0244 | 63.1 | 124 | 1.97 |  |  | 776 | 1440 | 1.86 |  |  |
|  | CDI-CS-004-WF-1601-0306 | 49.9 | 123 | 2.47 |  |  | 579 | 1280 | 2.21 |  |  |
|  | CDI-CS-004-WF-1601-0313 | 56.5 | 105 | 1.86 |  |  | 701 | 1190 | 1.70 |  |  |

NC: Not Calculated

**Supplementary Table 4.** Dose proportionality analysis (power model) of plasma Avanbulin following Lisavanbulin dose administration on Day 1 and Day 22

| **Day** | **PK parameter** | **Slope (β)** | **Standard Error** | **95% confidence interval for slope** |
| --- | --- | --- | --- | --- |
| Day 1 | C_max_ | 0.703 | 0.181 | -0.0738, 1.48 |
|  | AUC_0-24_ | 1.15 | 0.124 | 0.615, 1.68 |
| Day 22 | C_max_ | 1.04 | 0.310 | -0.296, 2.37 |
|  | AUC_0-24_ | 1.08 | 0.090 | 0.675, 1.48 |

Dose proportionality was not rejected if the 95% confidence interval for the slope included the value of 1.

**Supplementary Figure 1:**


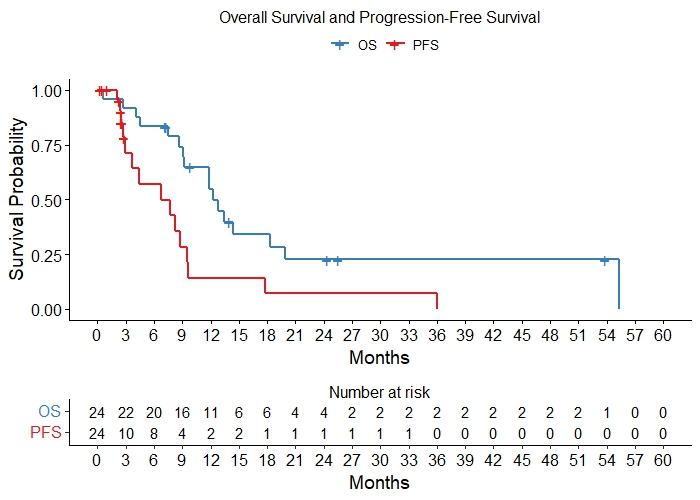


**Figure.** Median overall and progression-free survival in MGMT promoter unmethylated, newly diagnosed patients with GBM, IDH-mutant (N=24) treated with Lisavanbulin and radiation.

Assessment of overall and progression free survival was a secondary objective of this study. The estimated median OS of the 24 patients with uGBM per 2021 WHO classification (i.e., IDH-wildtype) in this trial was 12.1 months (95%CI: 9.0-18.2 months; Supplementary Figure 1, above), and the estimated median PFS was 6.5 months (95%CI: 2.6-9.4 months). The estimated OS including all 26 patients, per protocol, was 12.6 months (95%CI: 9.0-18.2 months) and median PFS 6.5 months (95%CI: 3.6-9.4 months). Of the 2 patients whose tumors harbored an IDH-1 mutation and were therefore reclassified astrocytoma, IDH-mutant, WHO grade 4, one patient was treated with Lisavanbulin 6 mg daily, progressed 18 months after initial surgery, and died after 19 months; the other patient received Lisavanbulin 15 mg daily and had not progressed after 10 months, the time of data close out.
