# Supplementary figures and images for: Lisavanbulin (BAL101553), a novel microtubule inhibitor, plus radiation in patients with newly diagnosed, MGMT promoter unmethylated glioblastoma
Source: Neurooncol Adv. 2024 Aug 28;6(1):vdae150. doi: 10.1093/noajnl/vdae150 (PMC11450402; doi:10.1093/noajnl/vdae150)

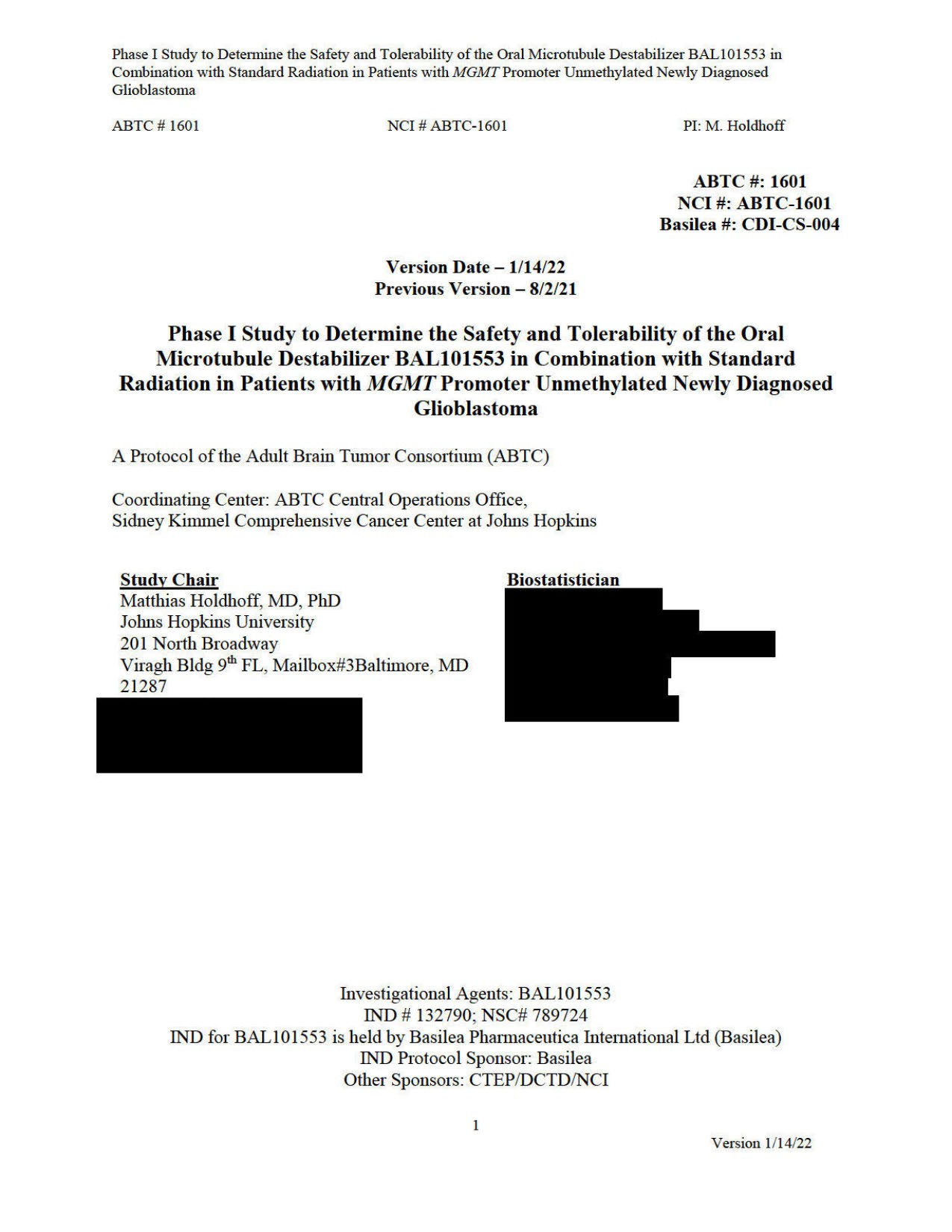


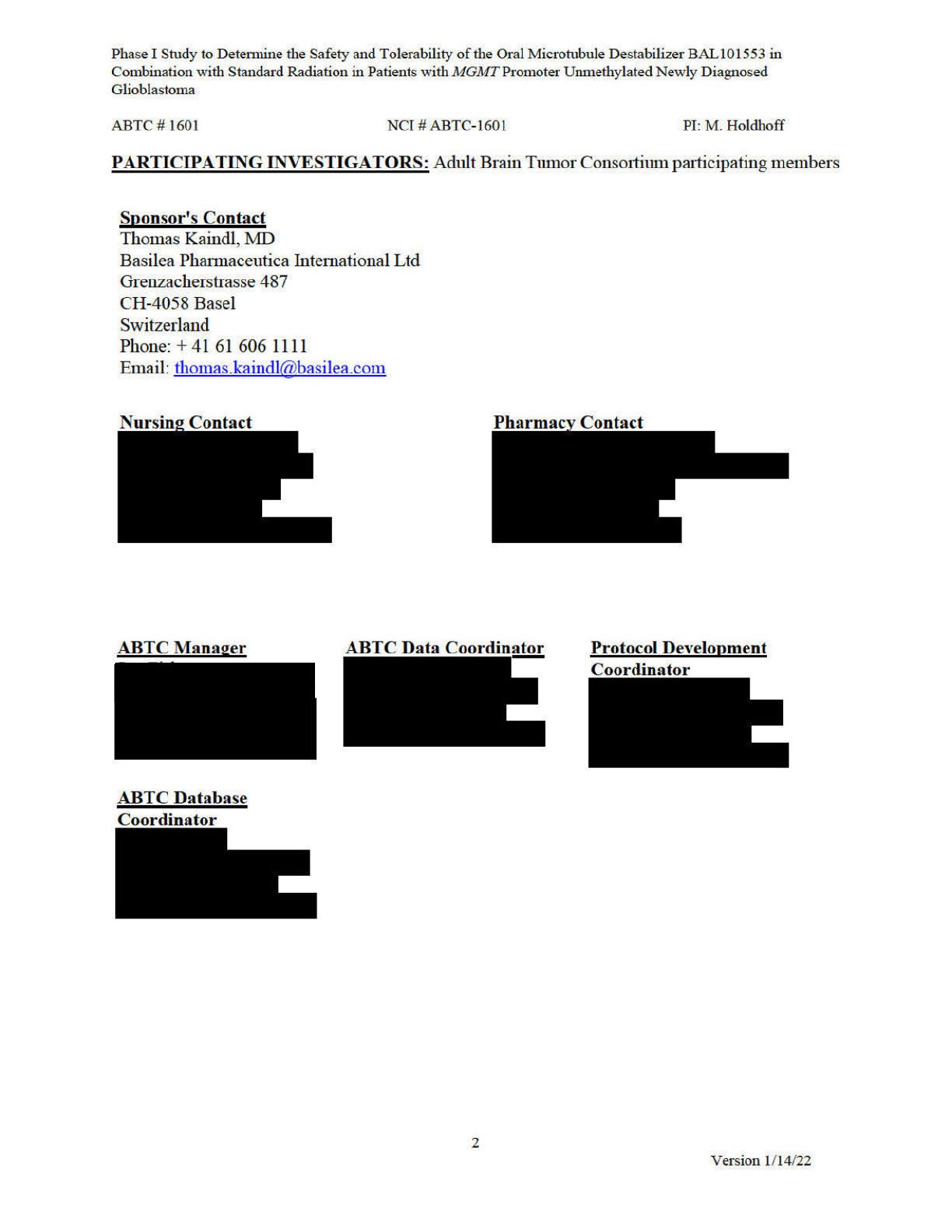


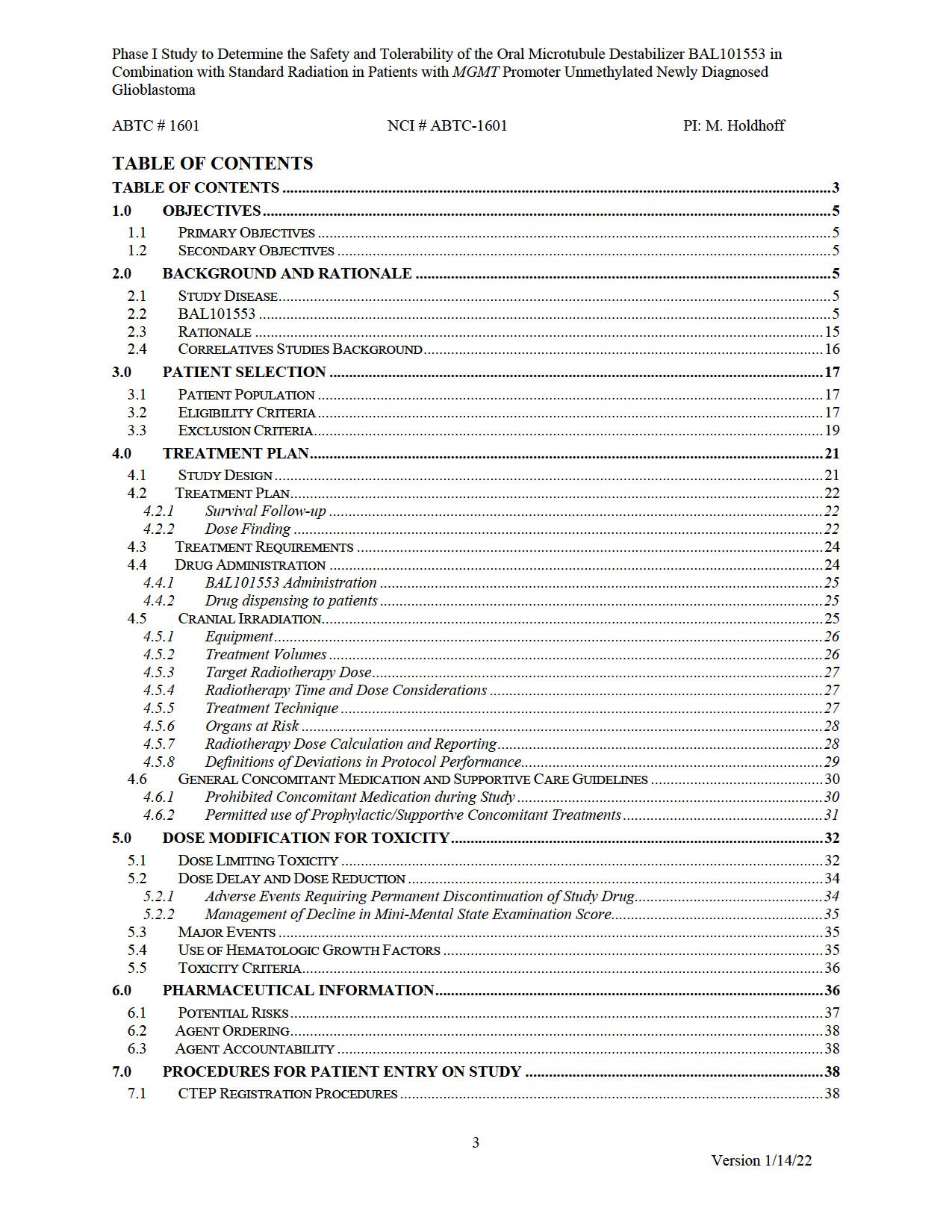


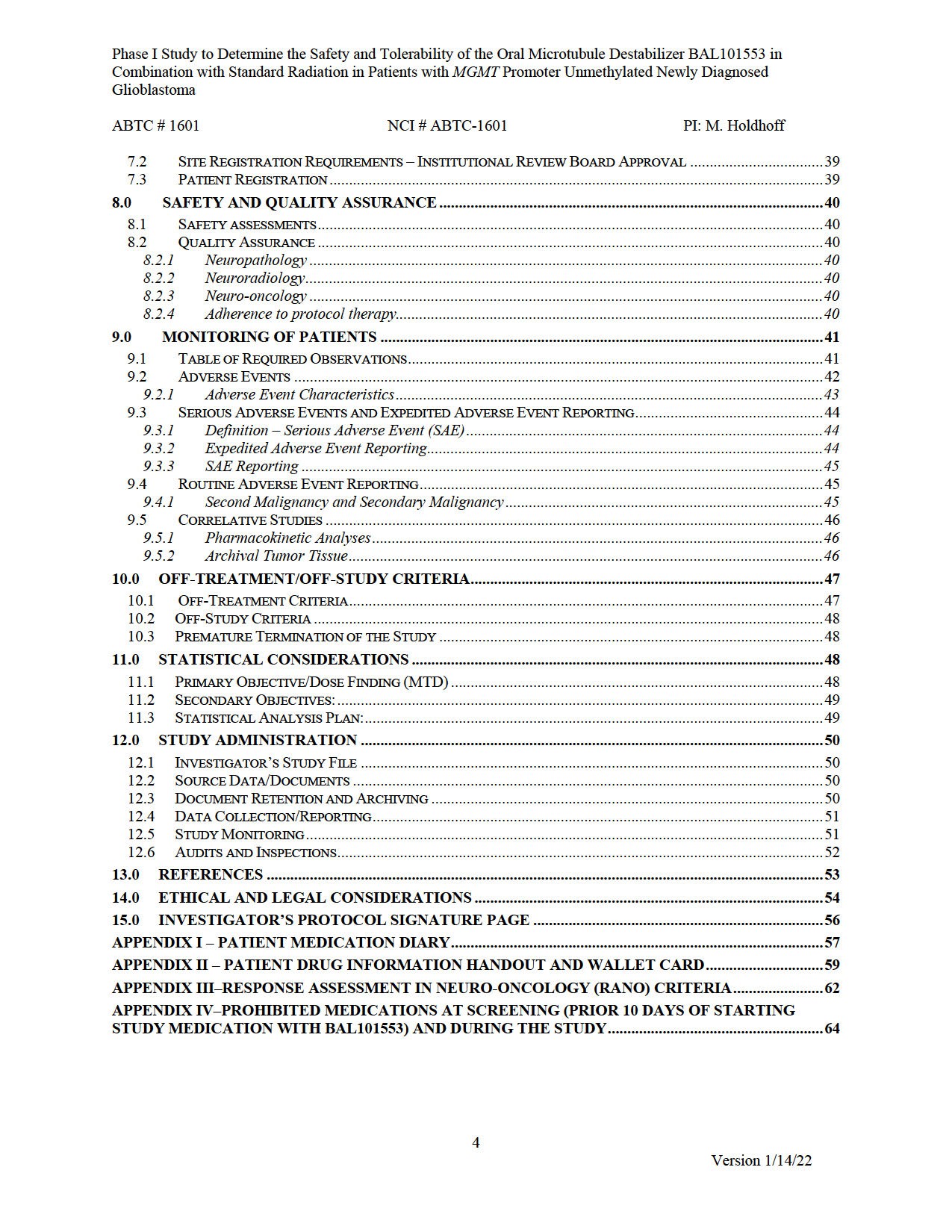


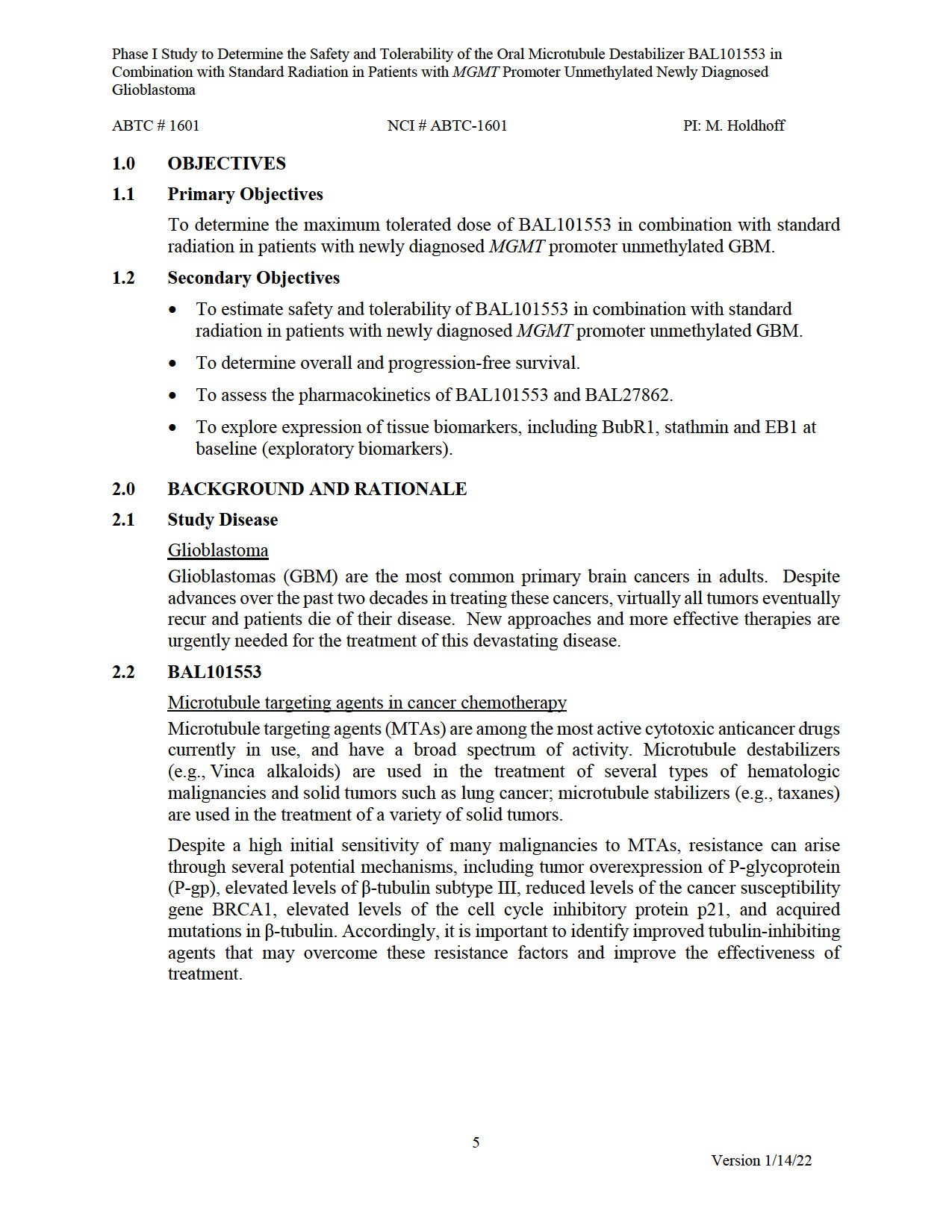


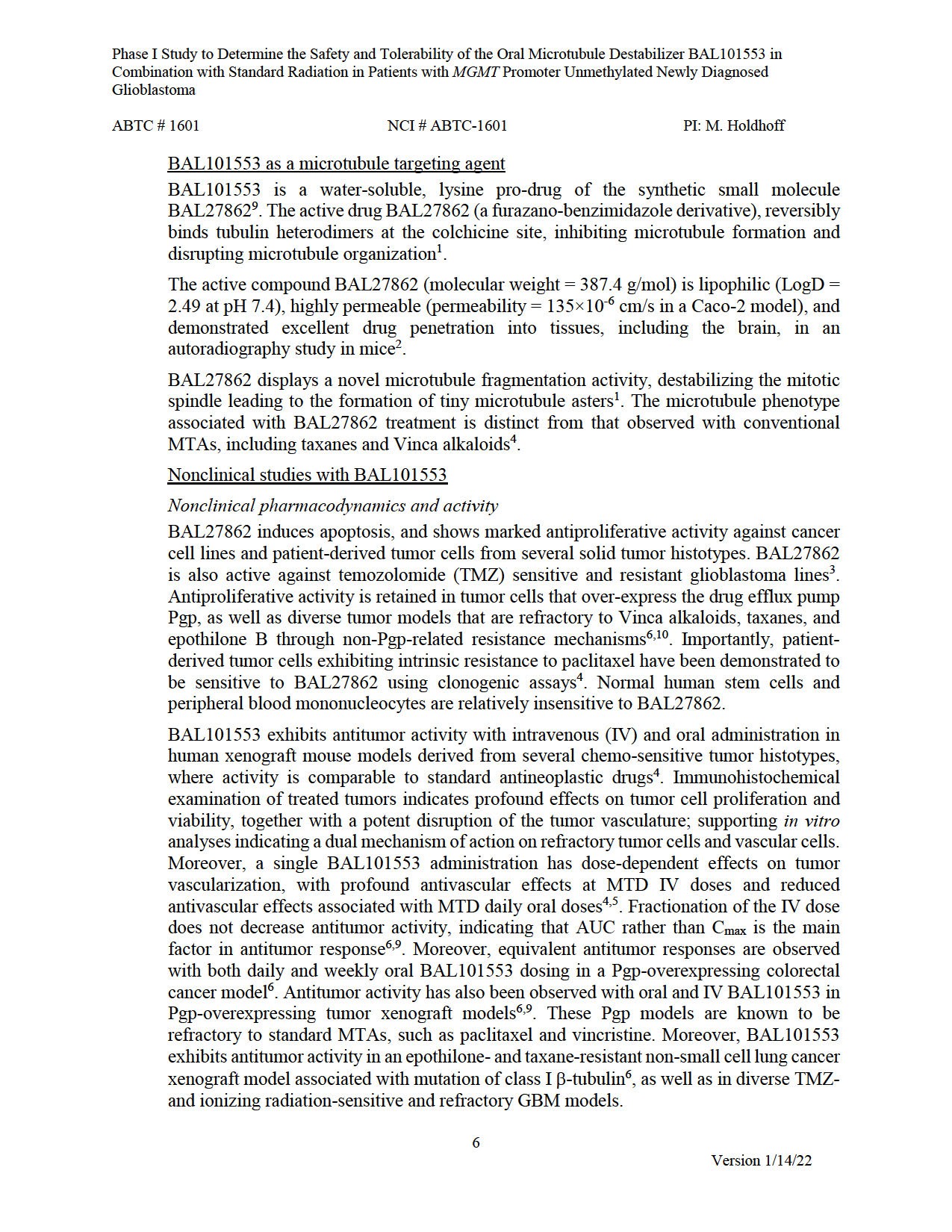


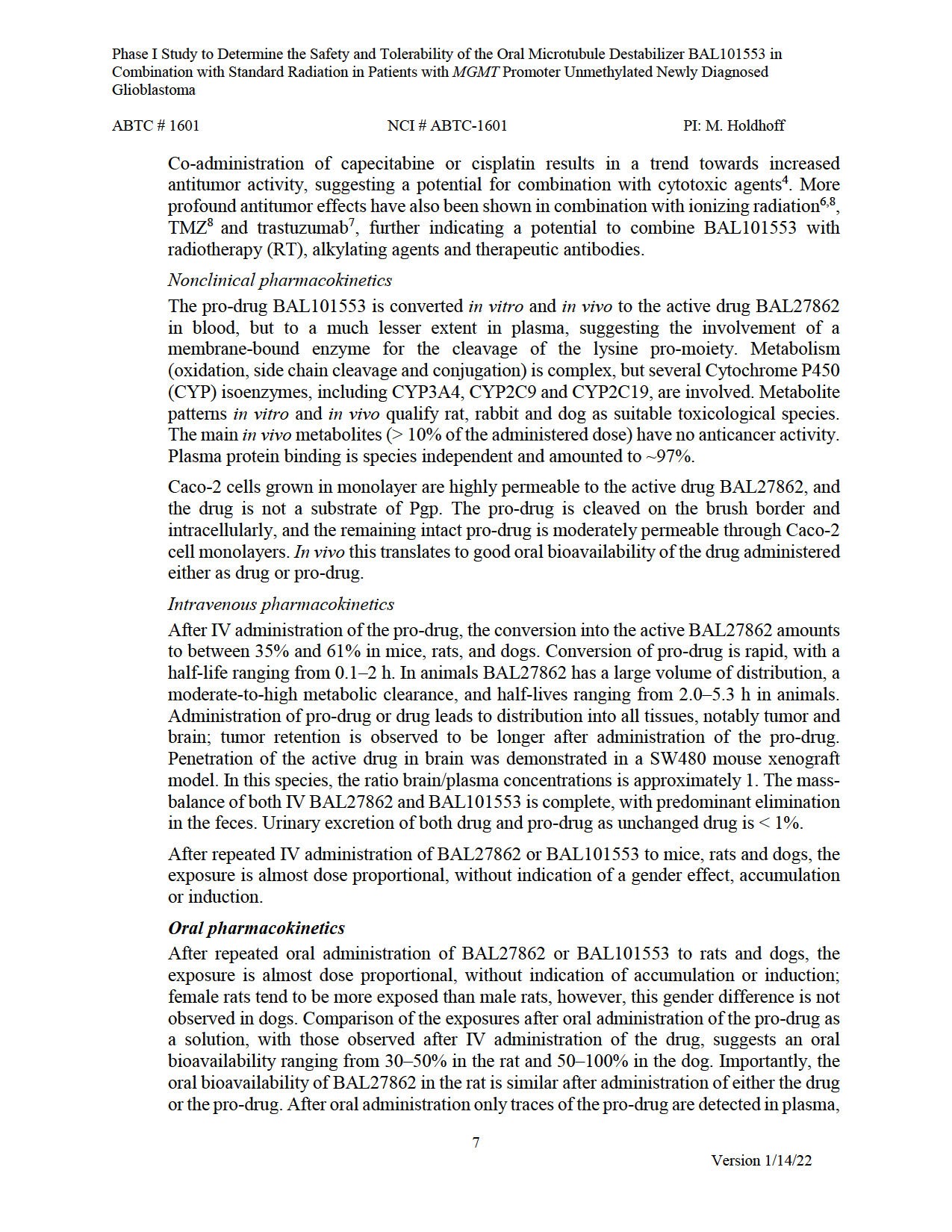


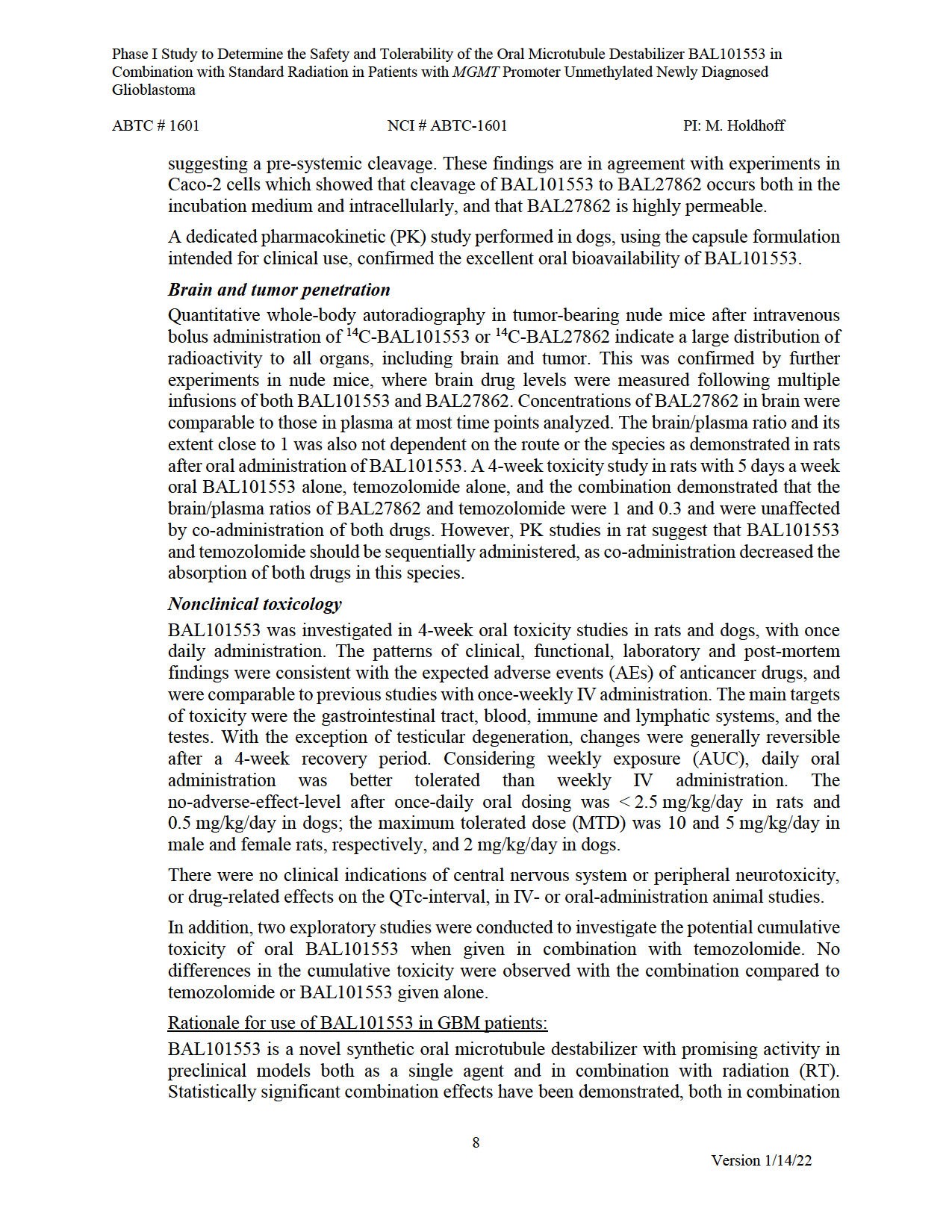


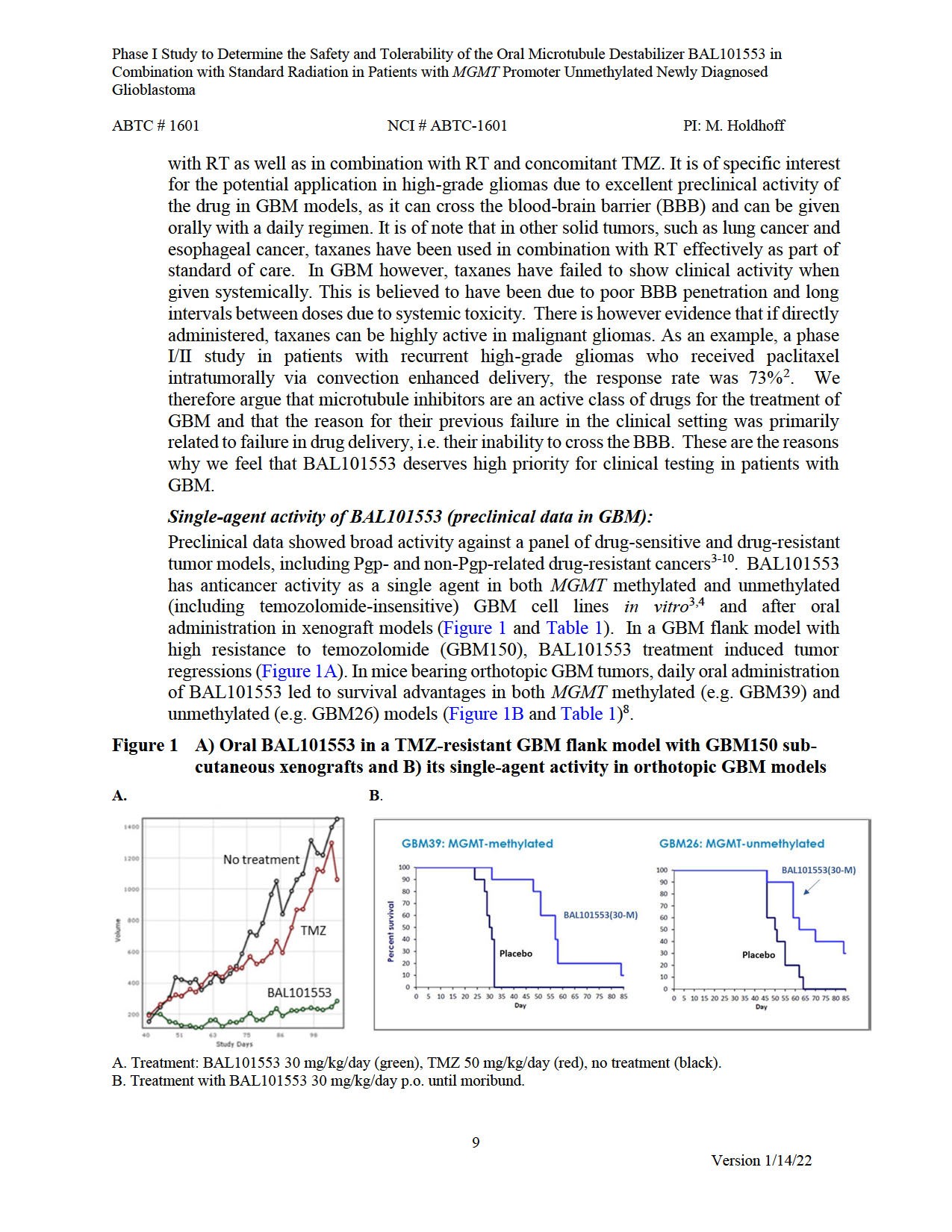


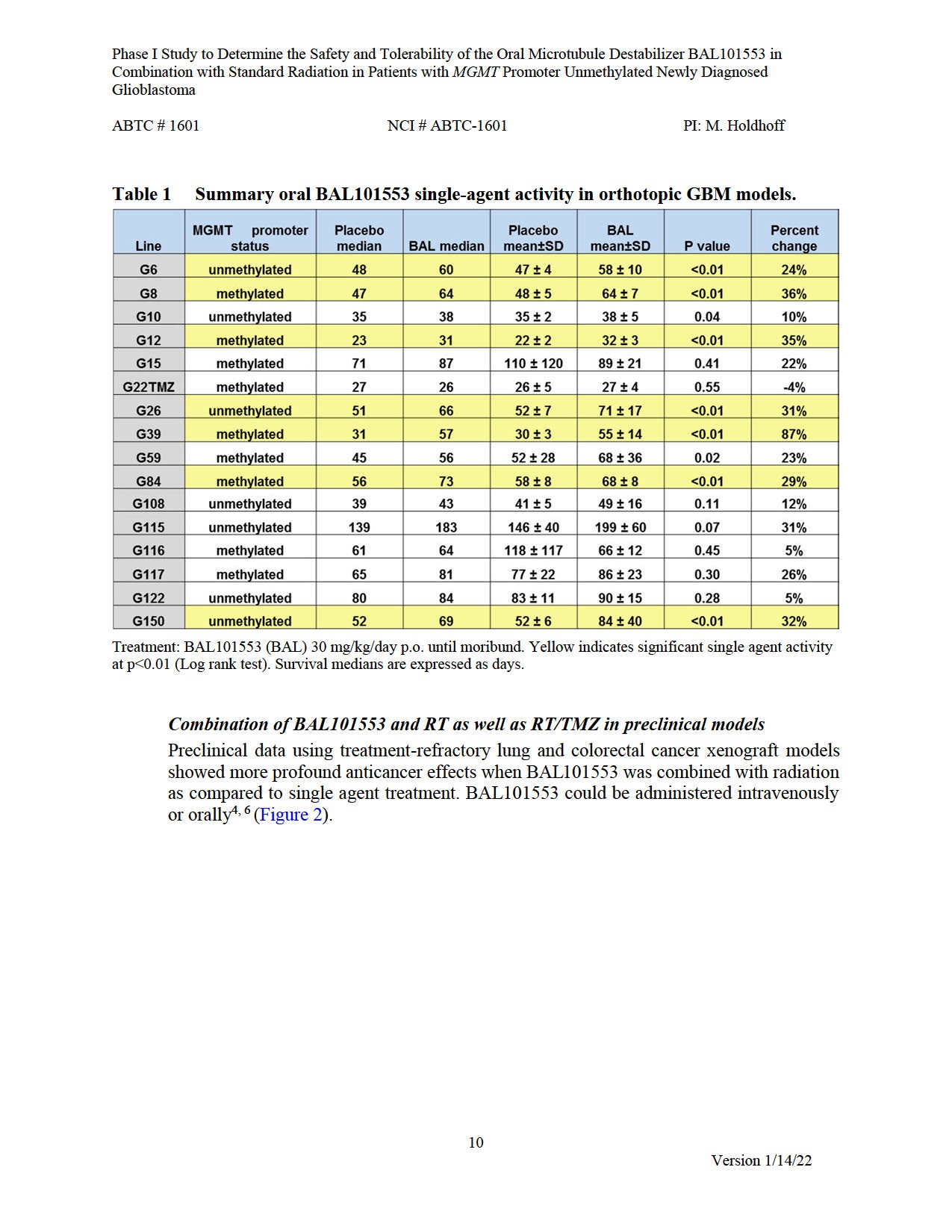


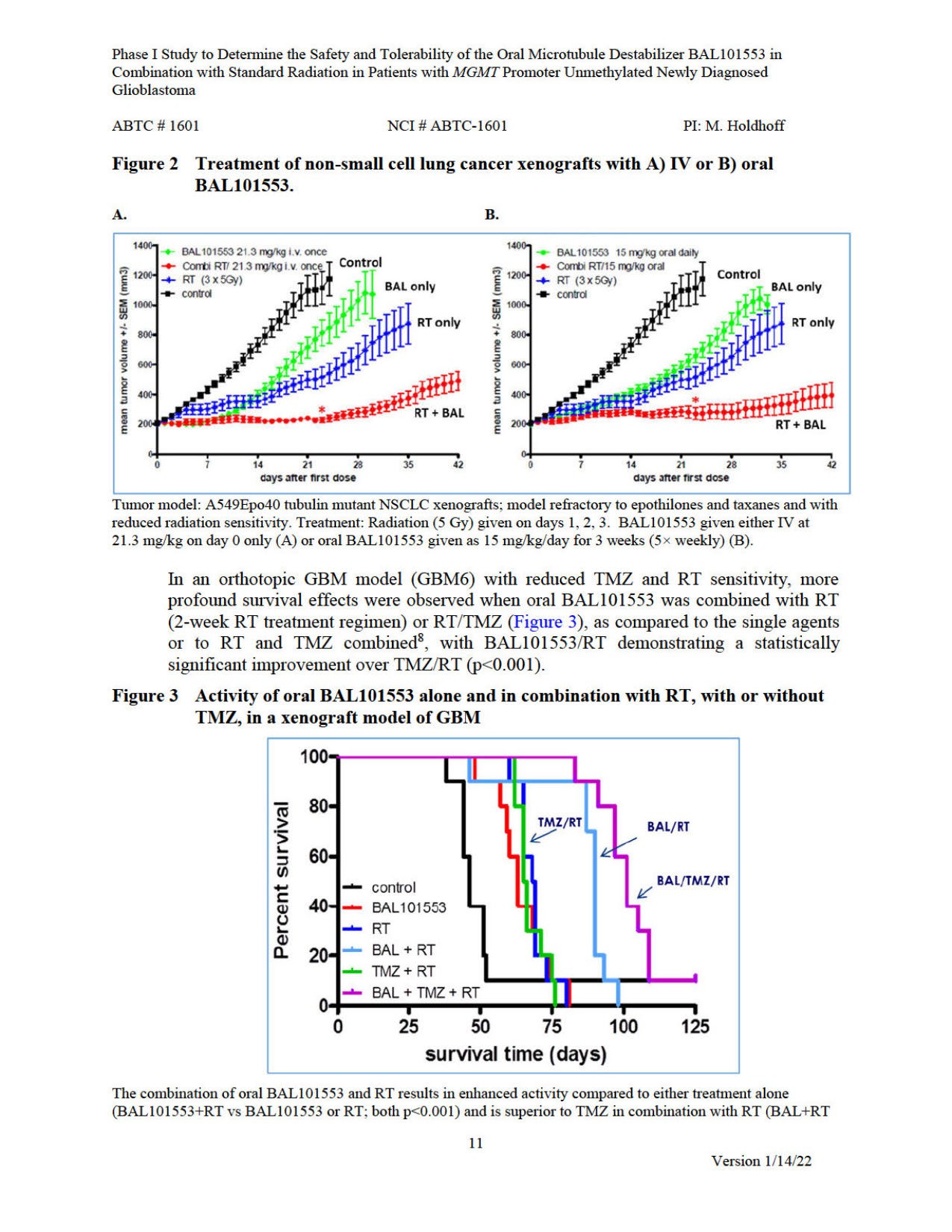


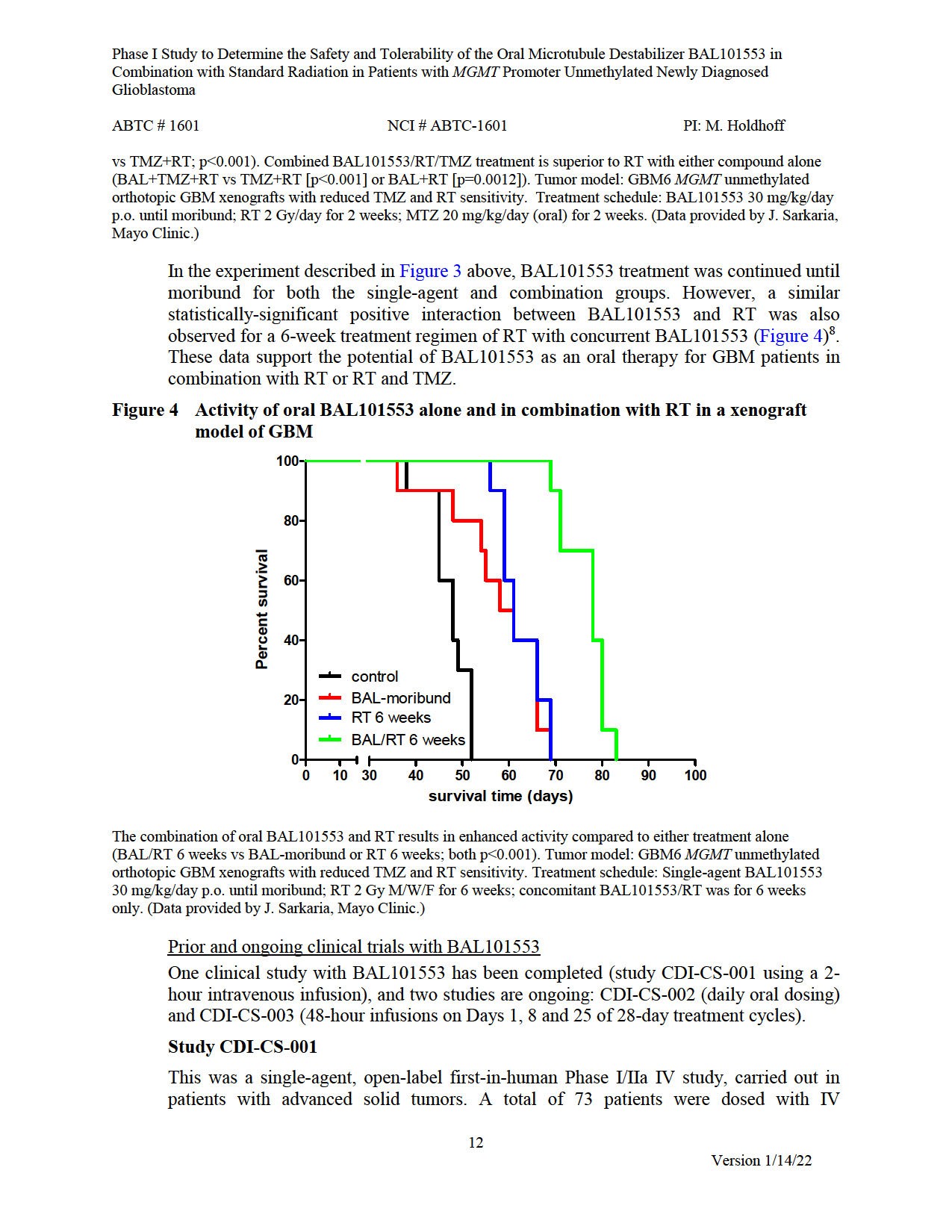


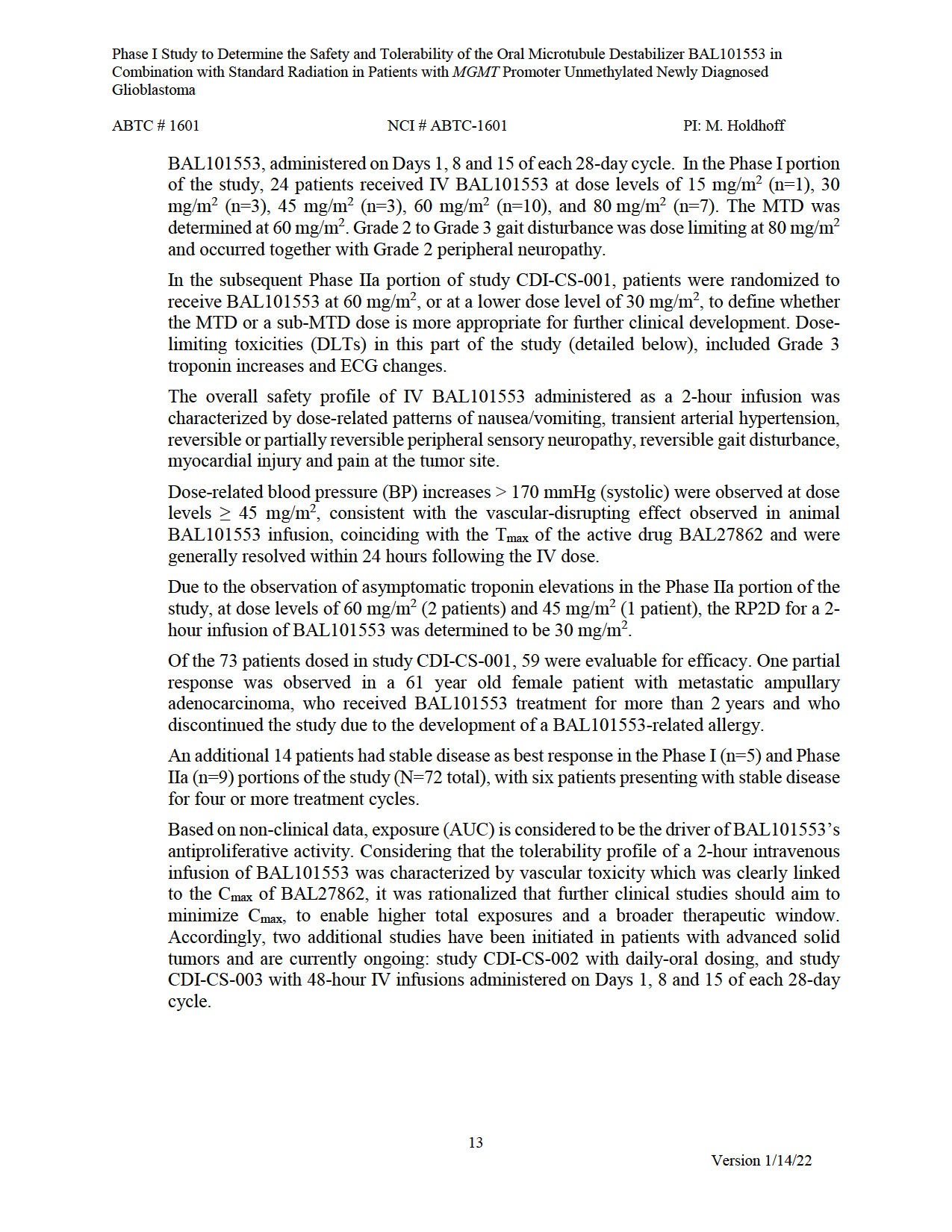


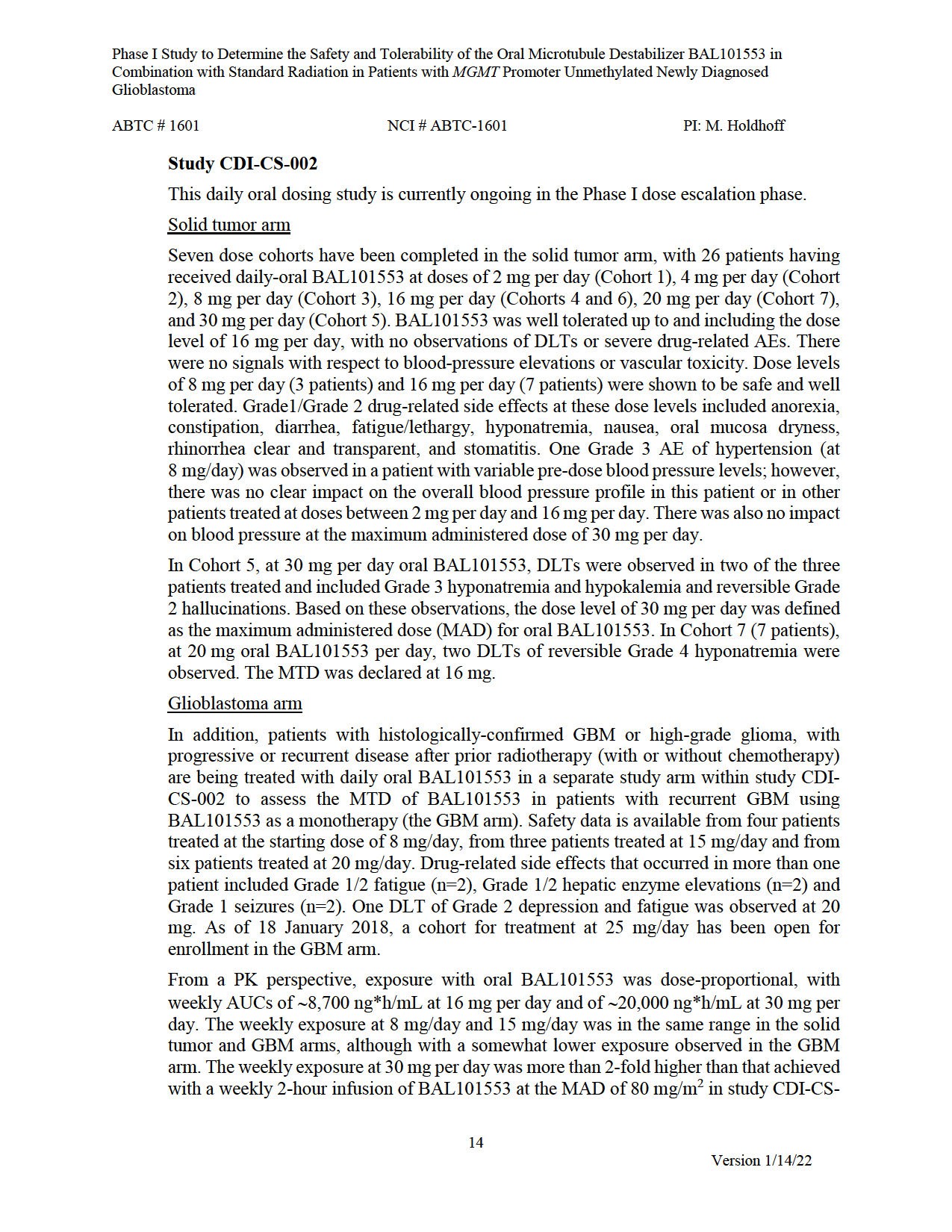


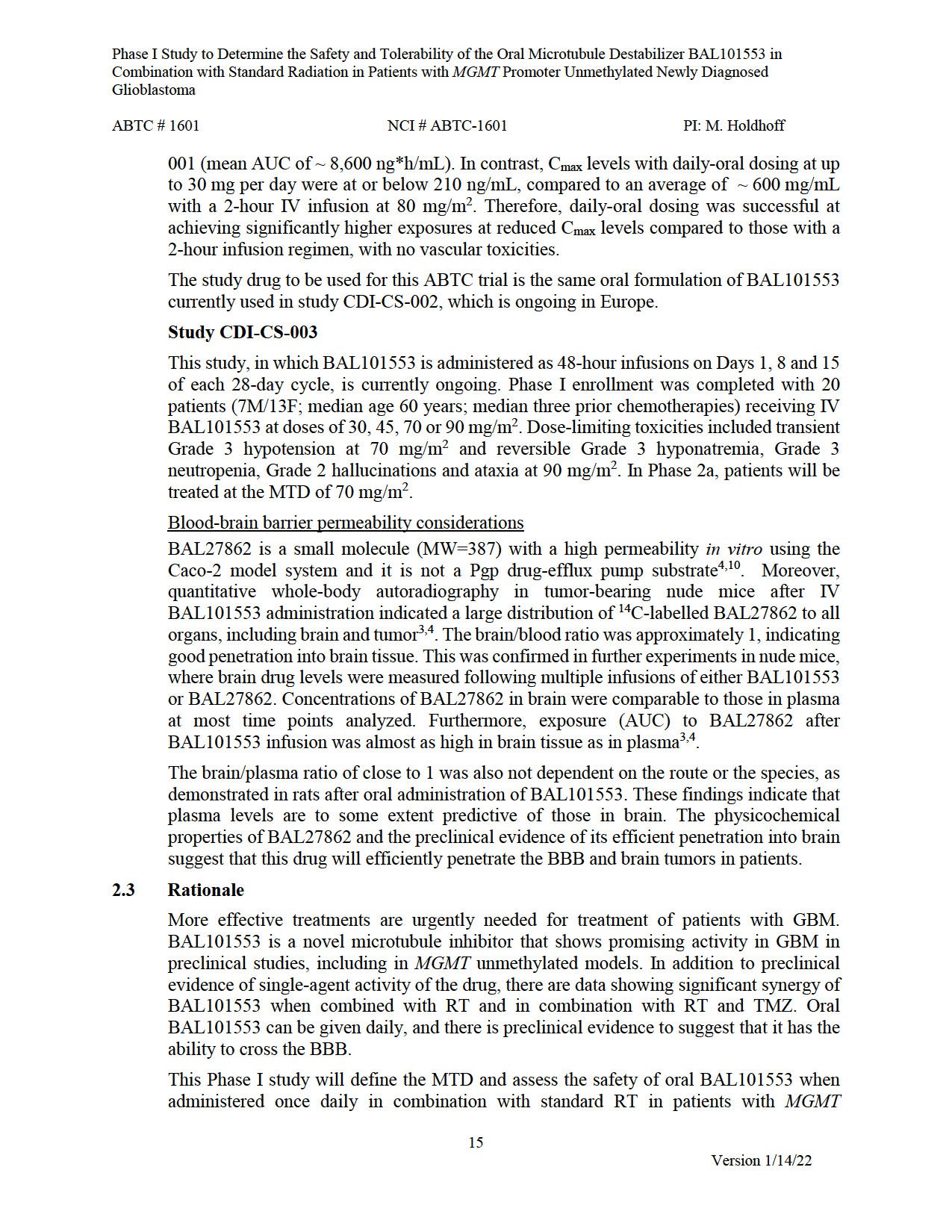


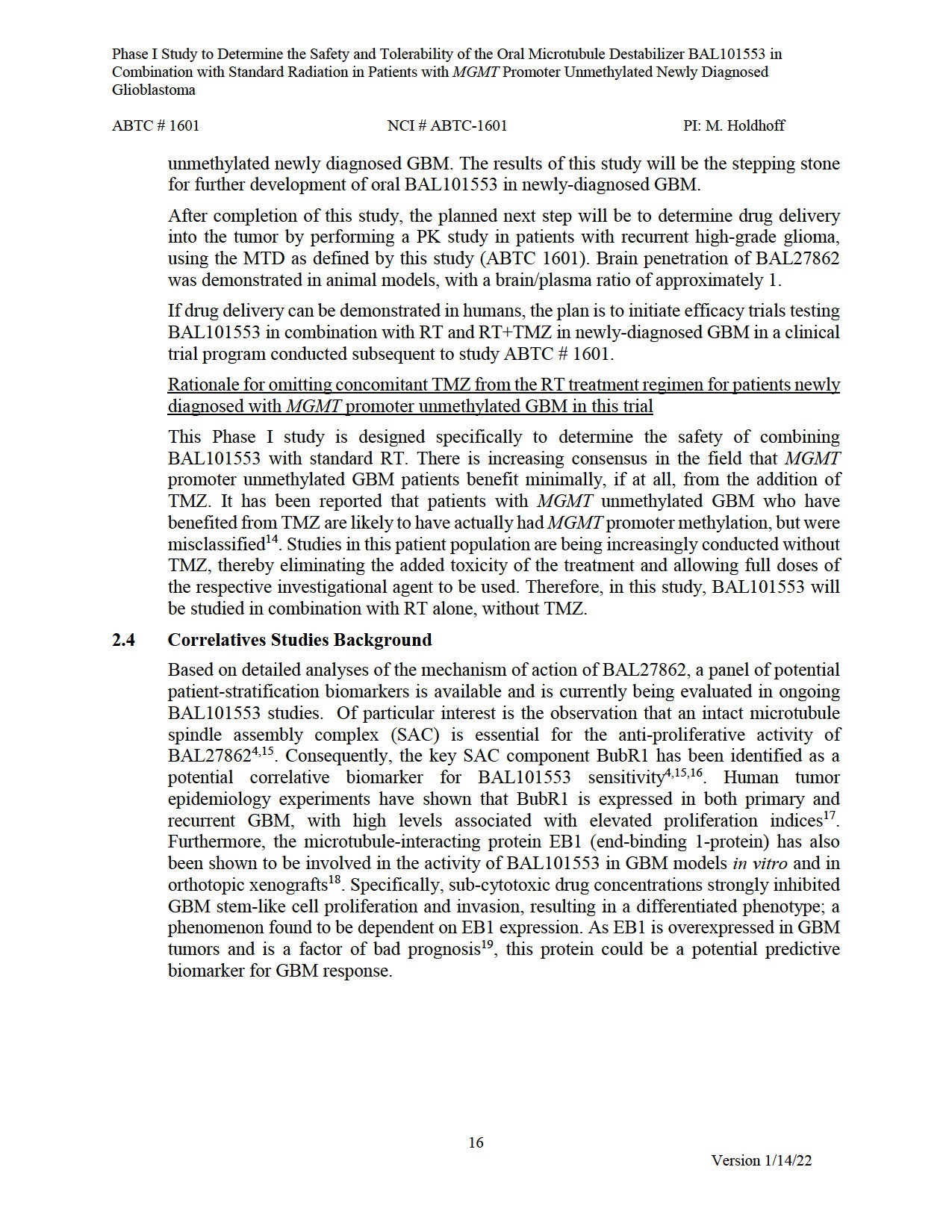


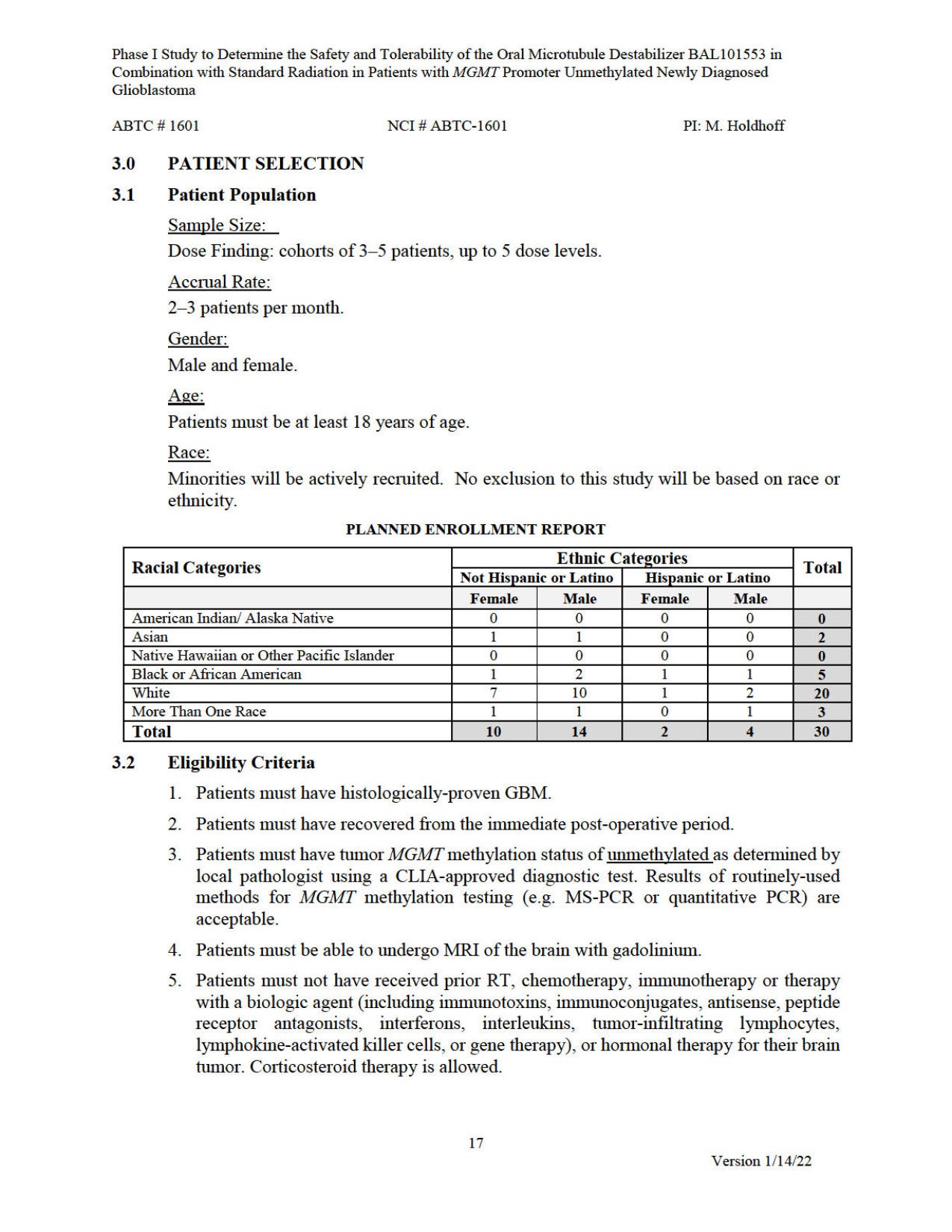


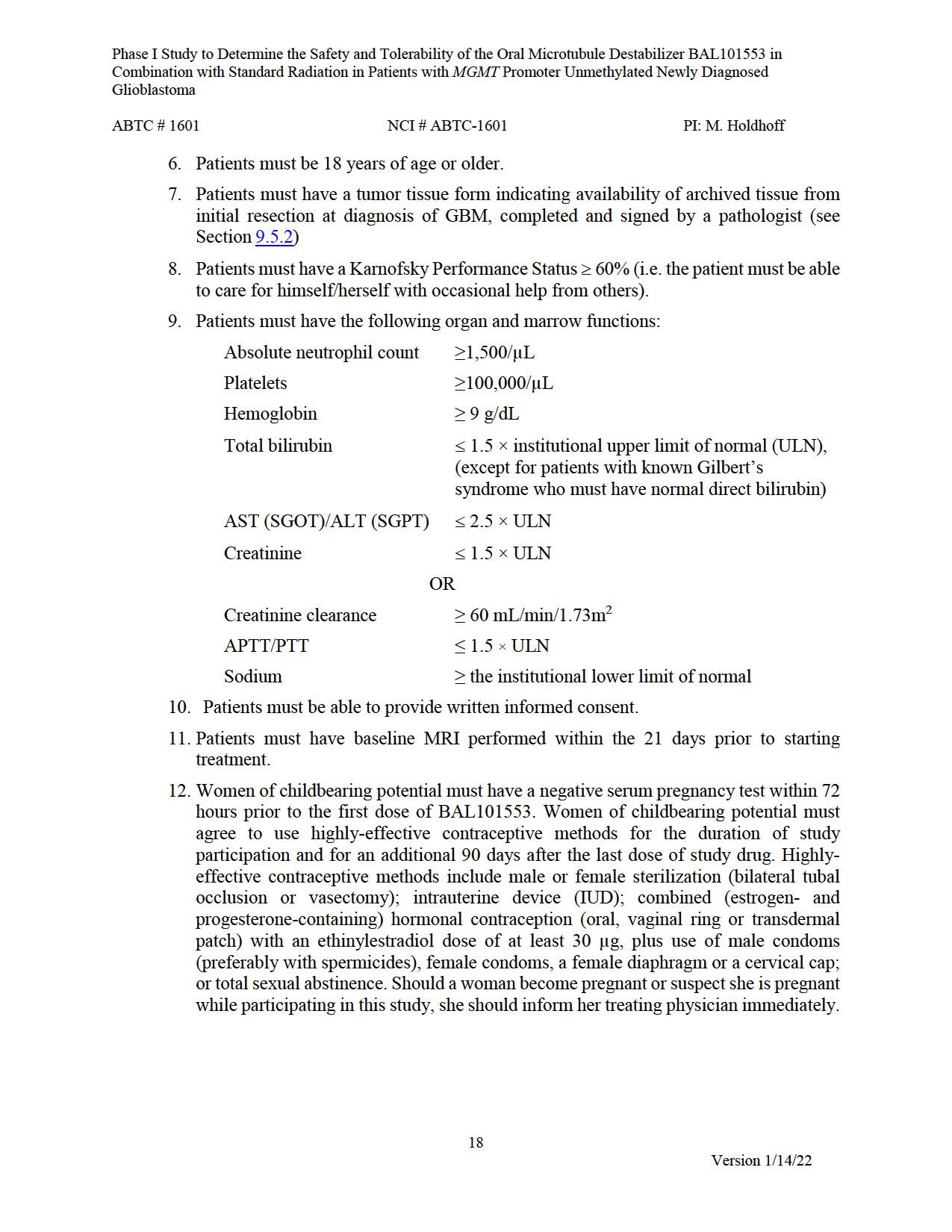


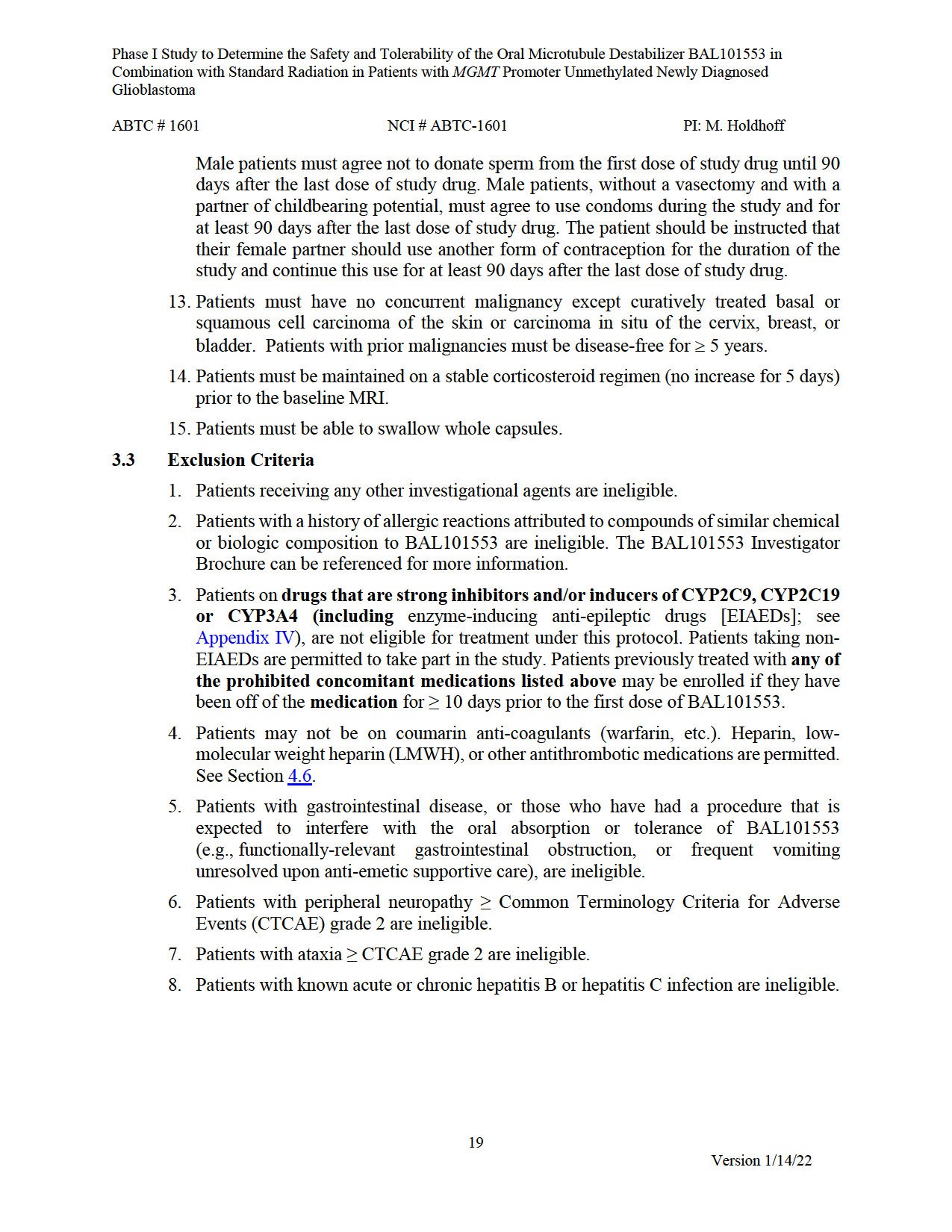


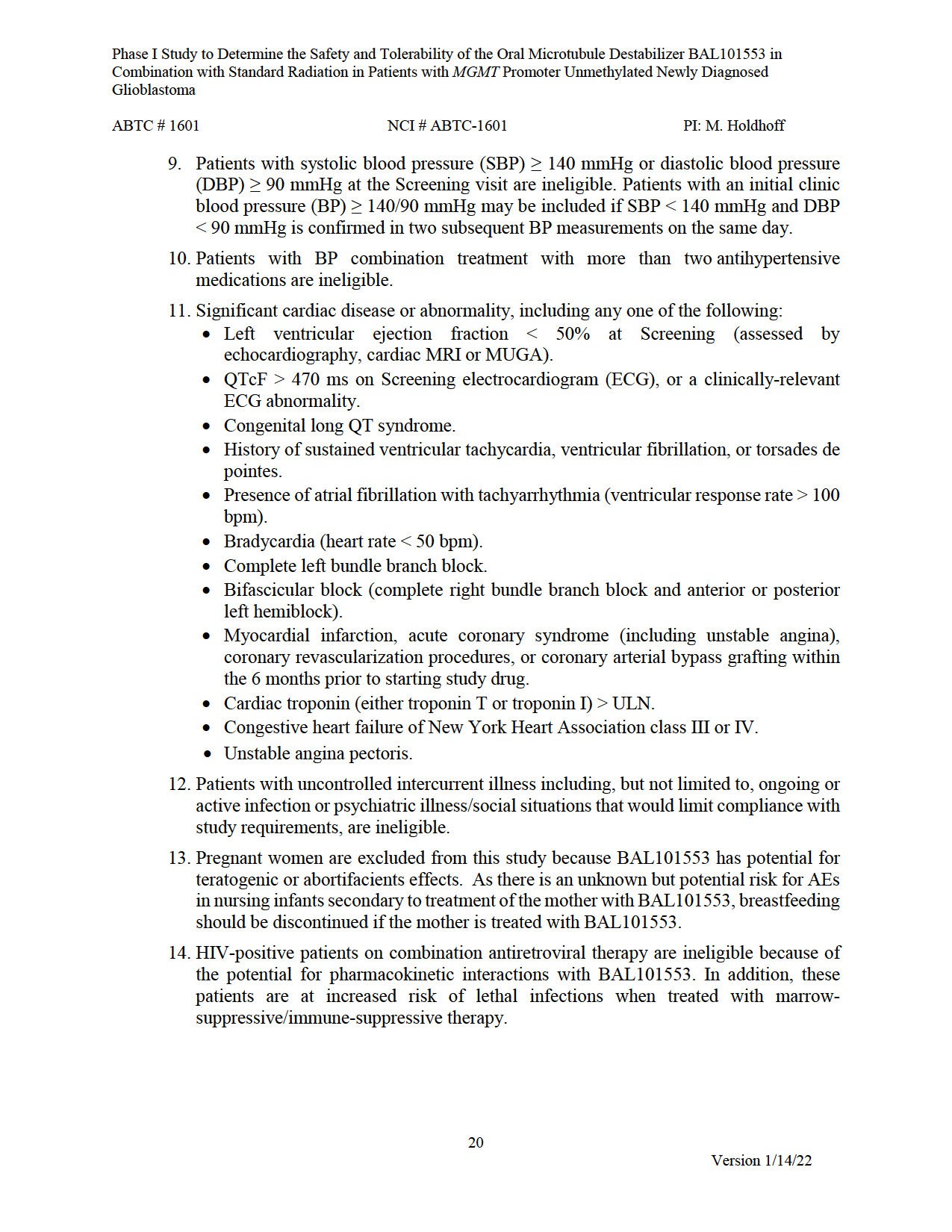


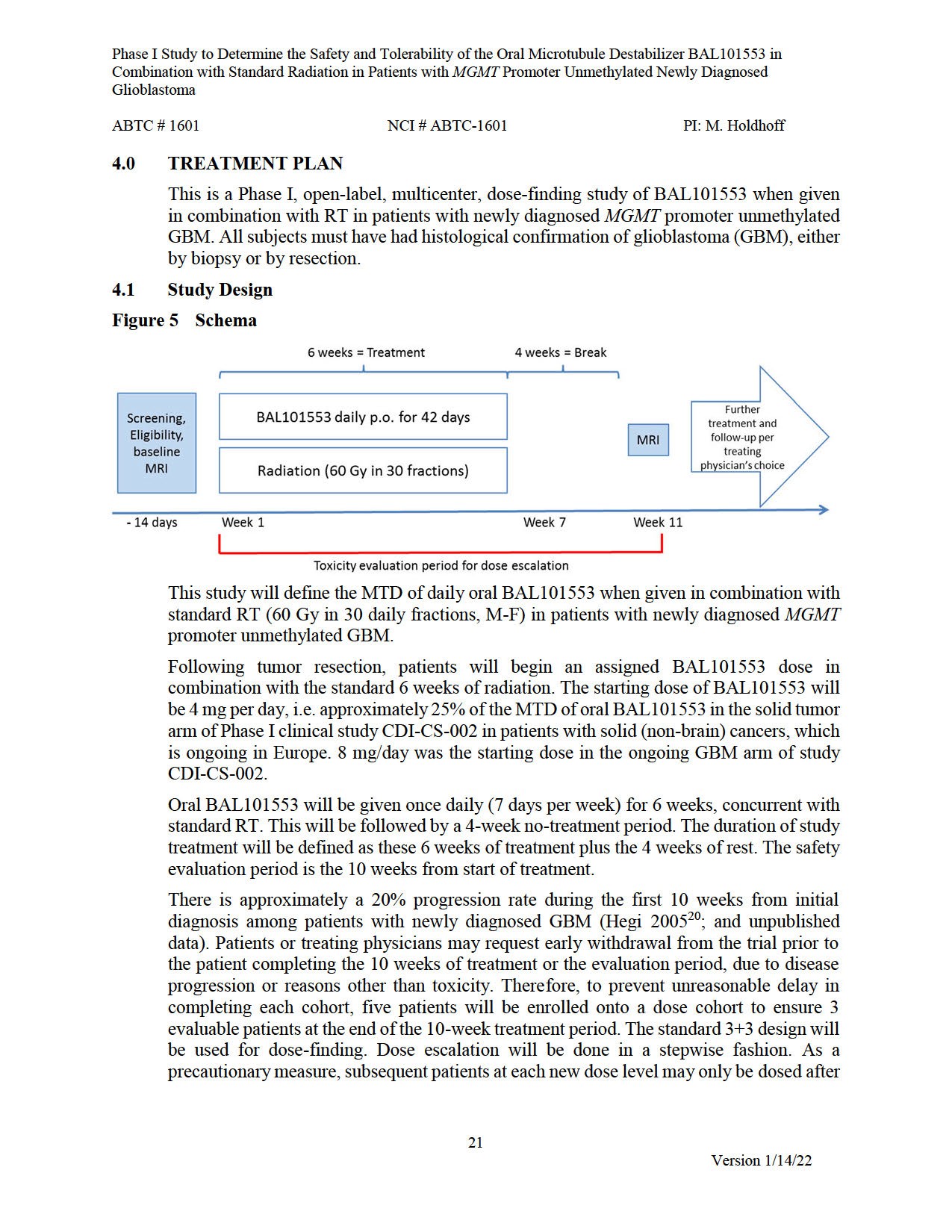


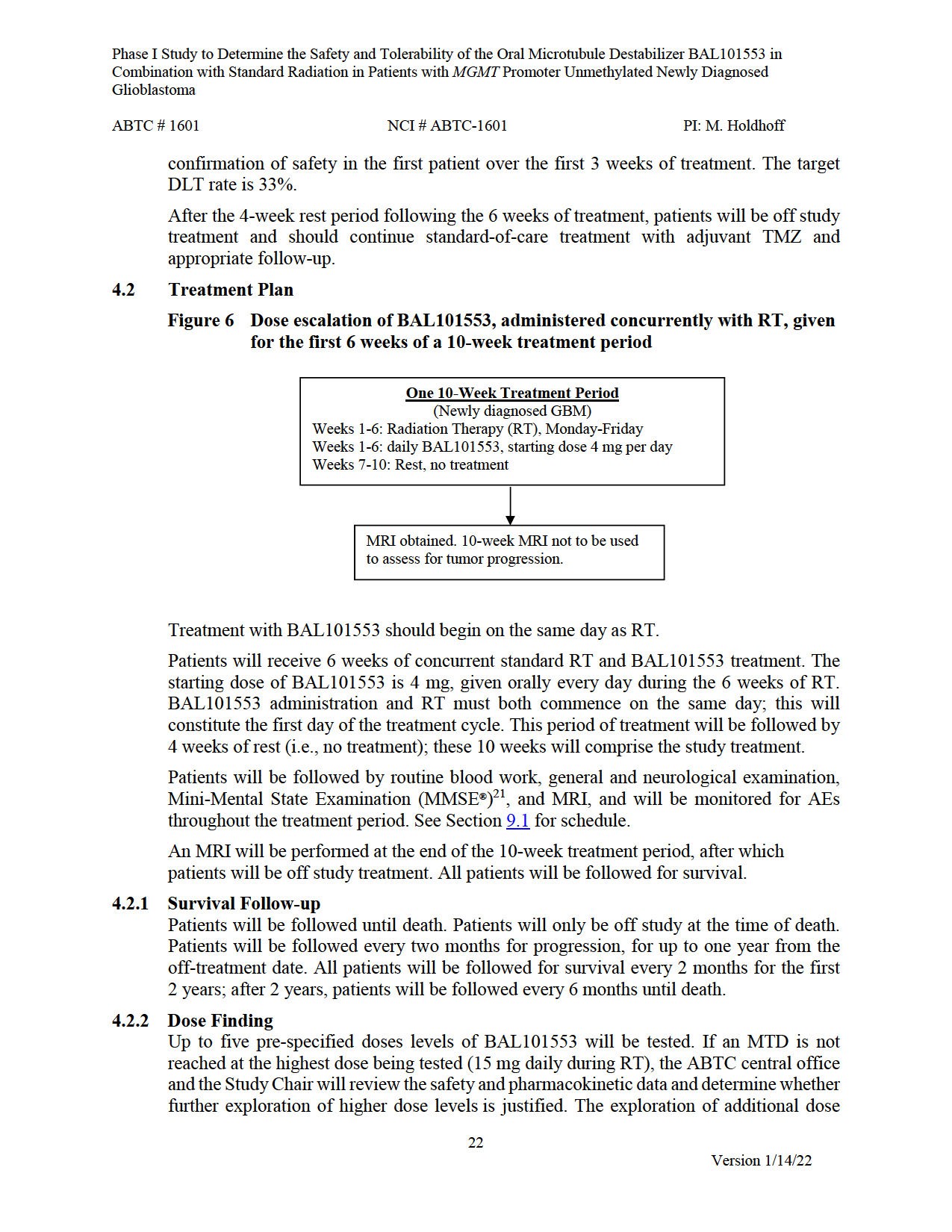


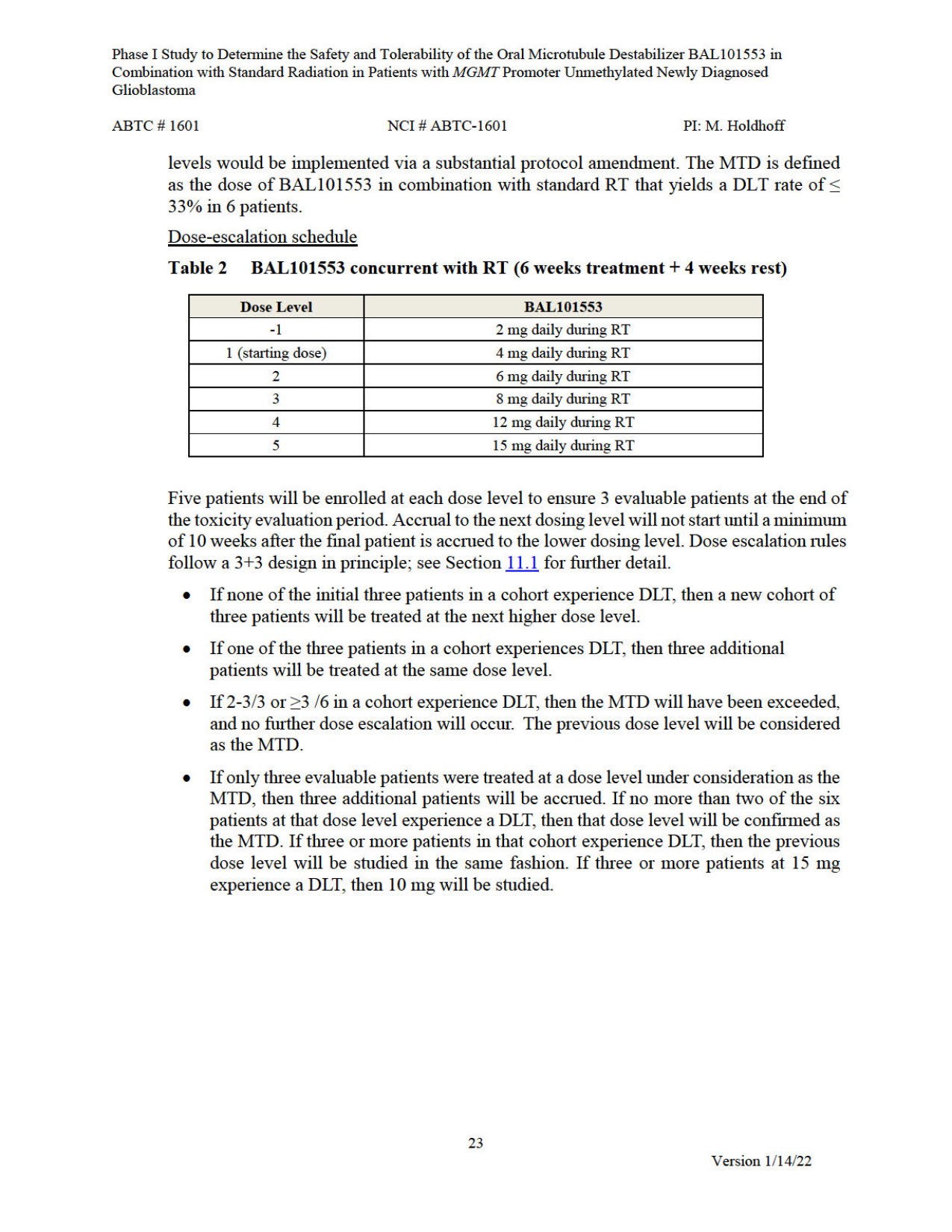


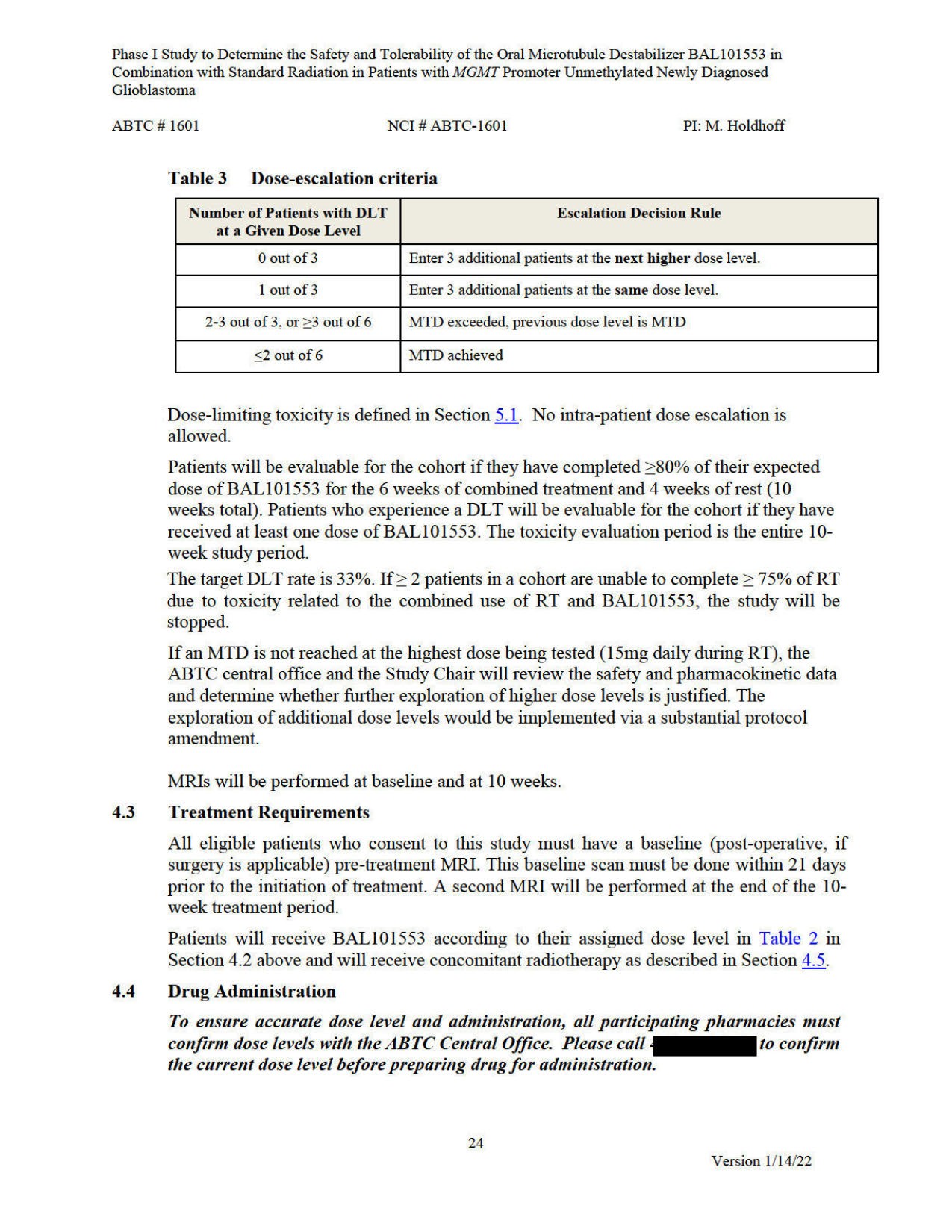


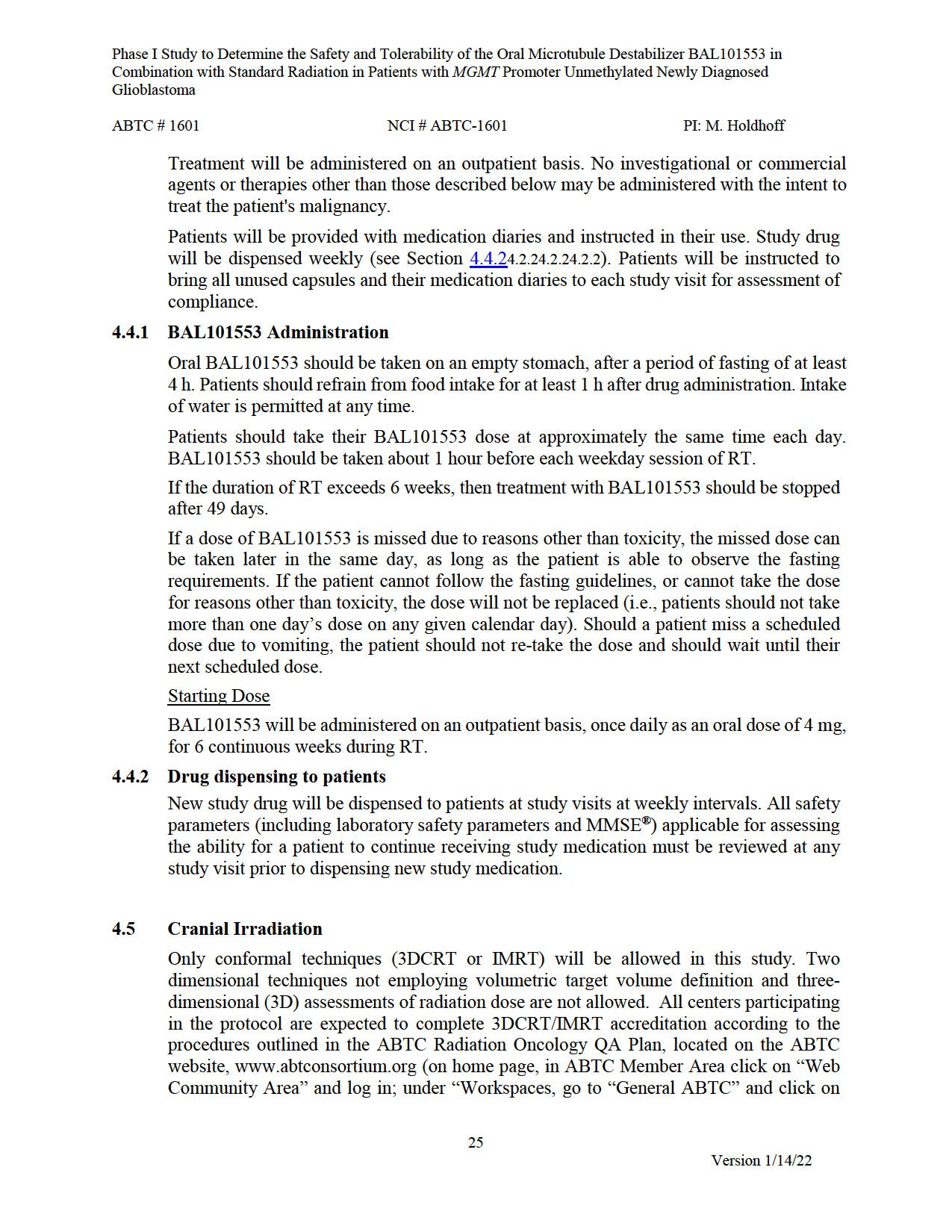


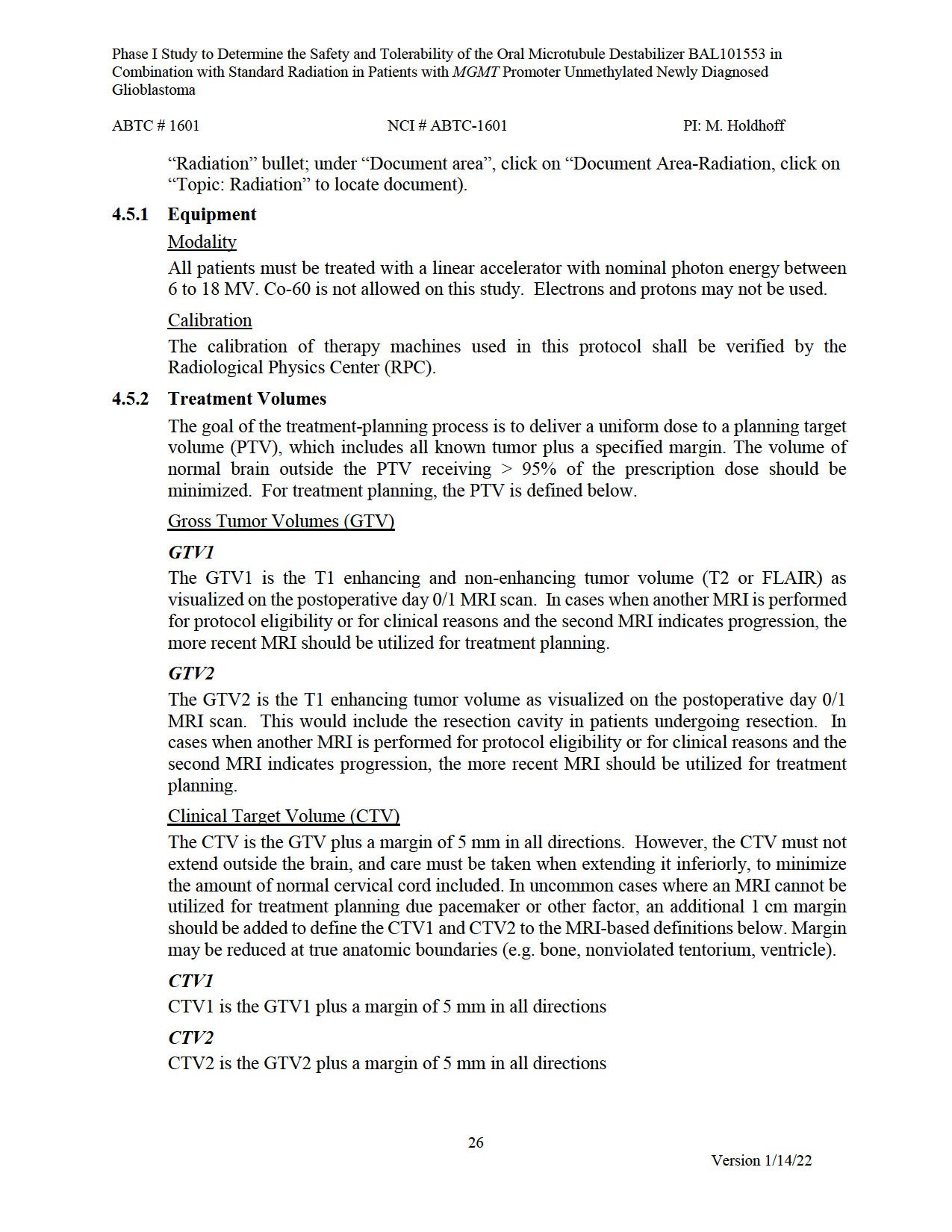


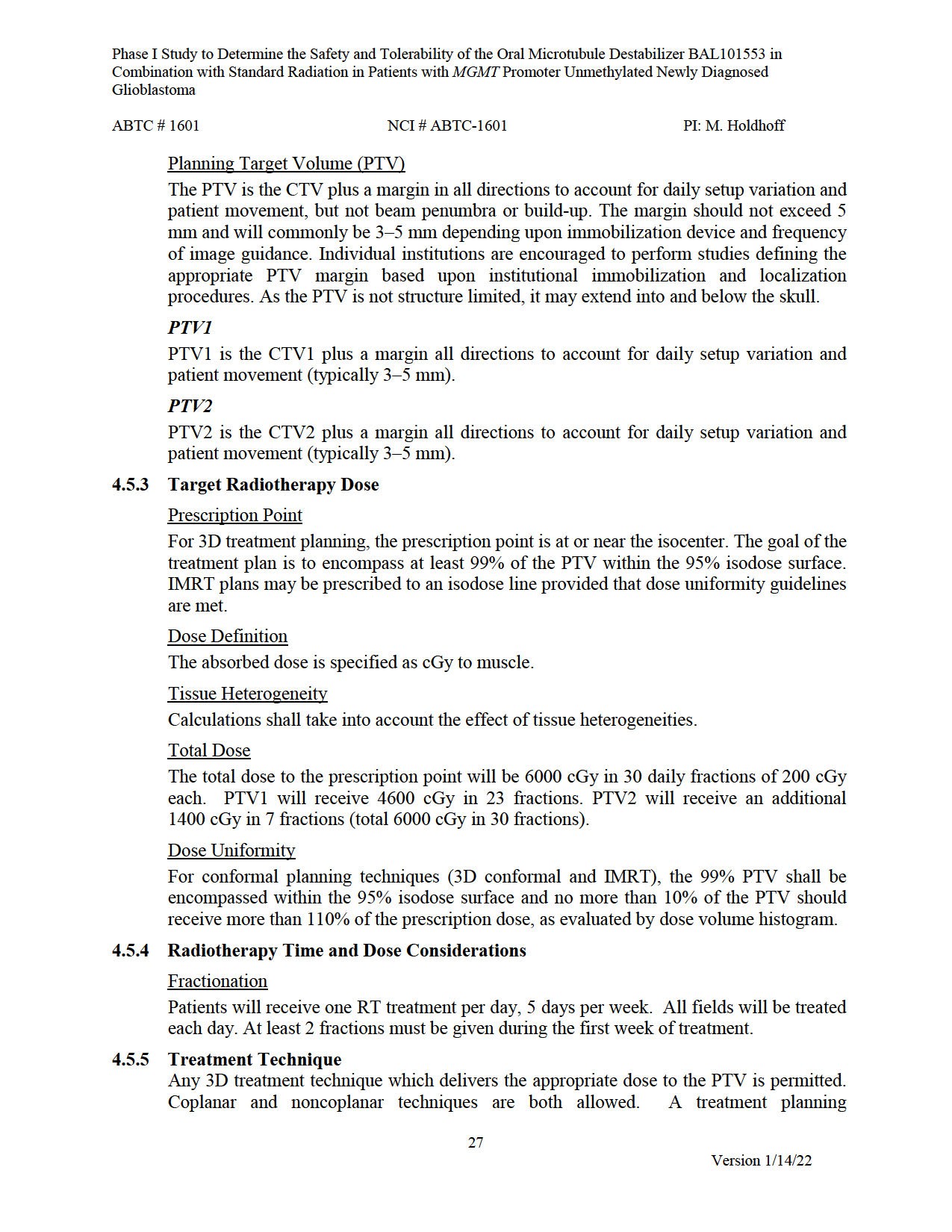


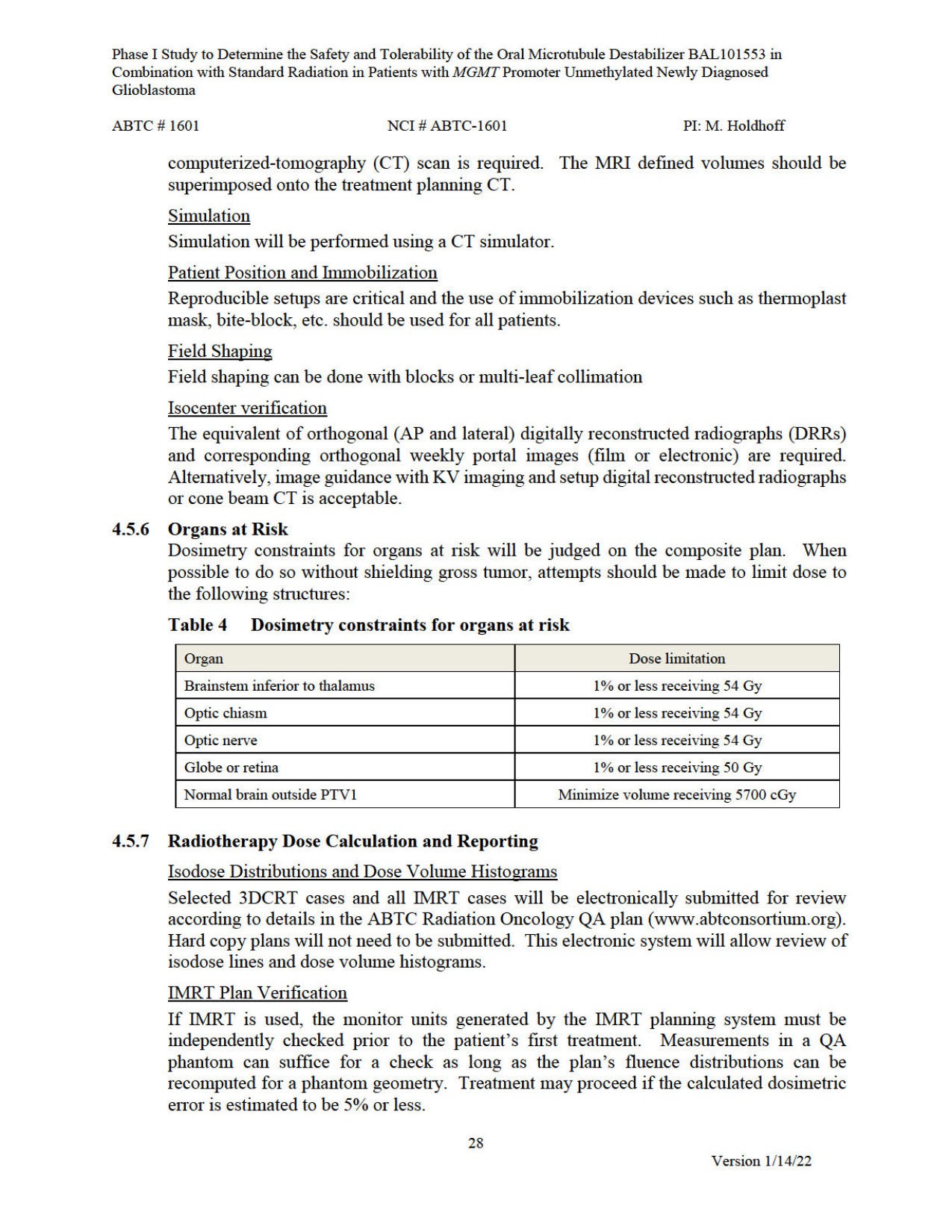


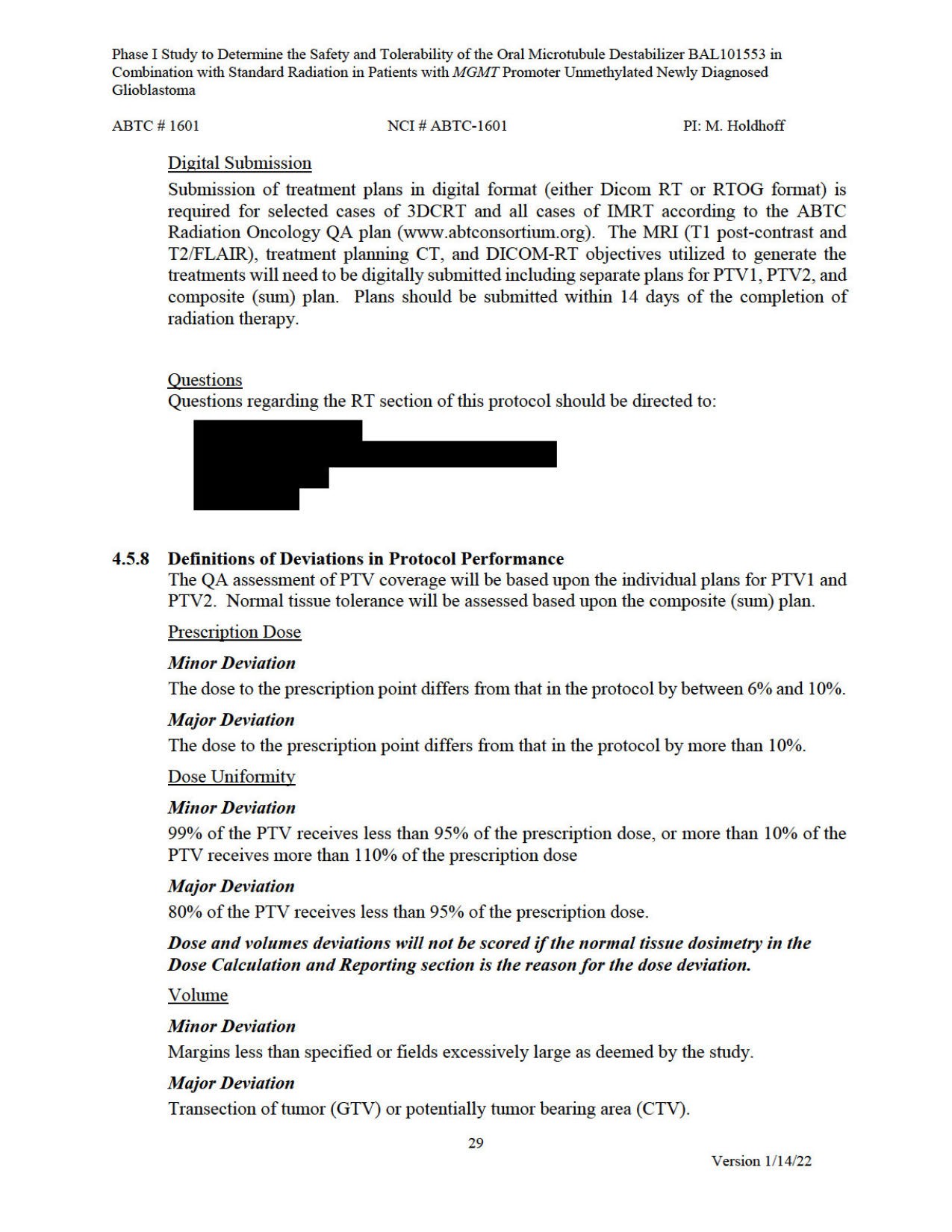


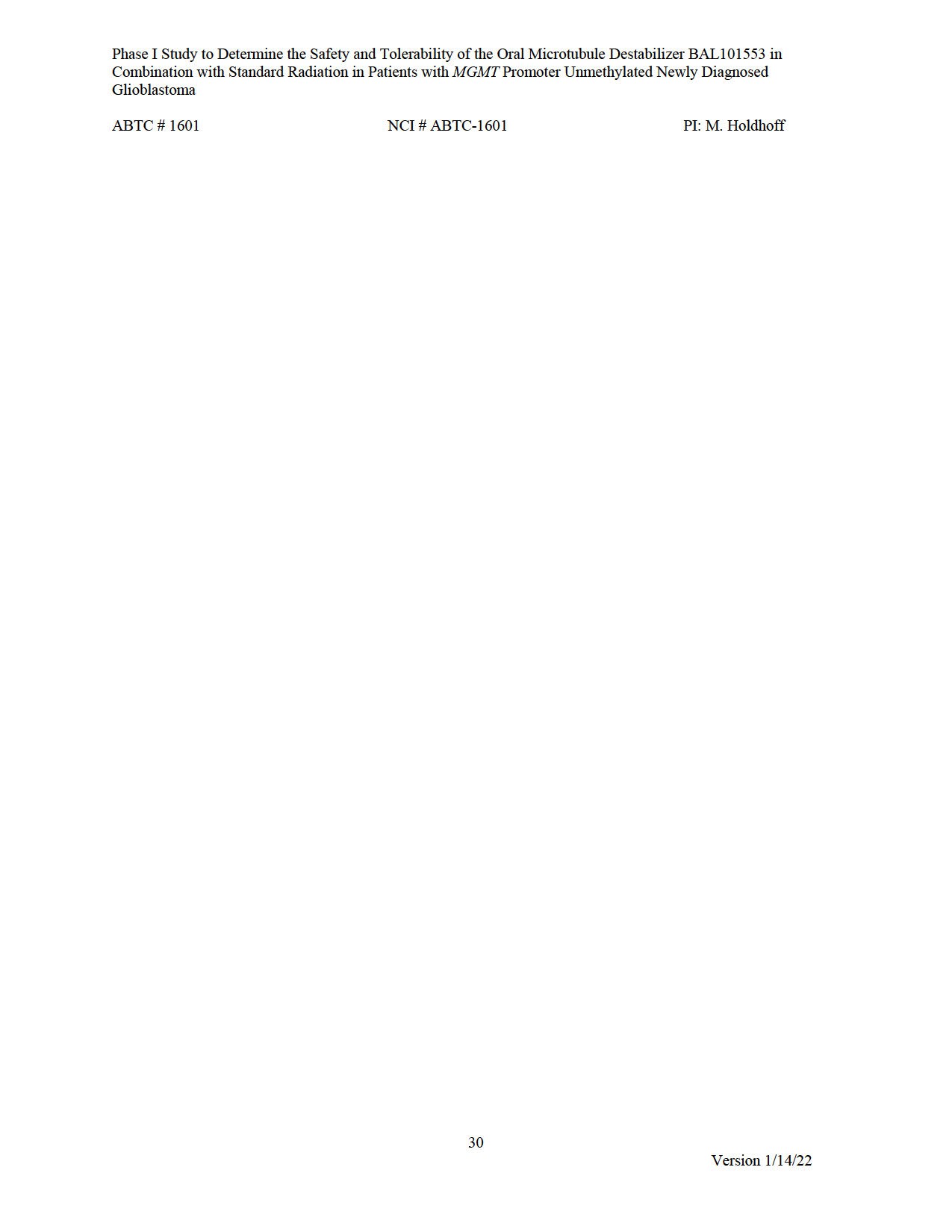


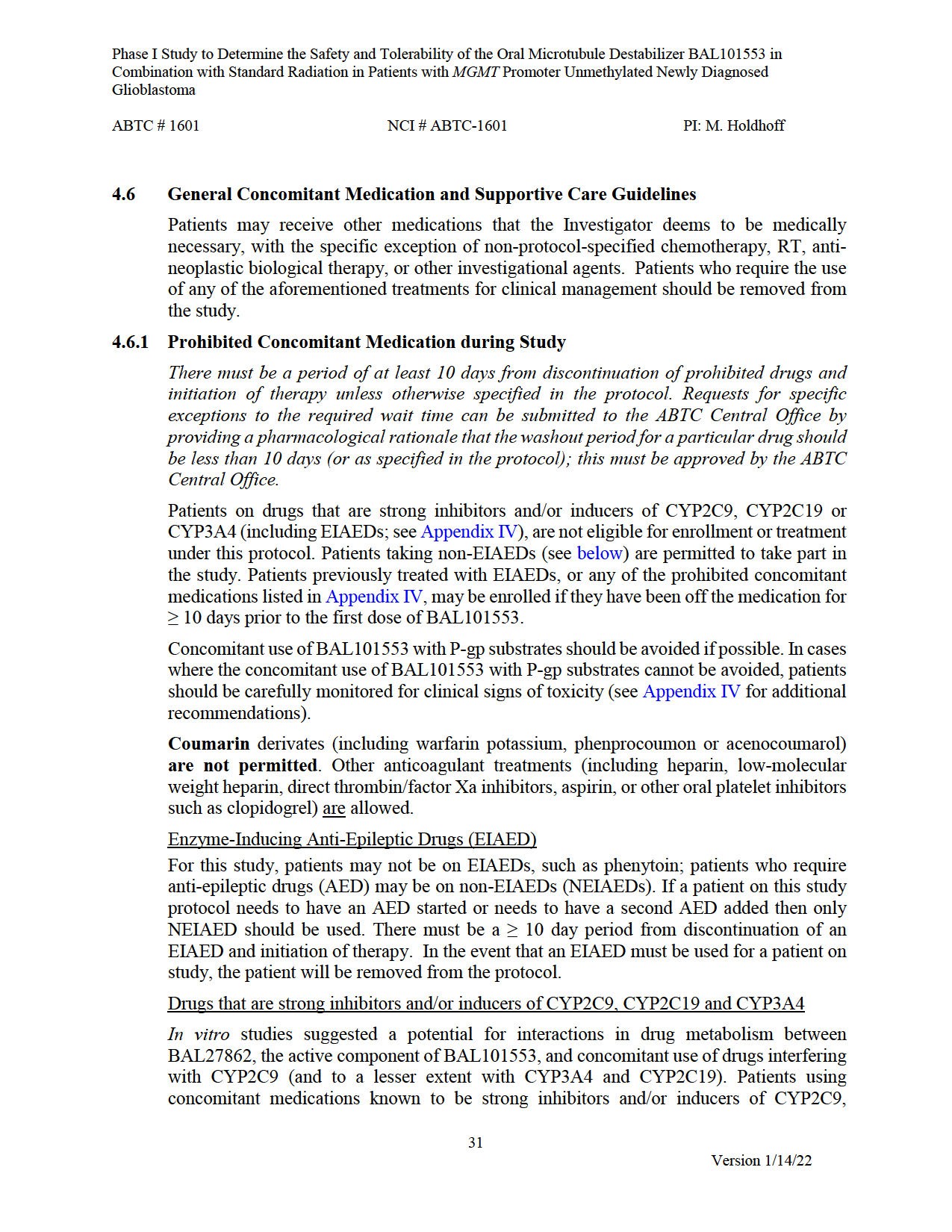


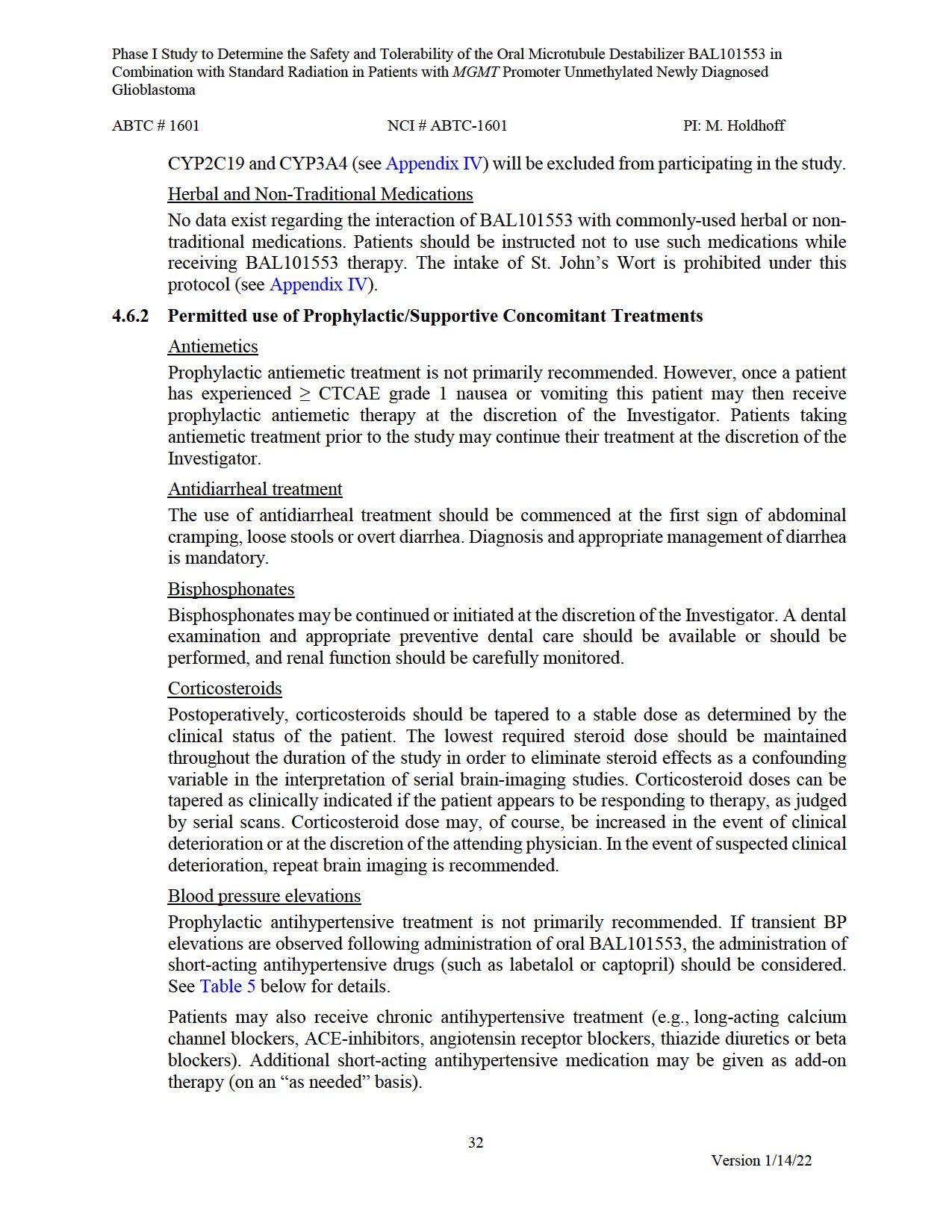


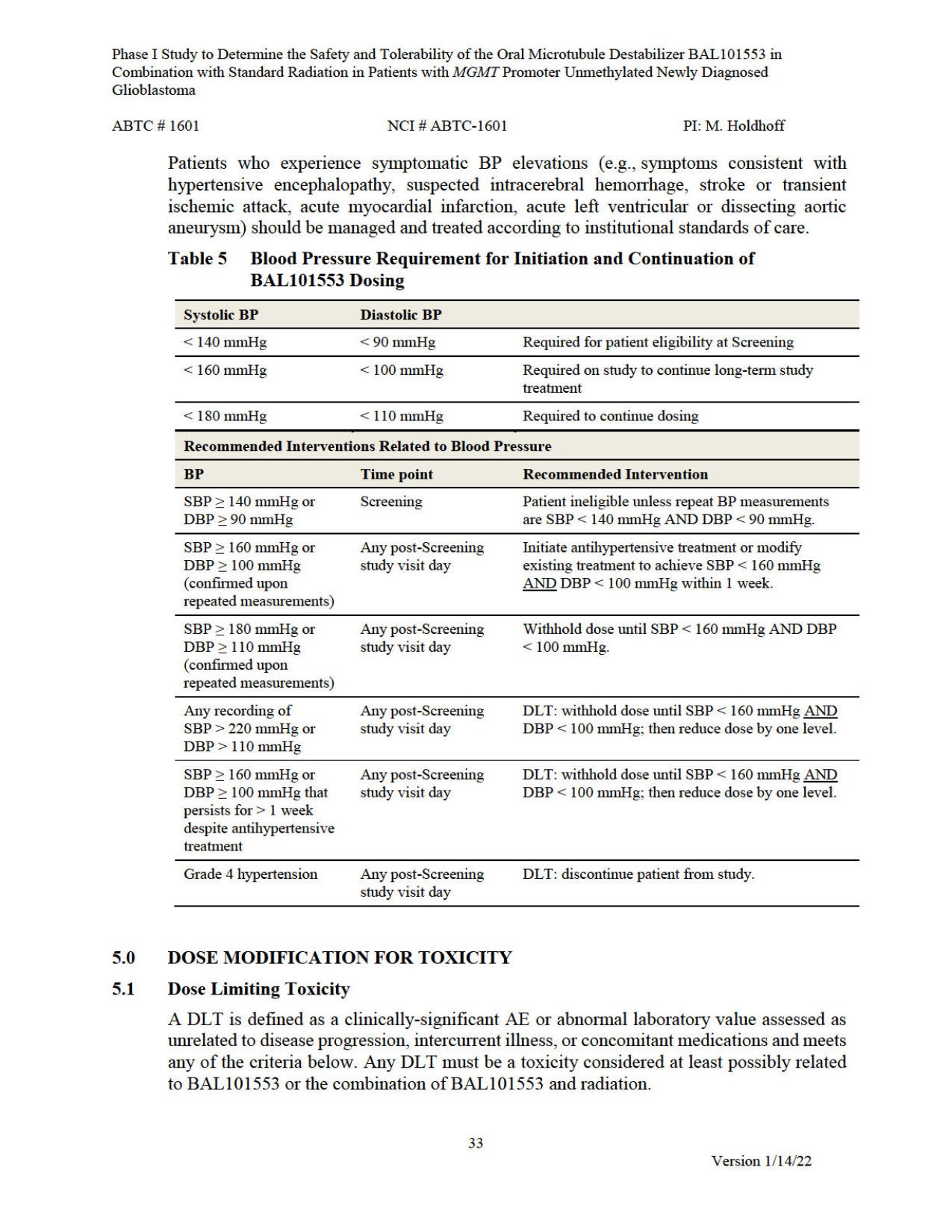


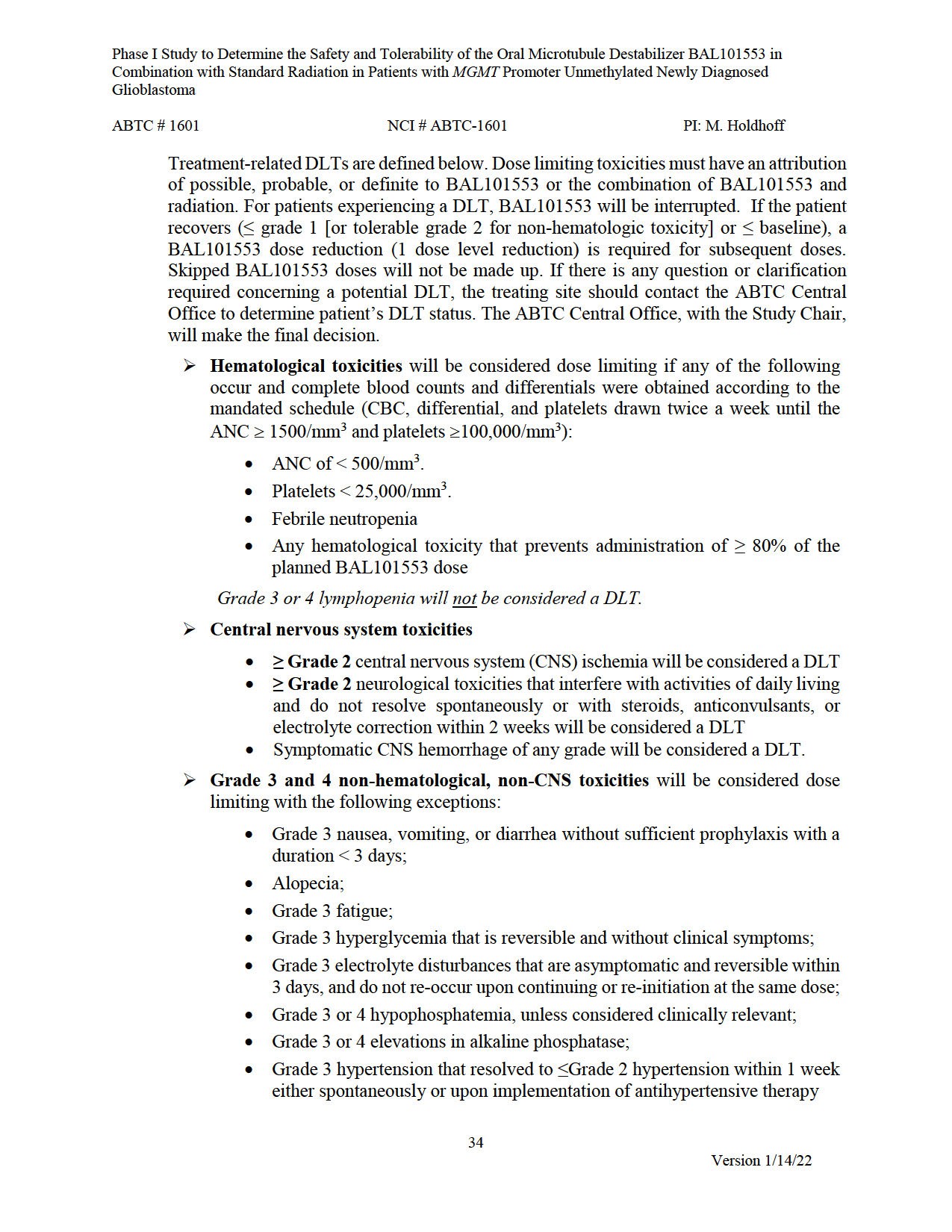


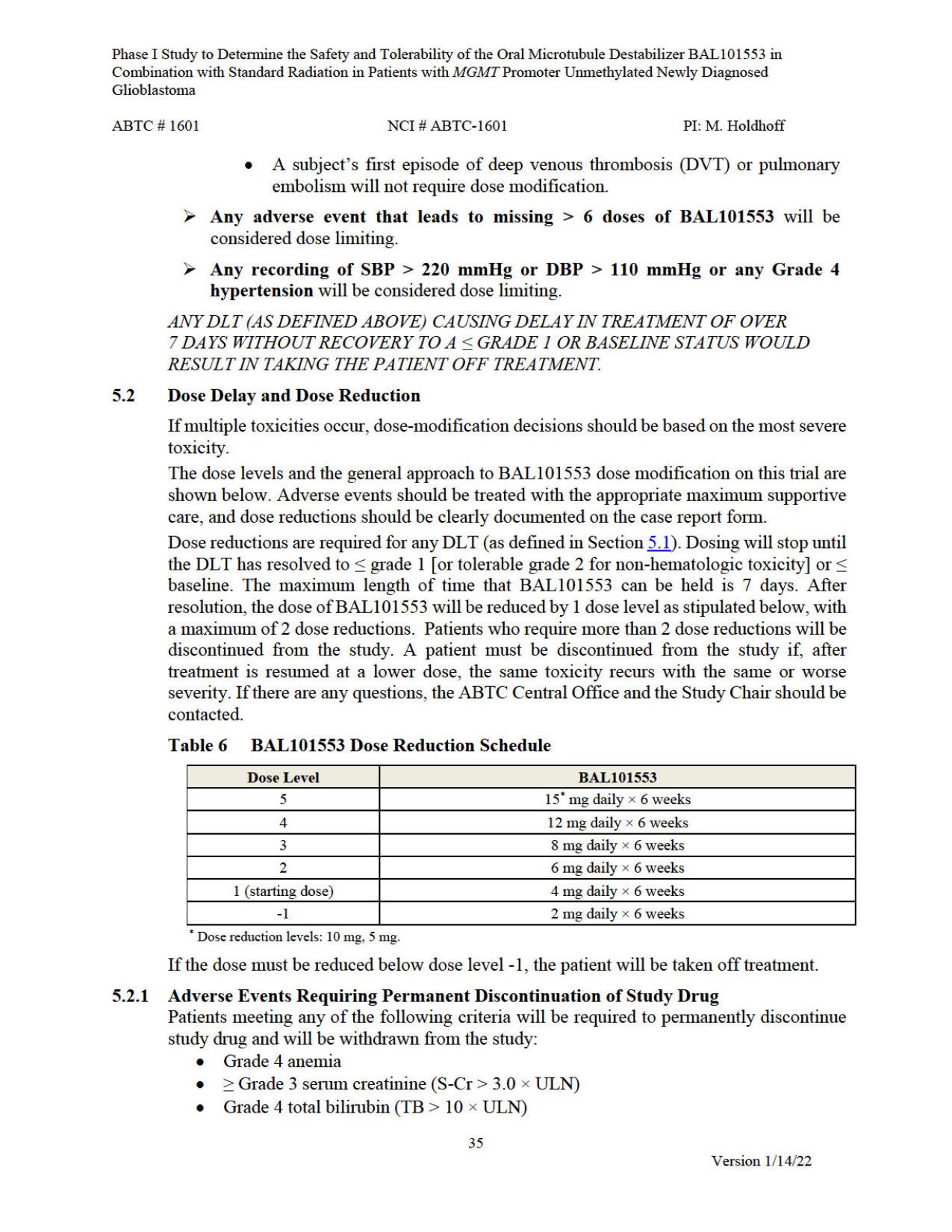


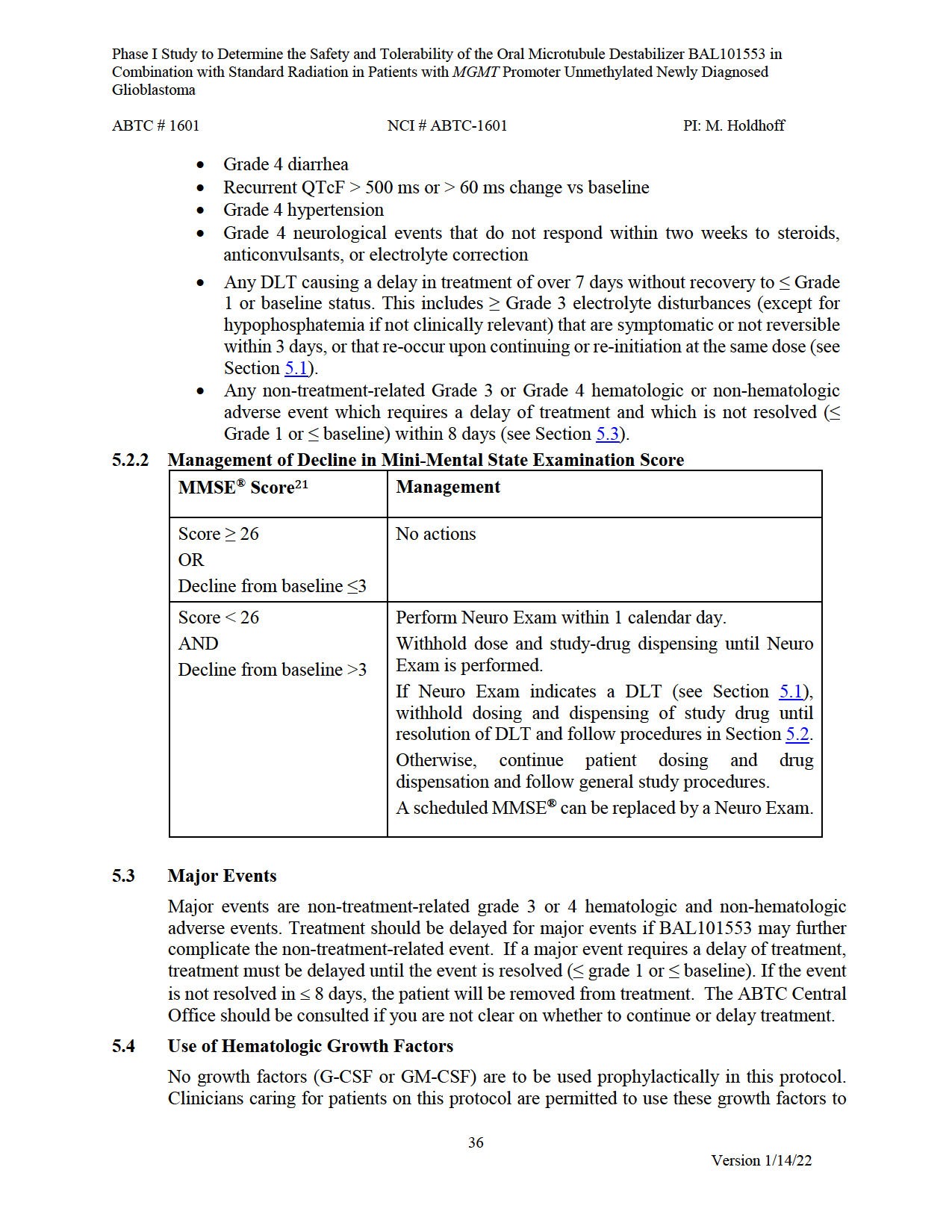


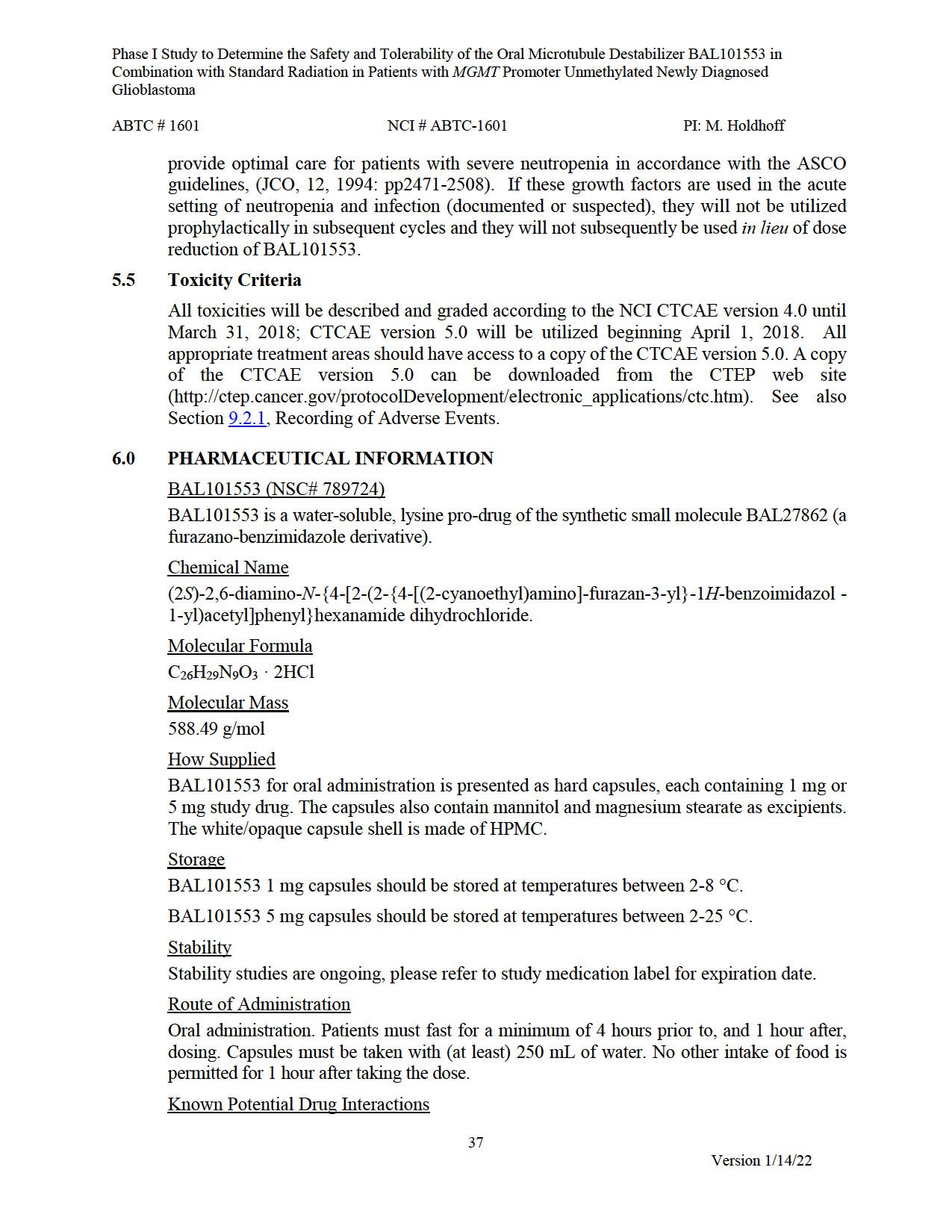


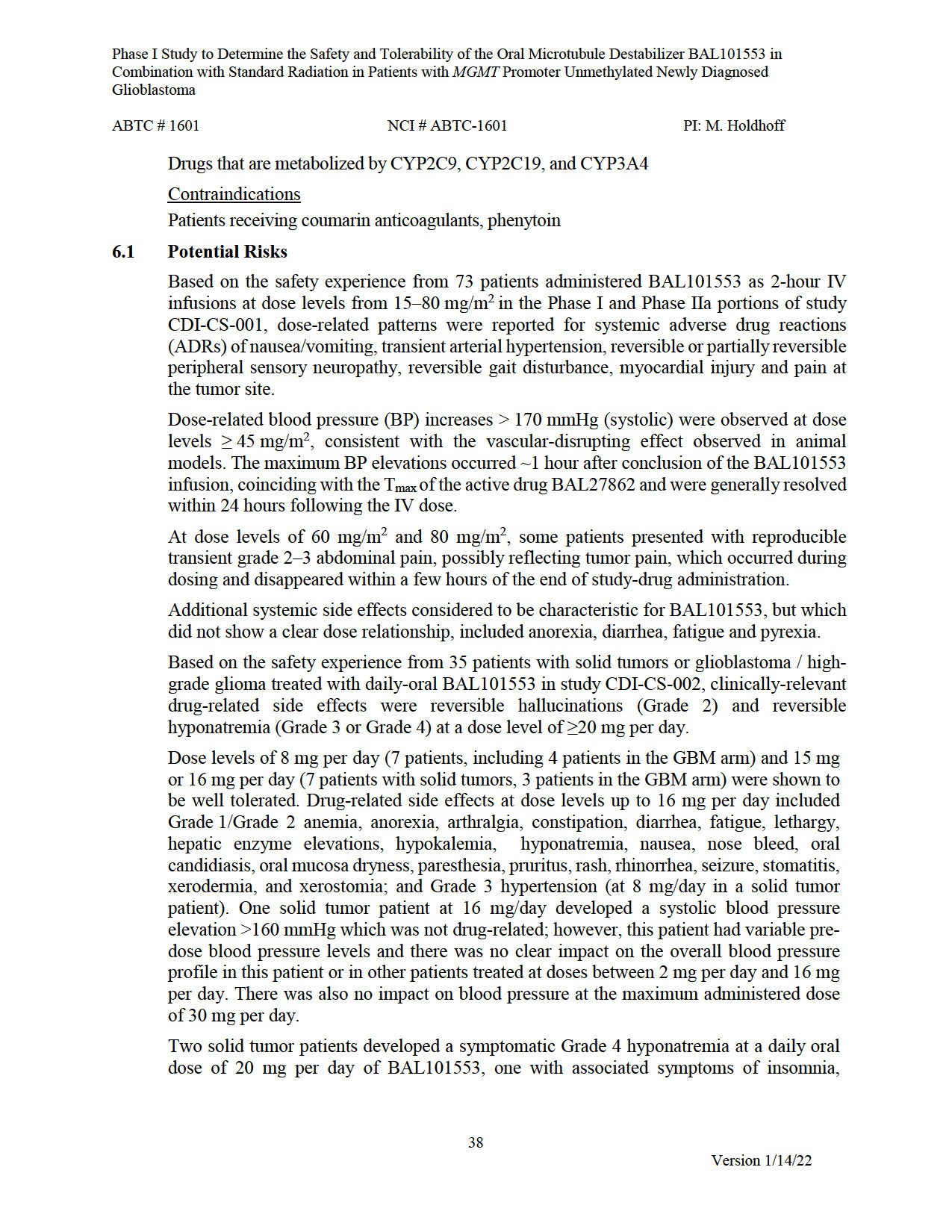


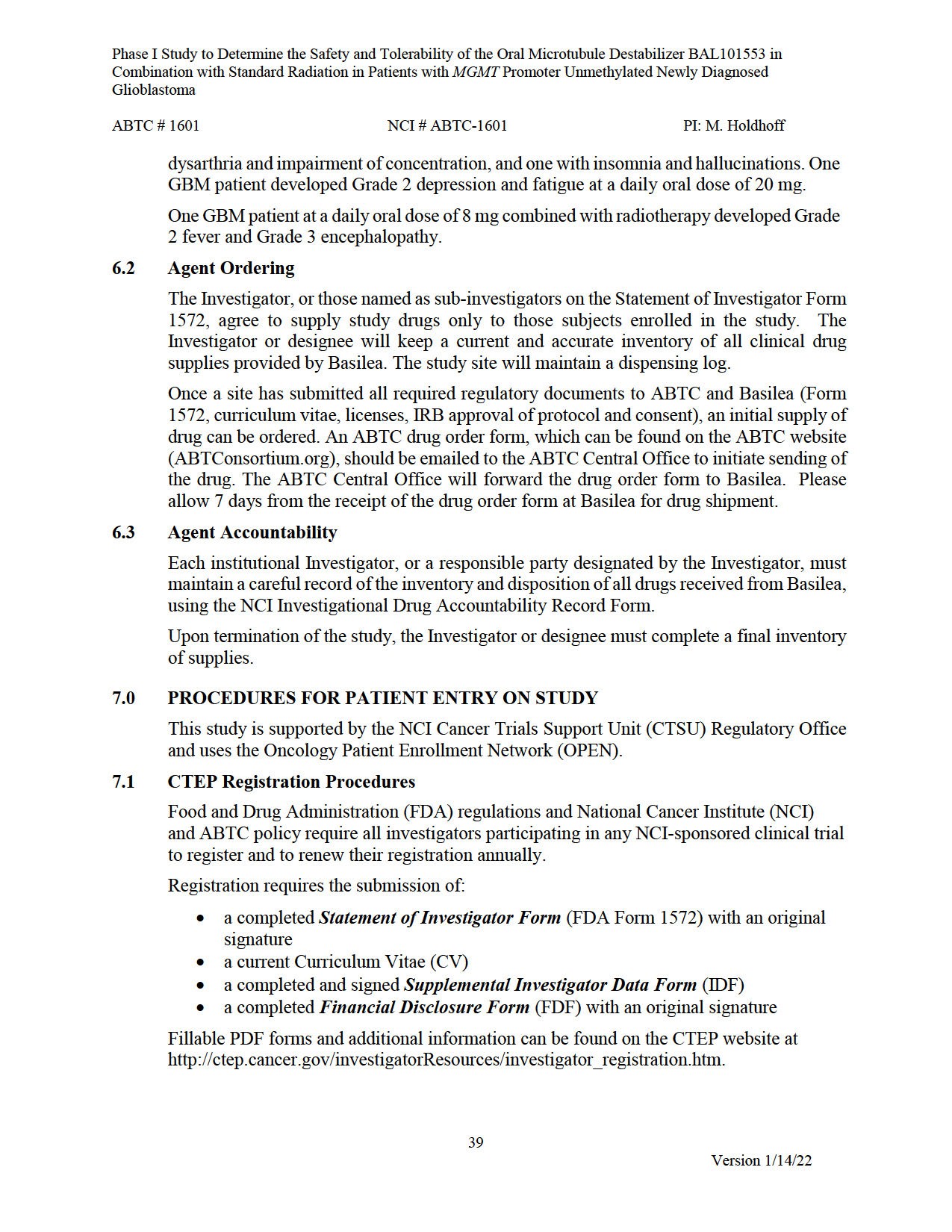


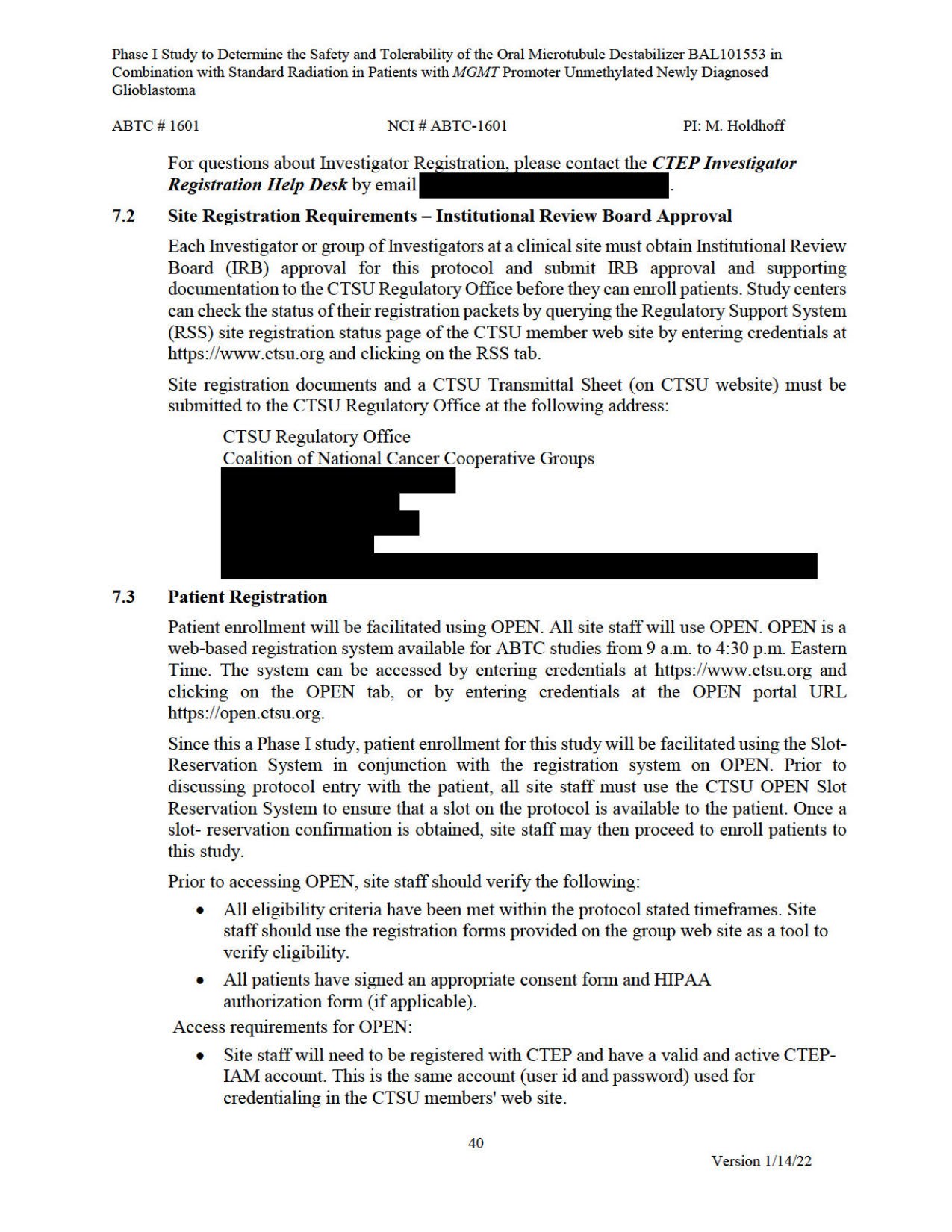


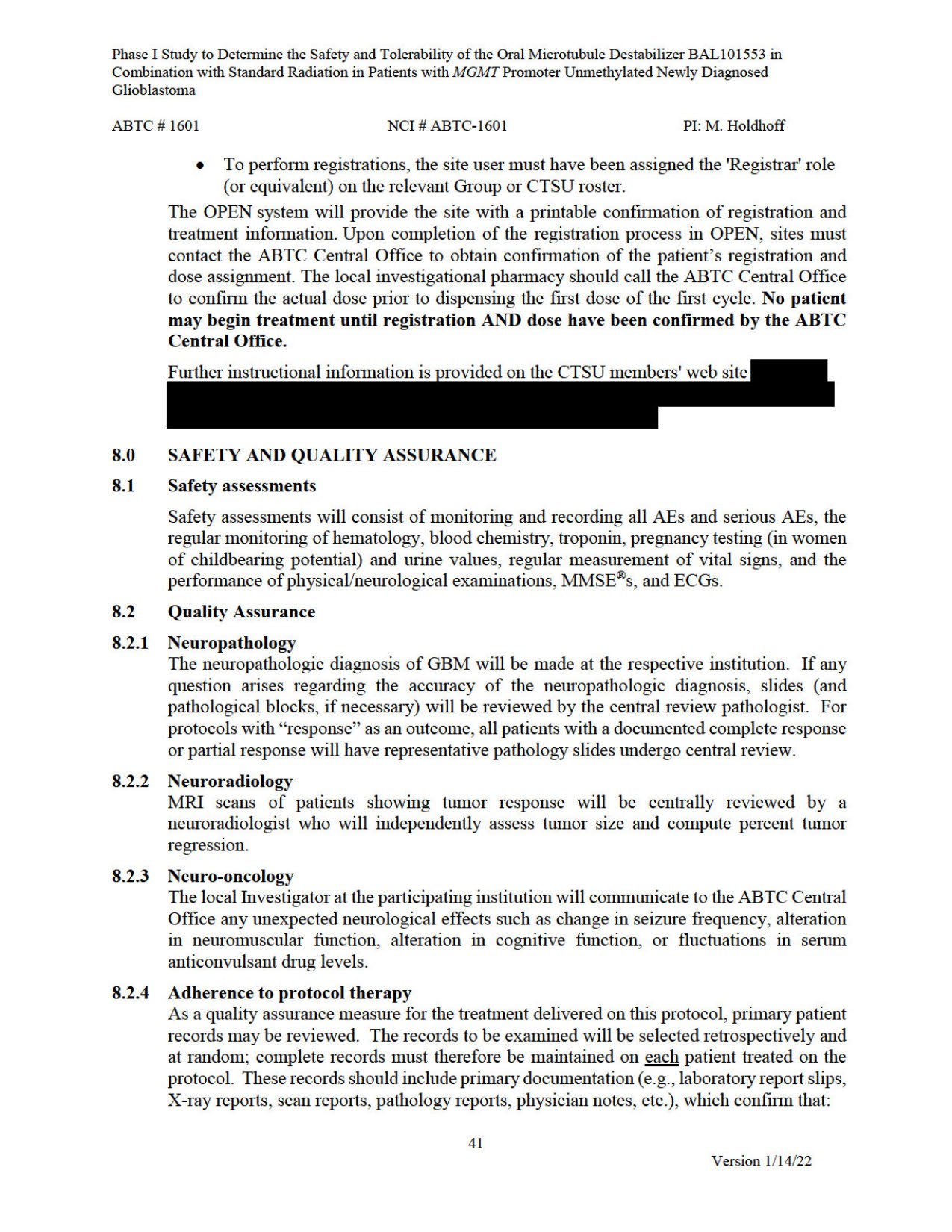


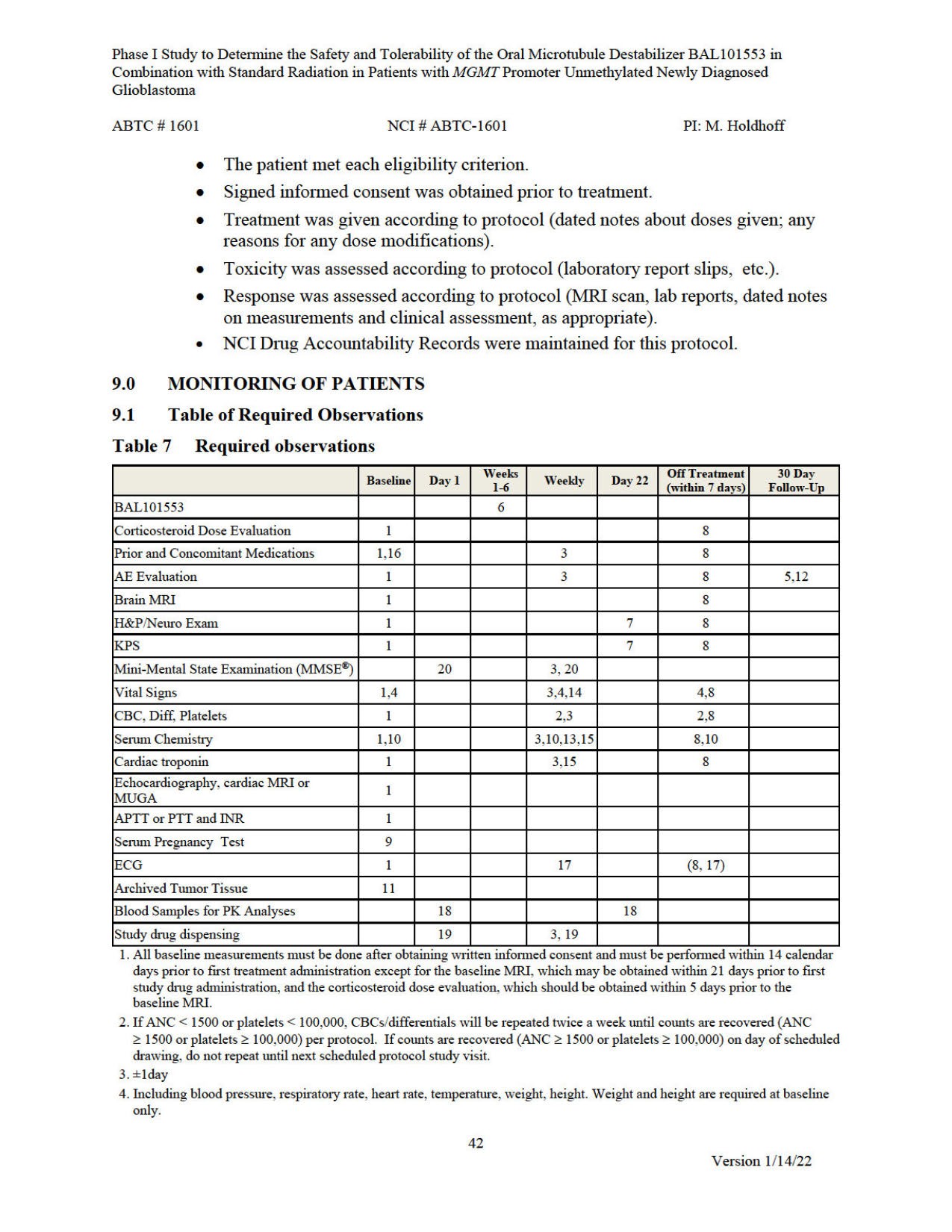


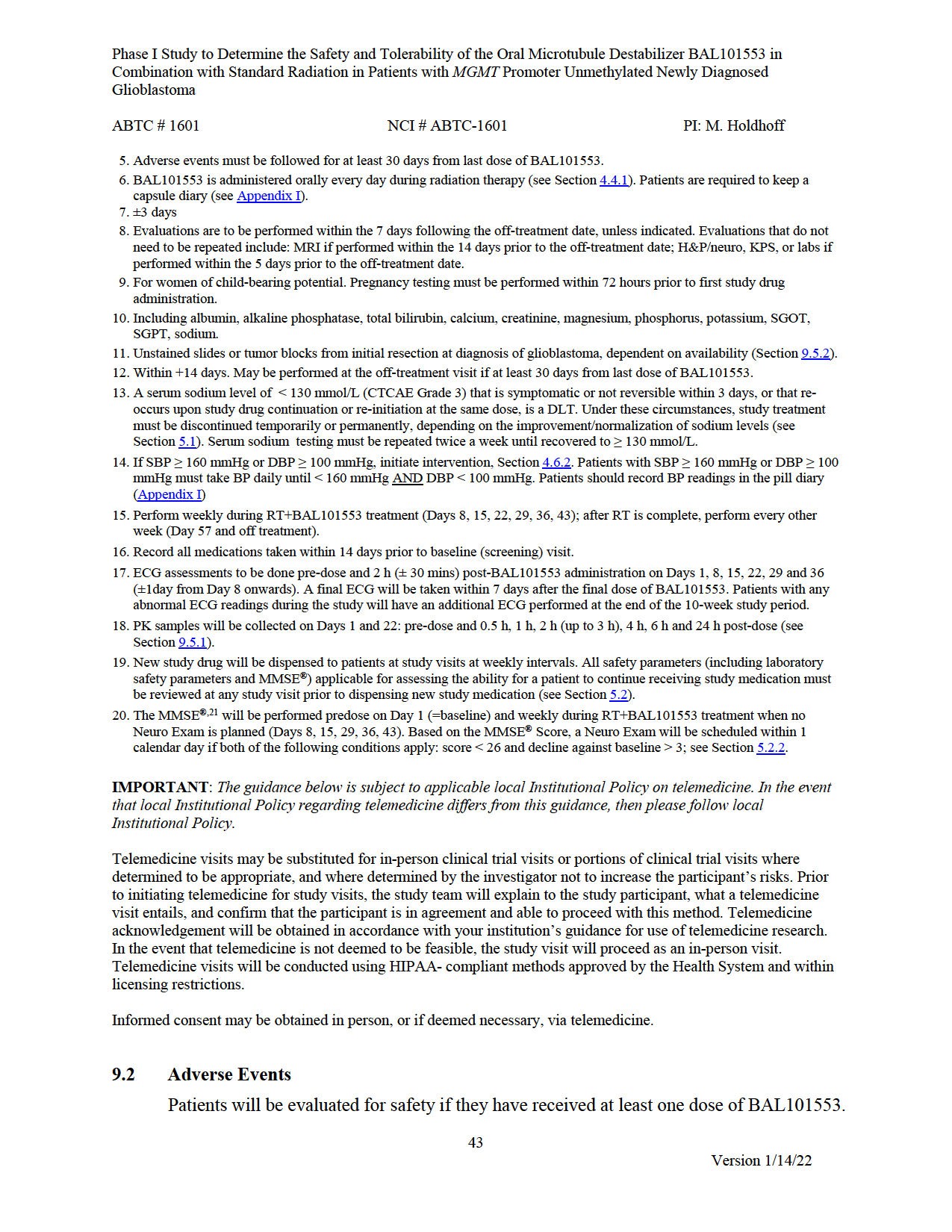


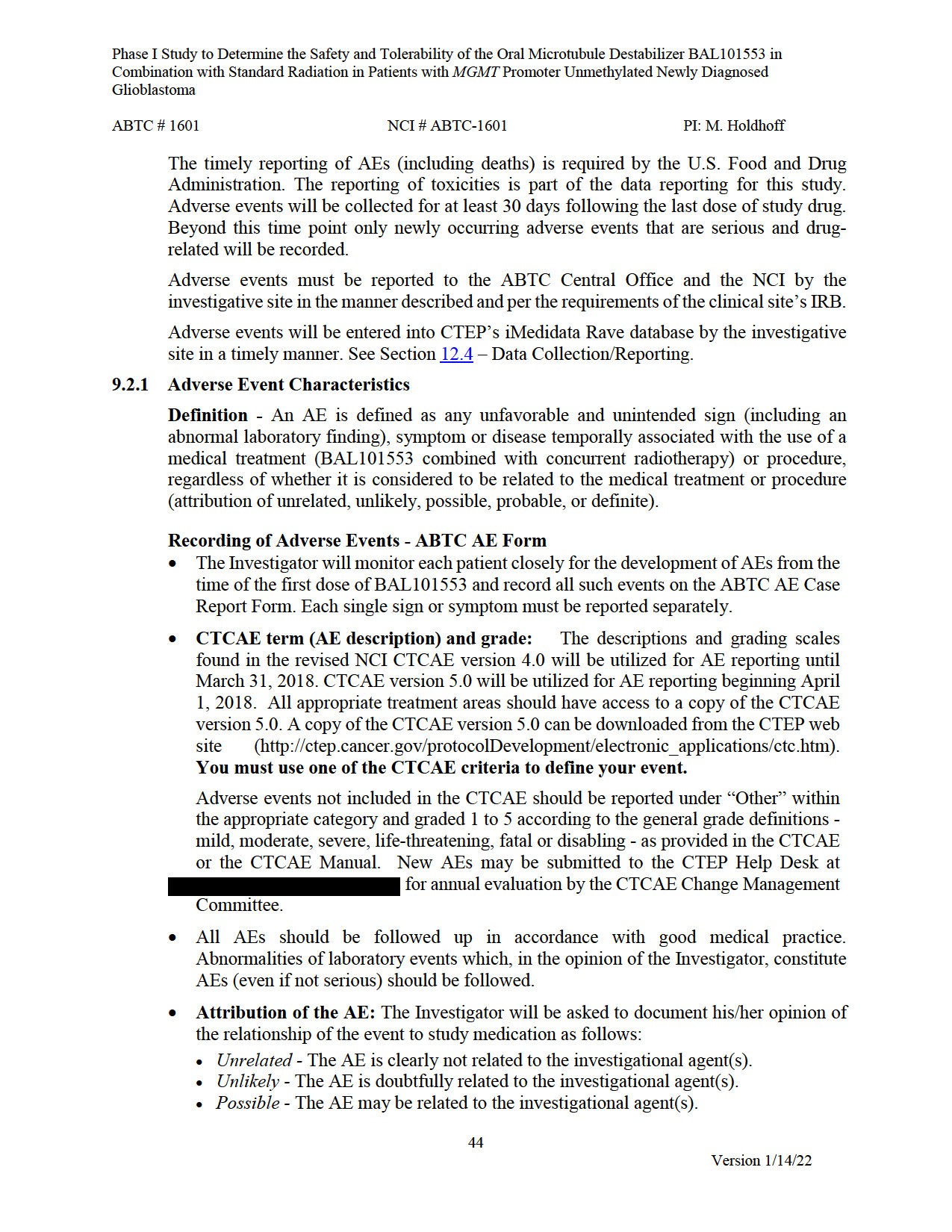


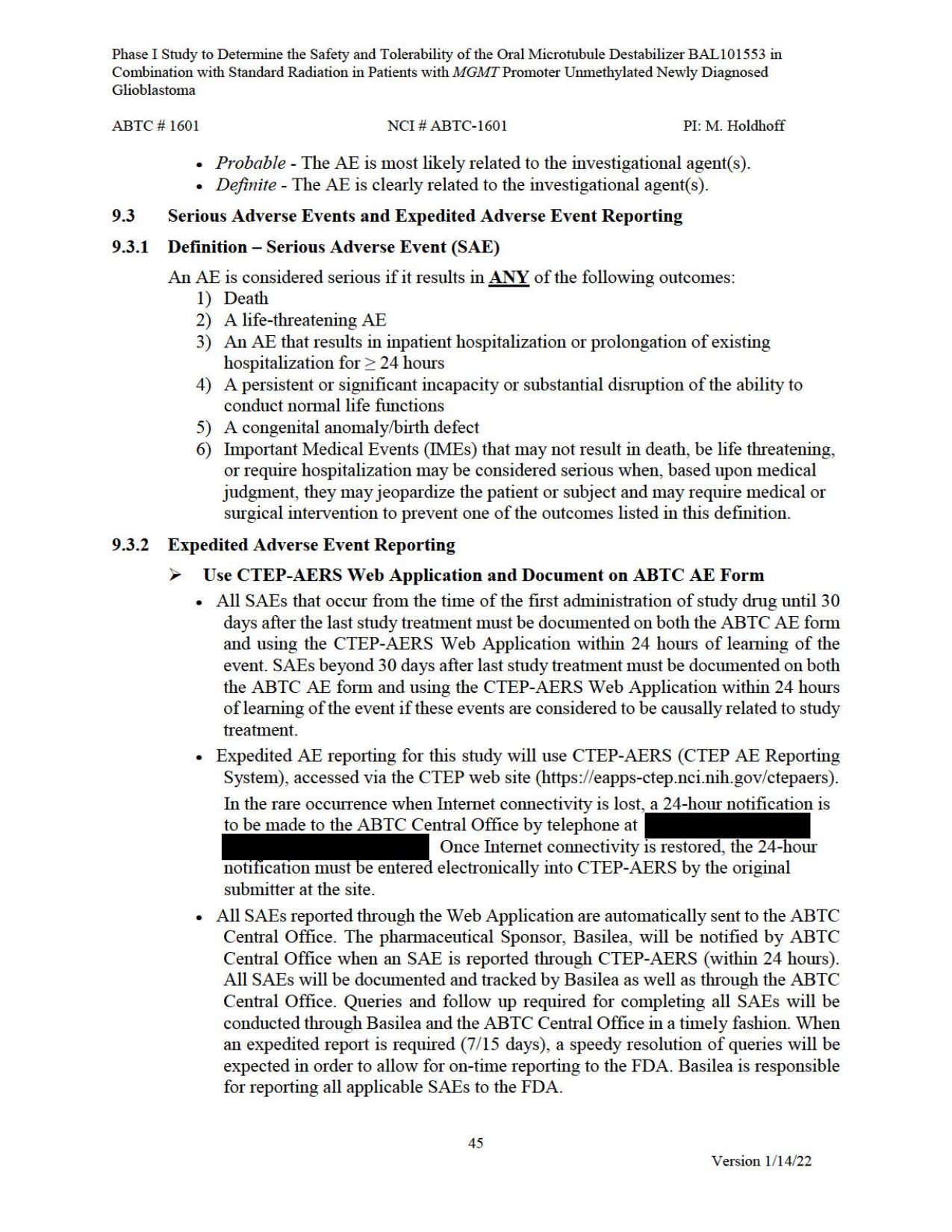


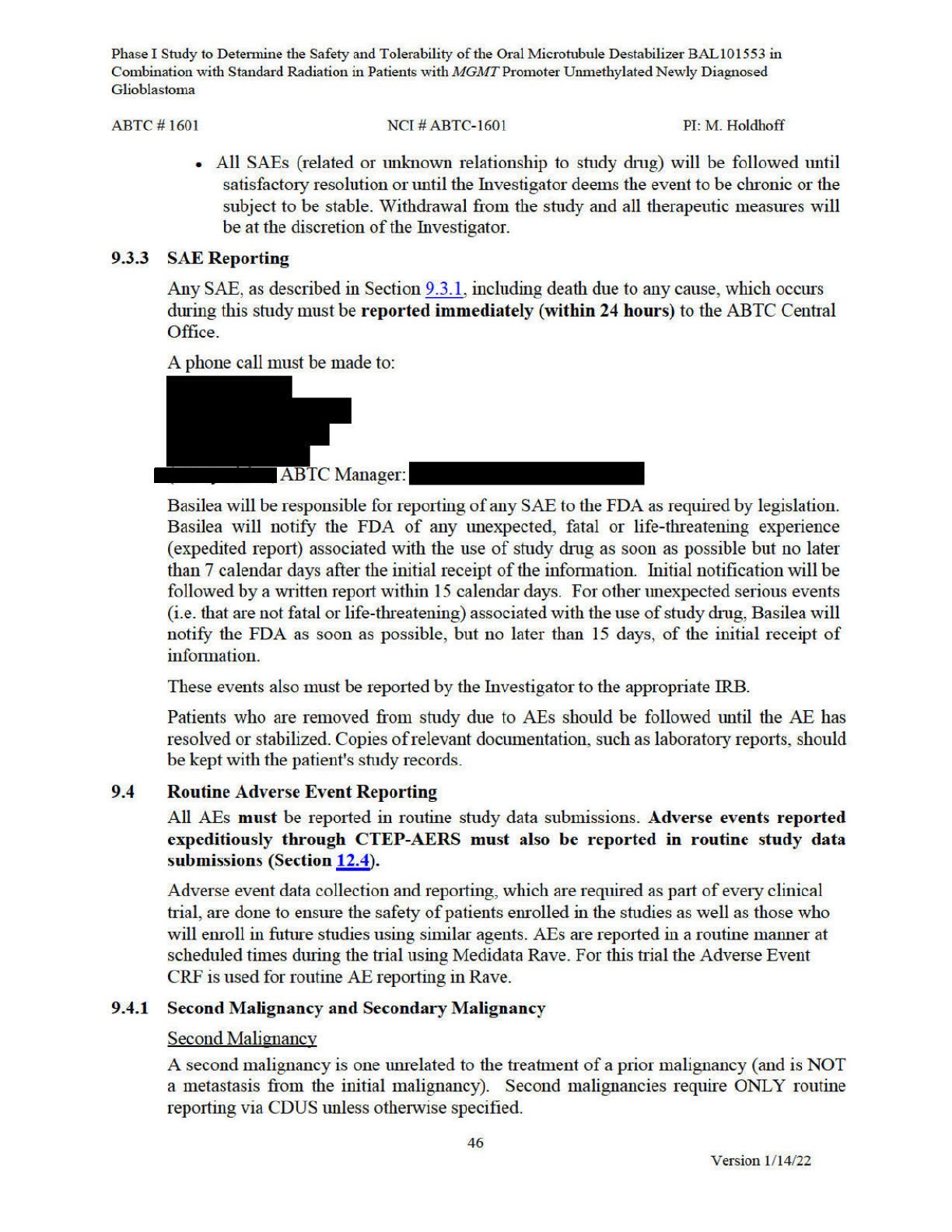


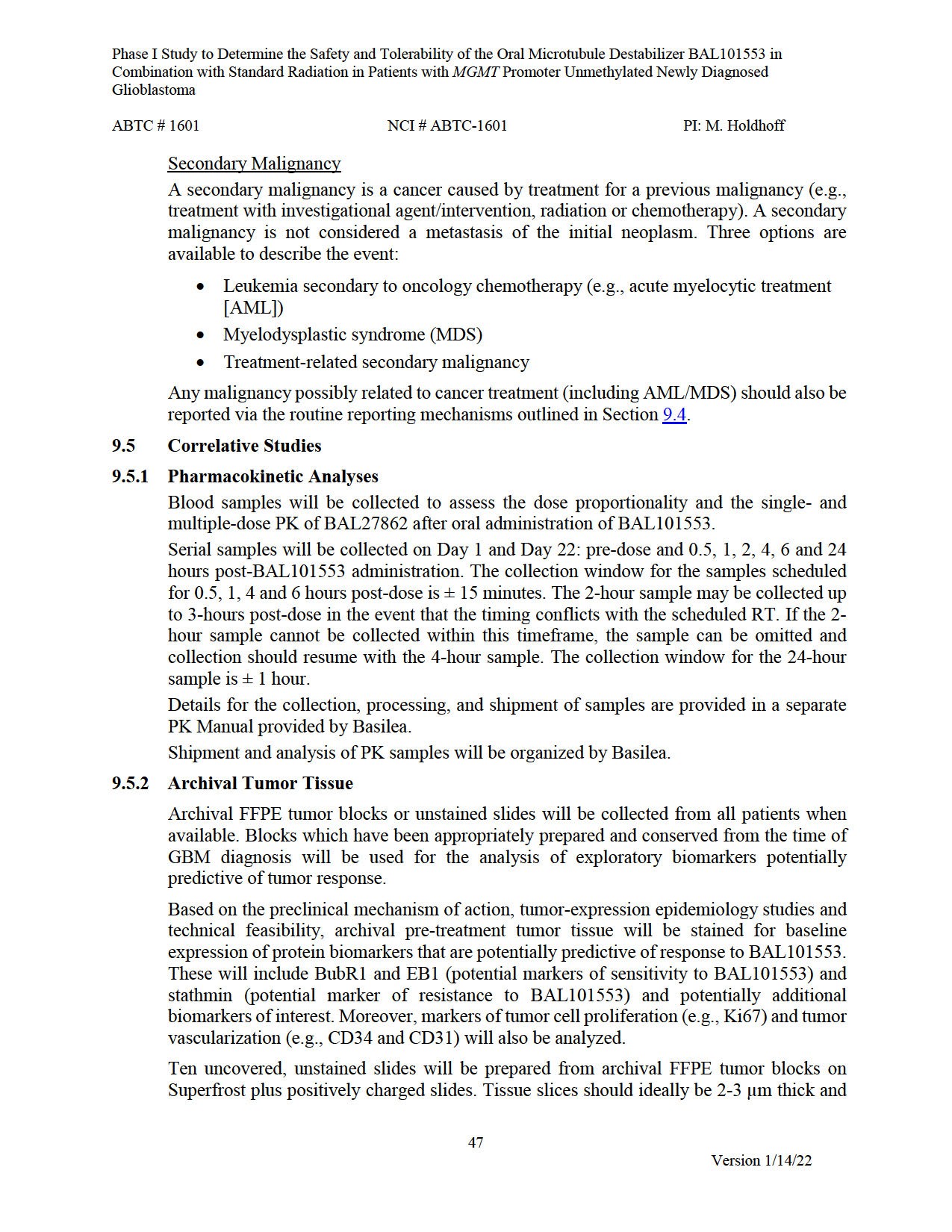


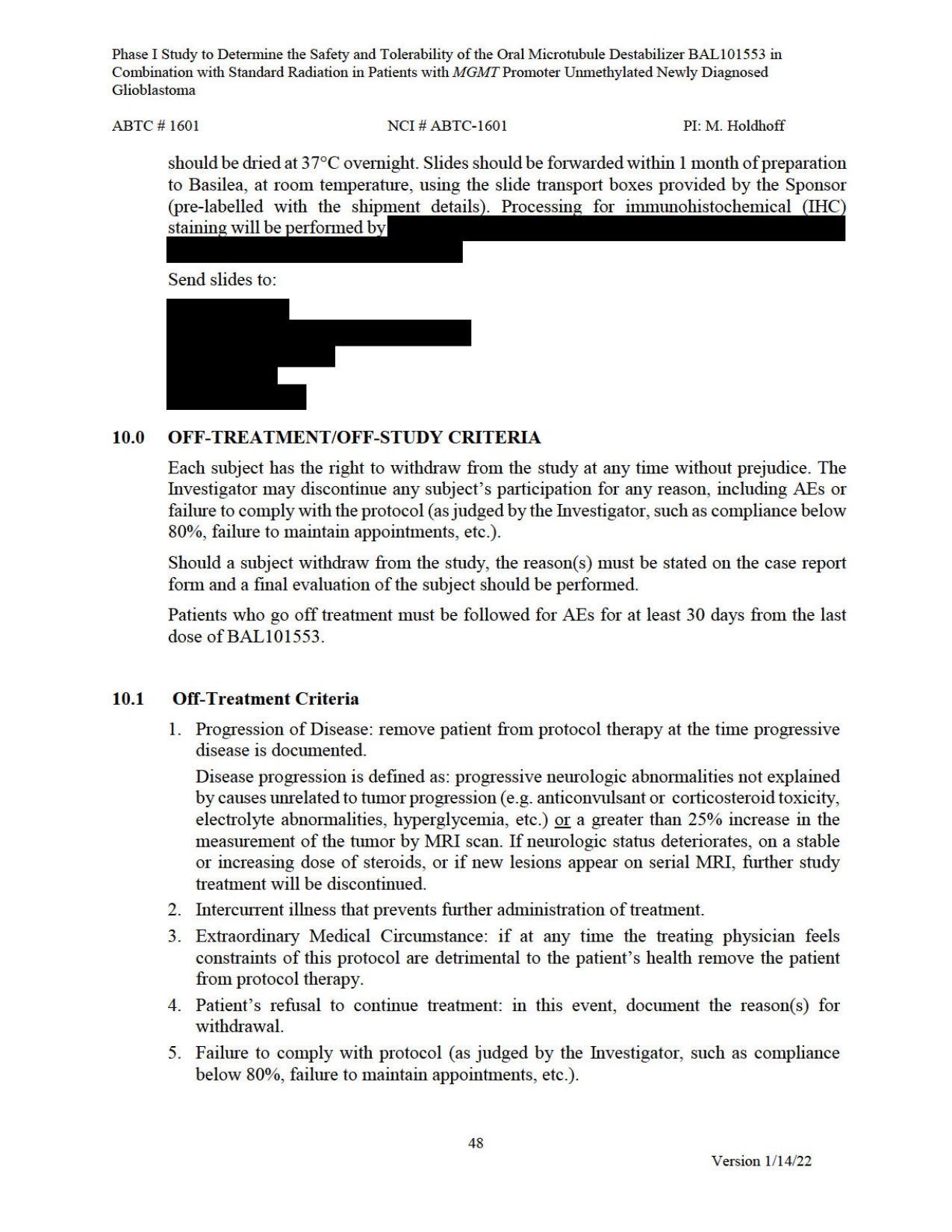


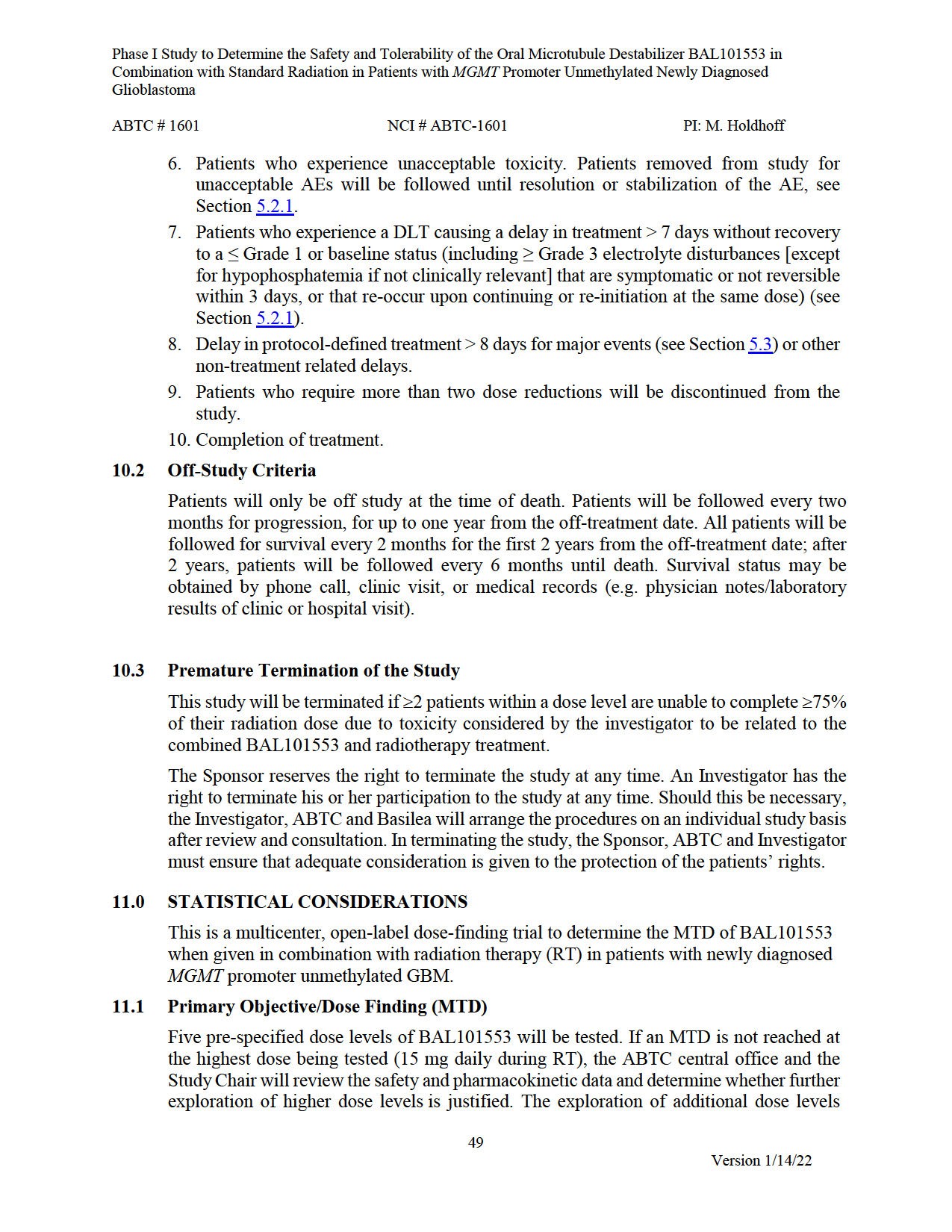


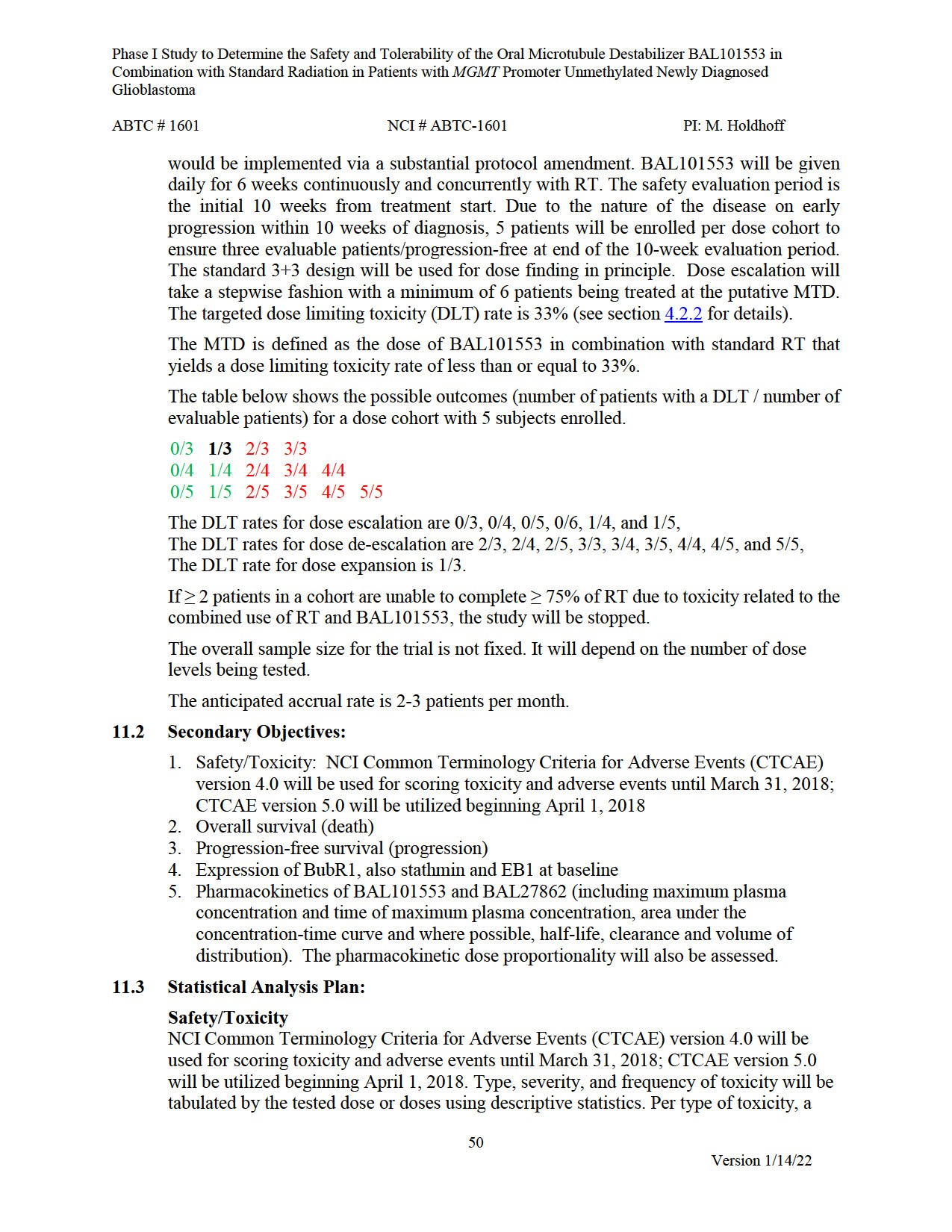


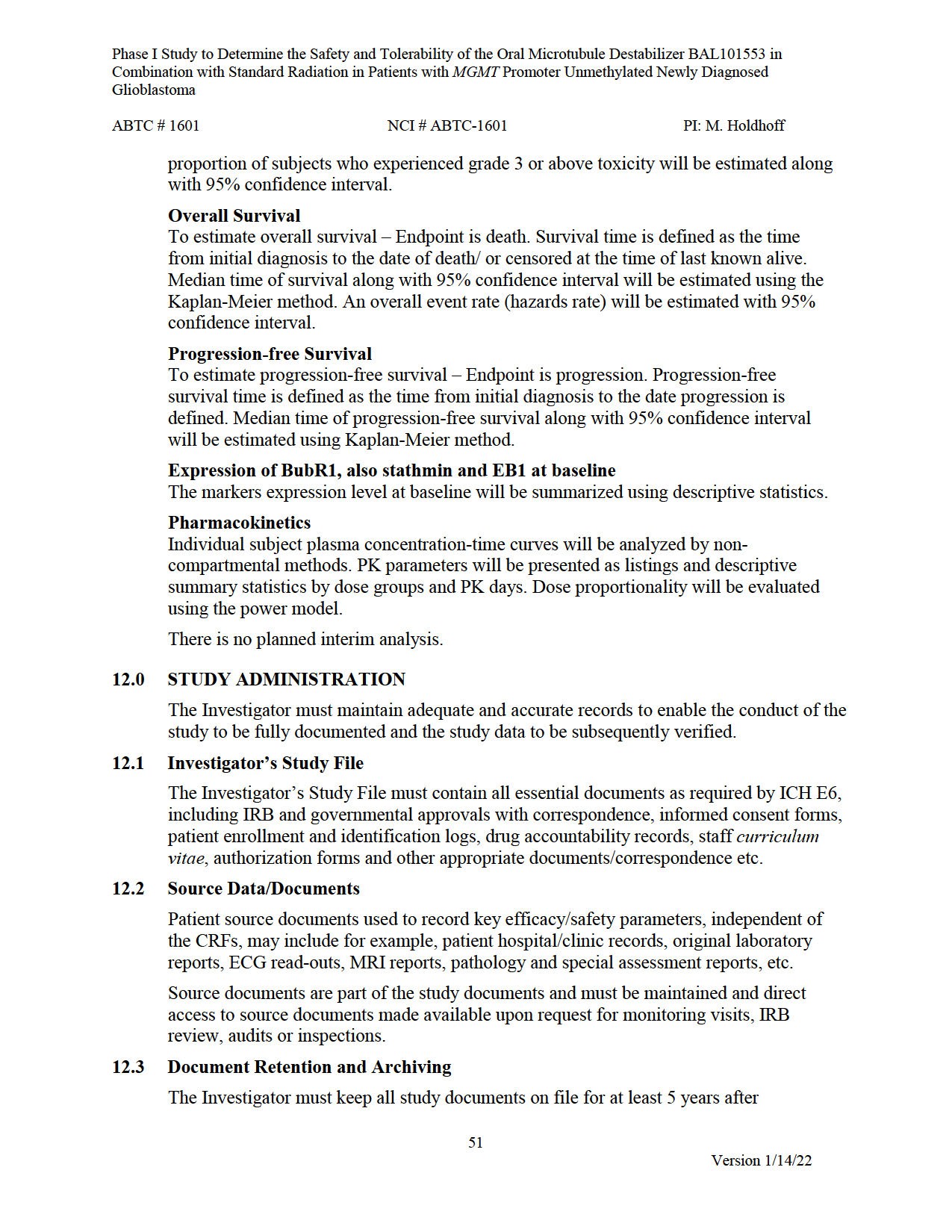


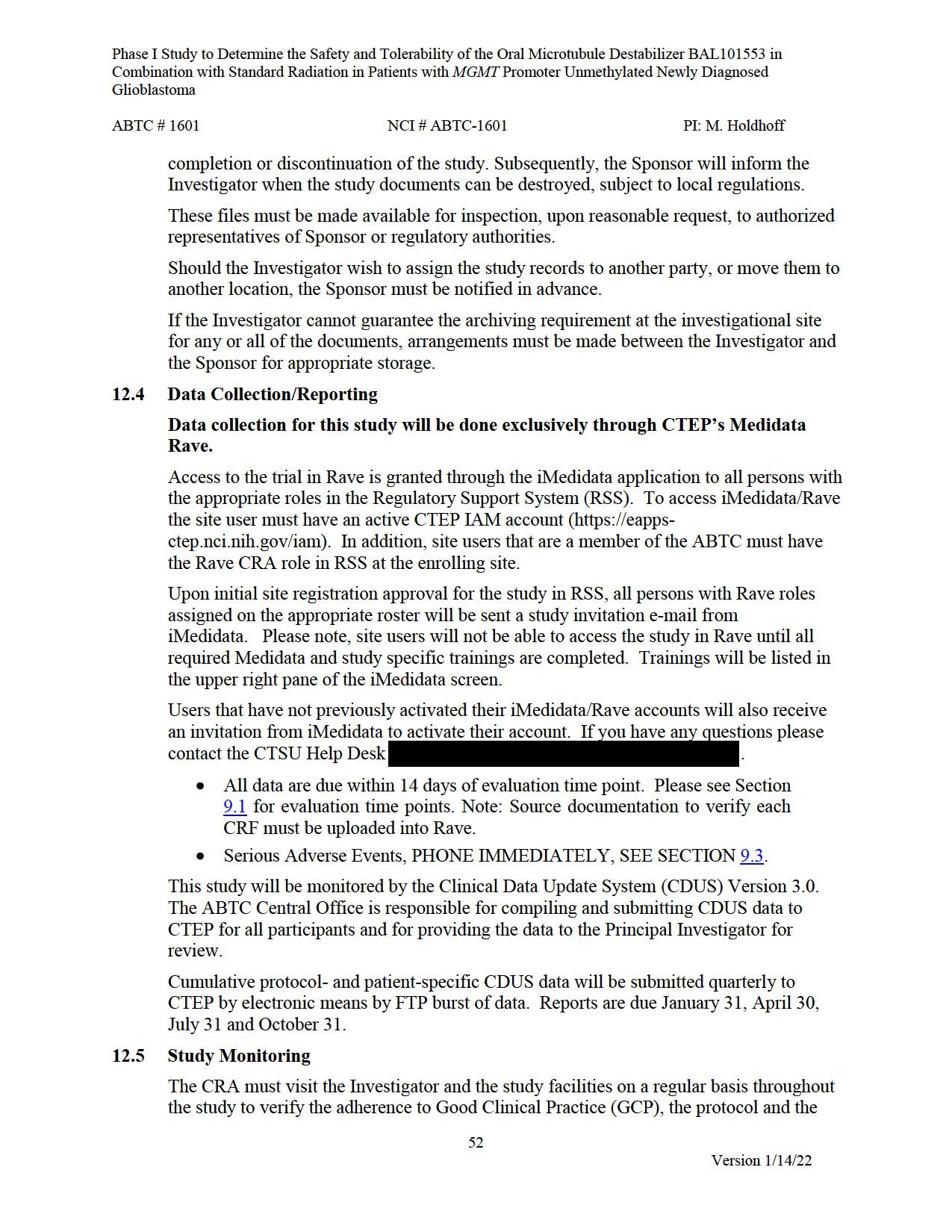


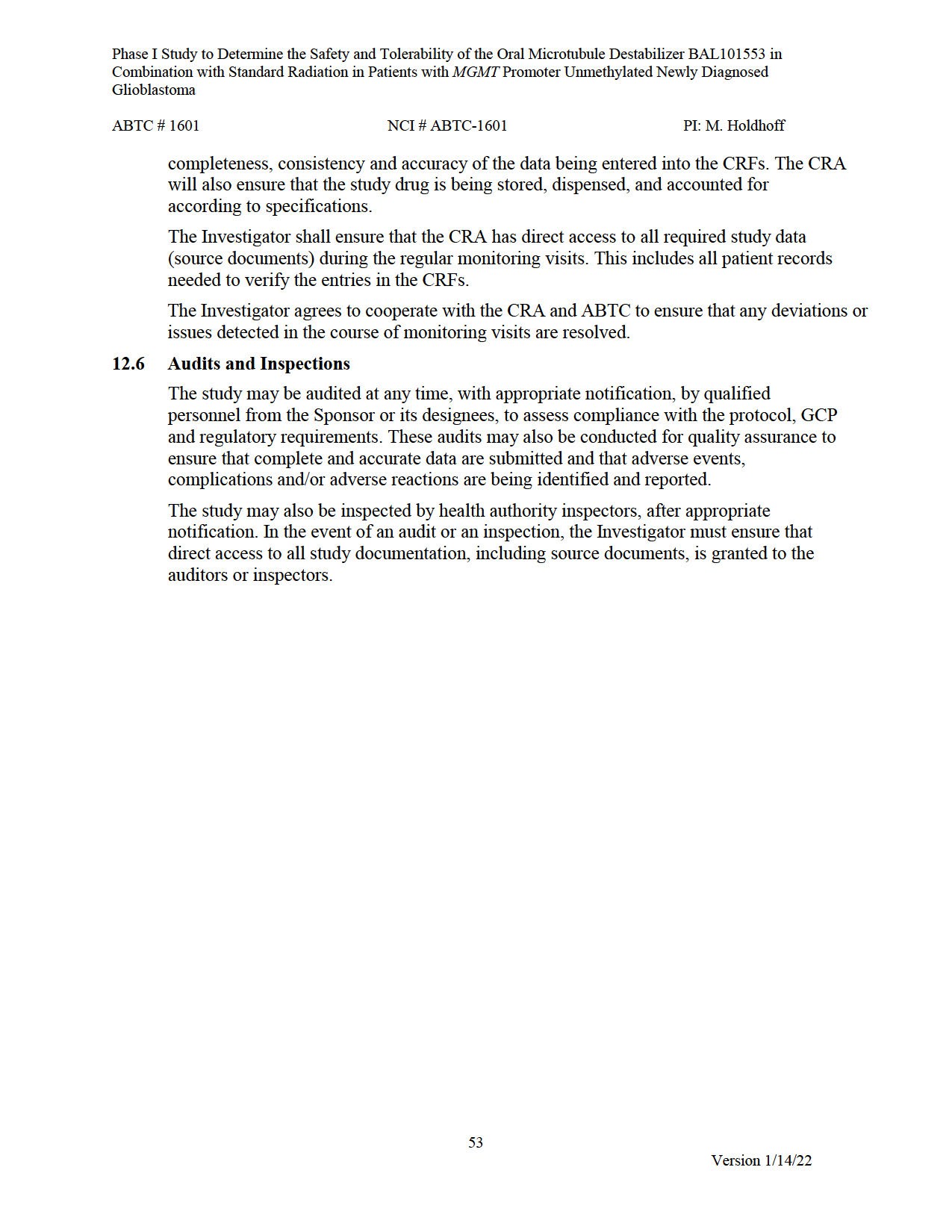


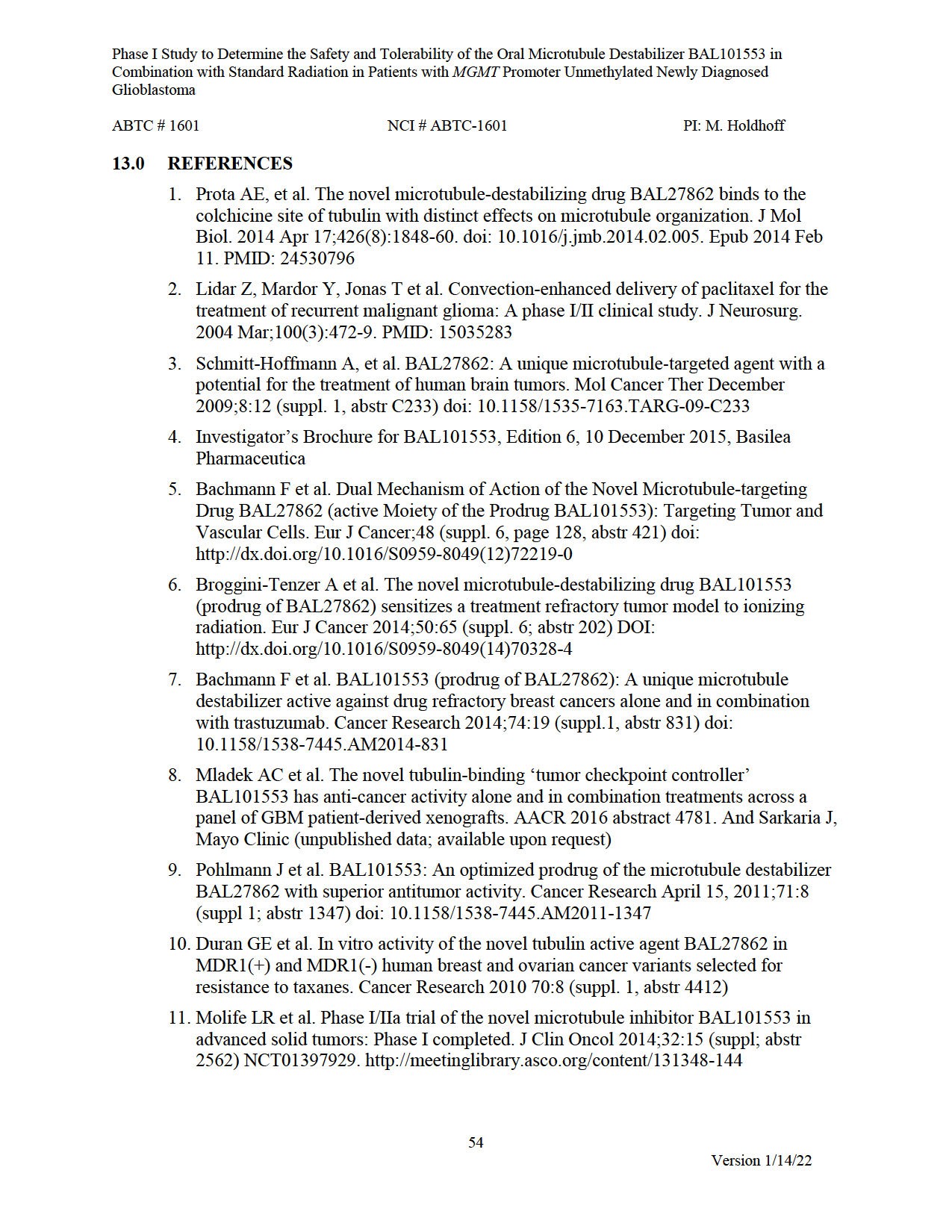


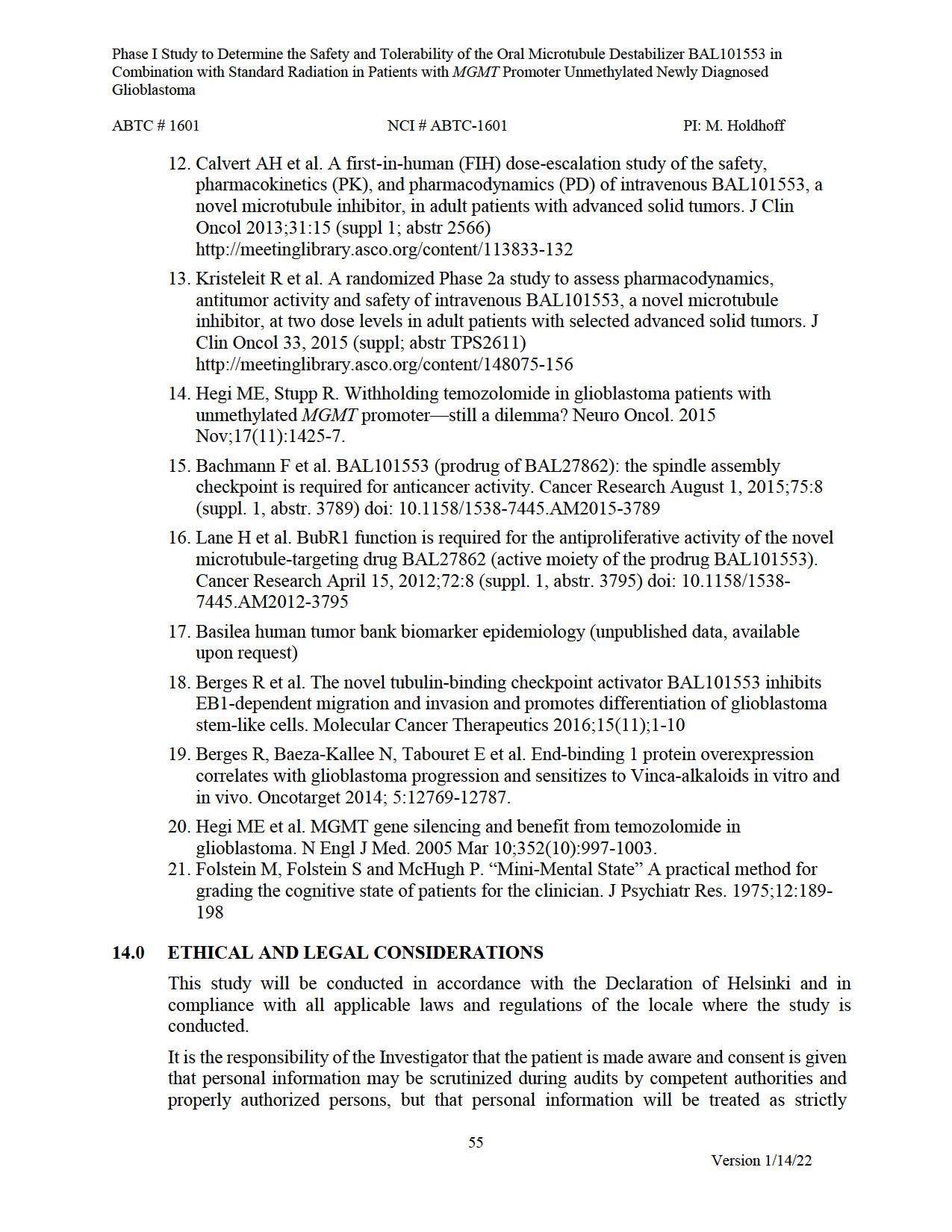


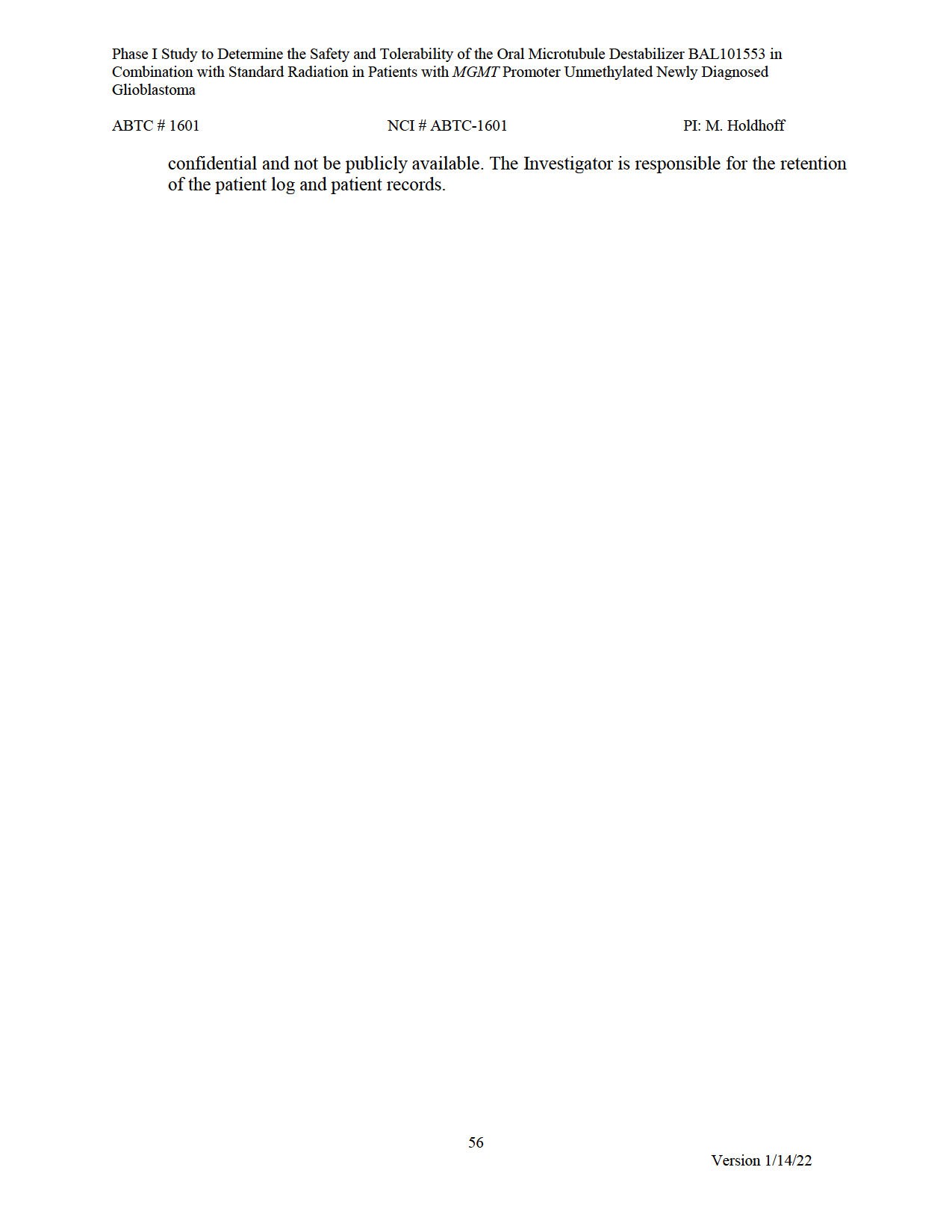


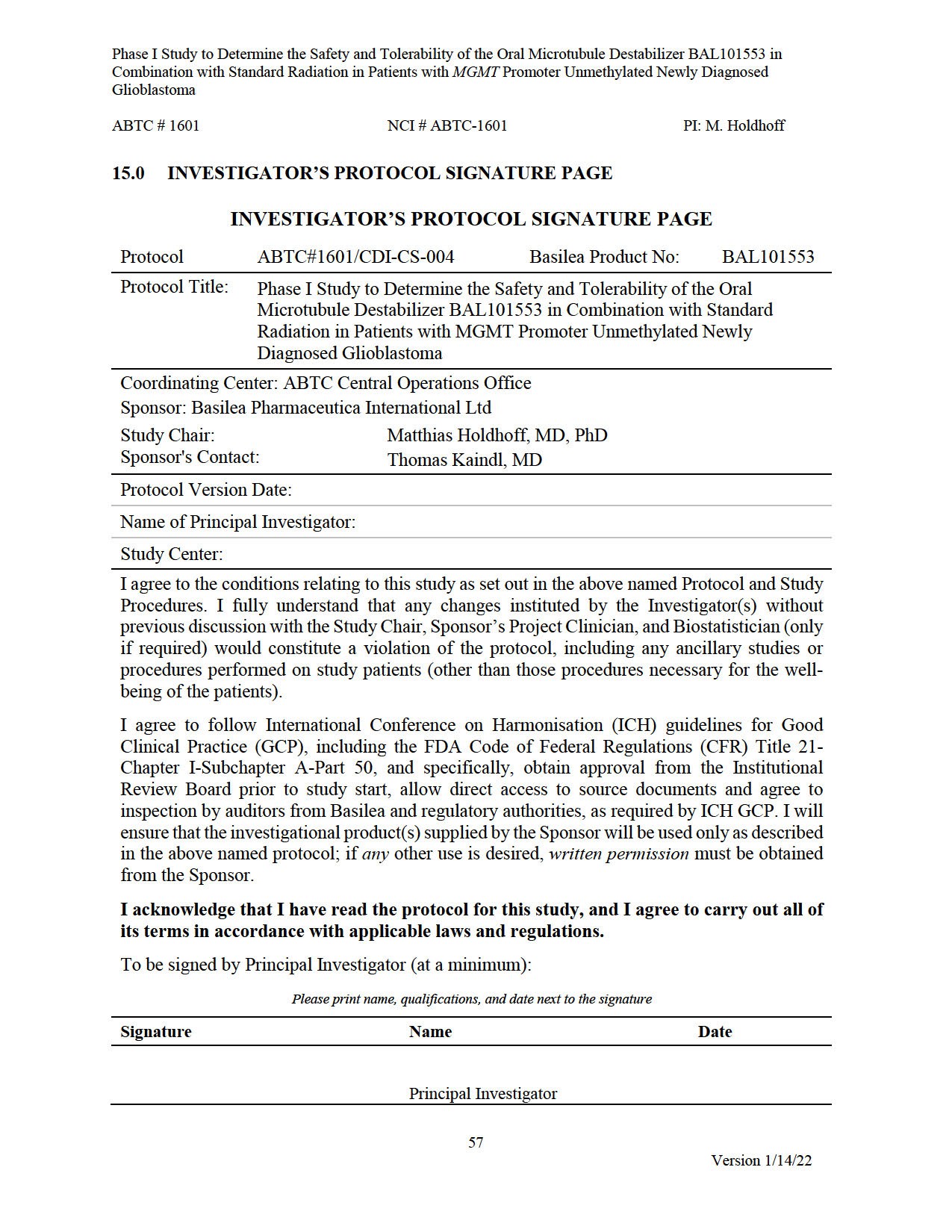


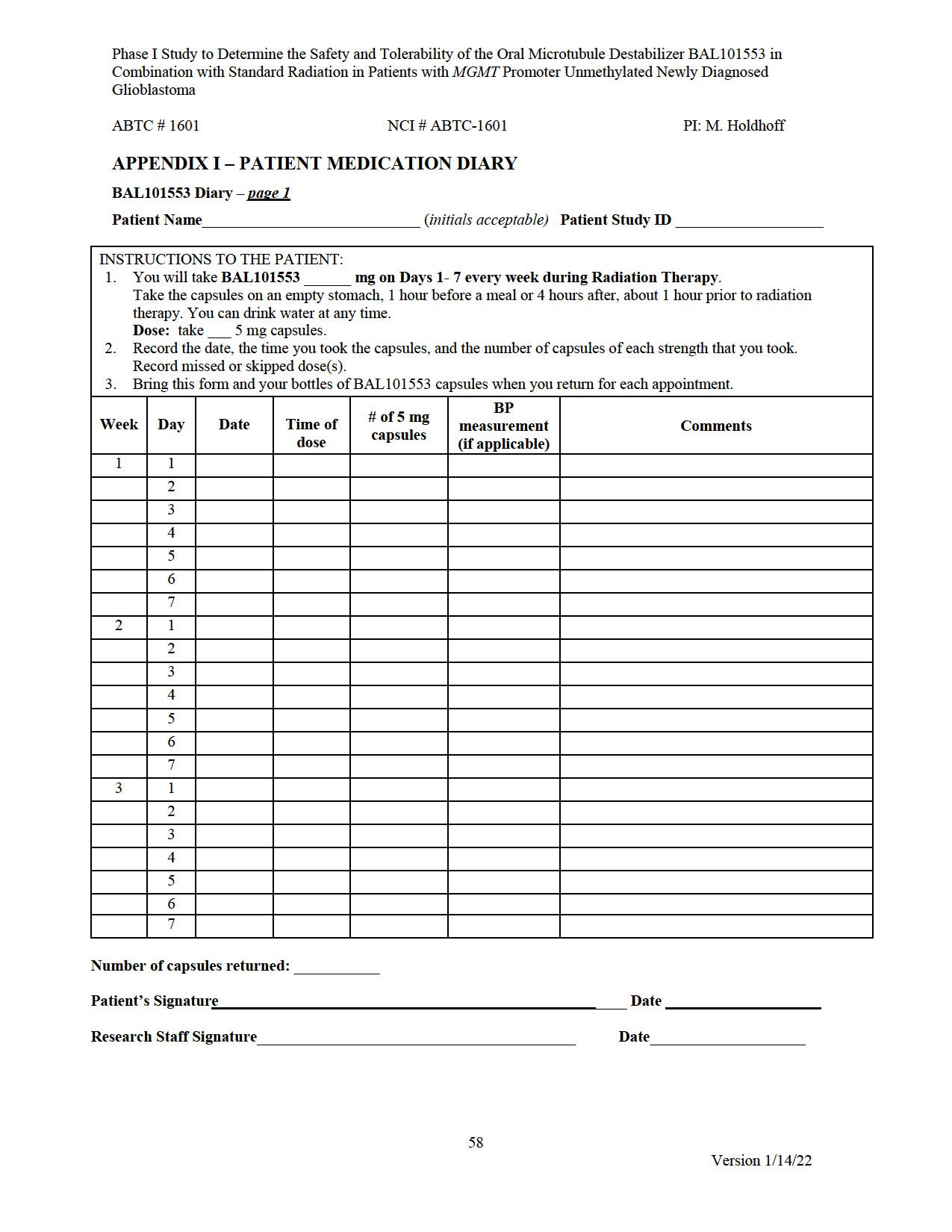


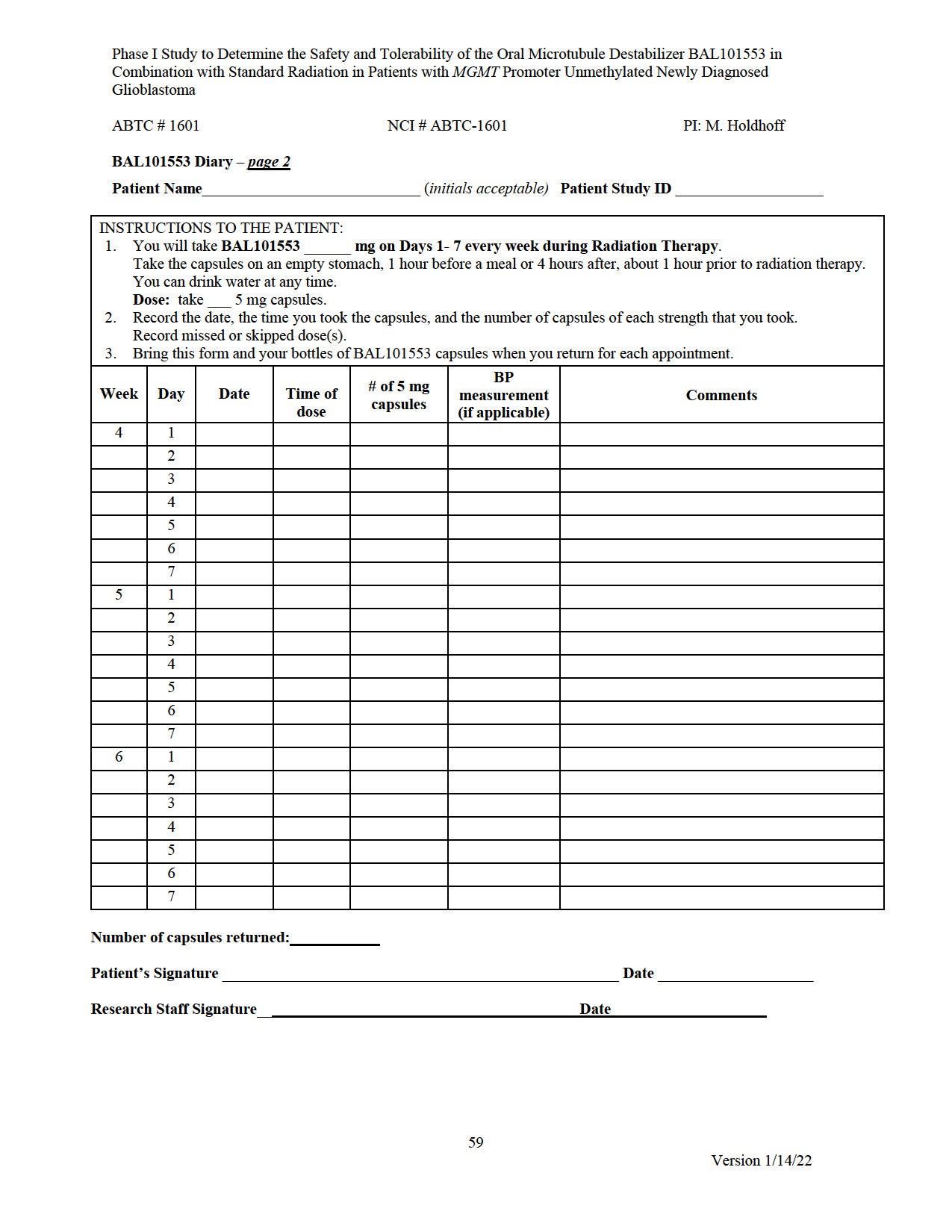


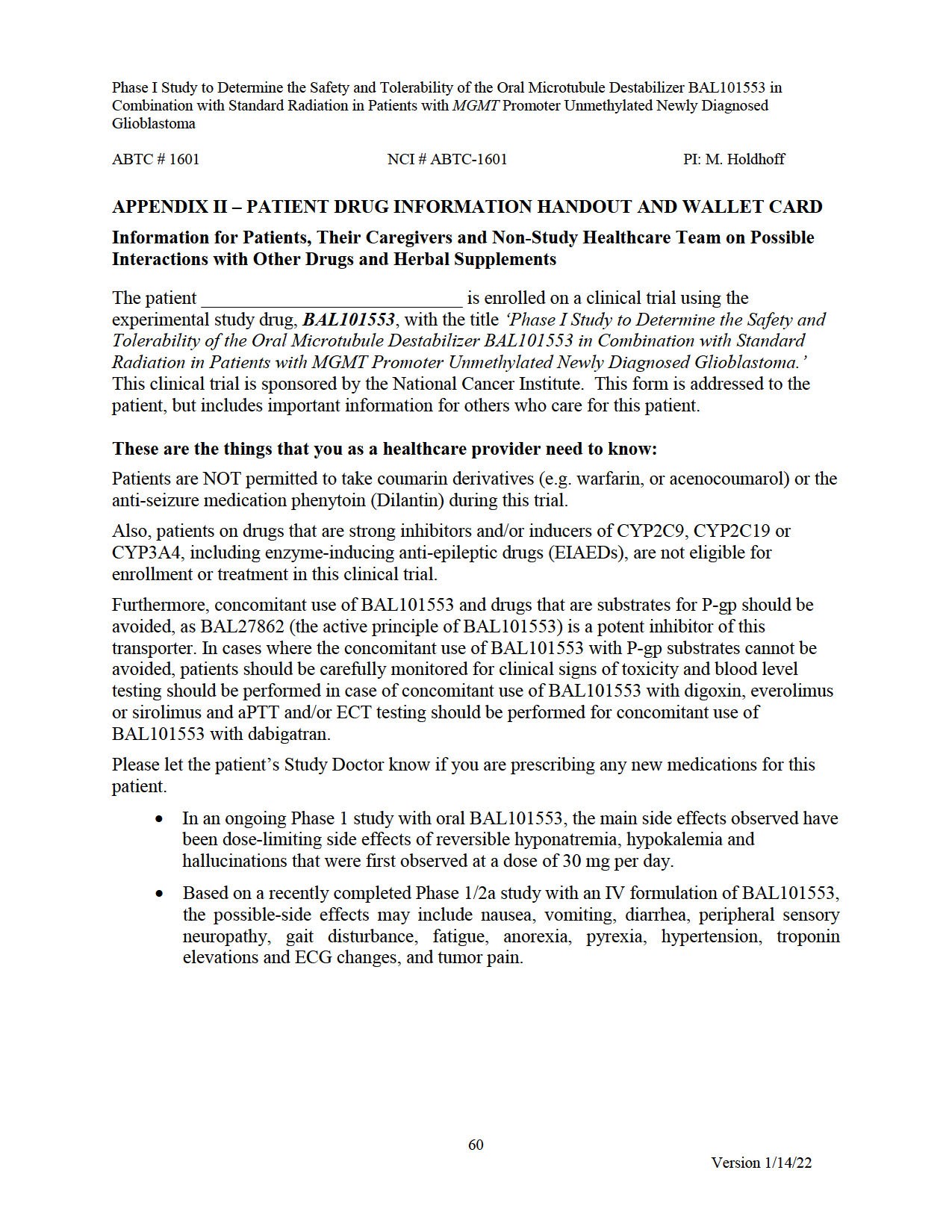


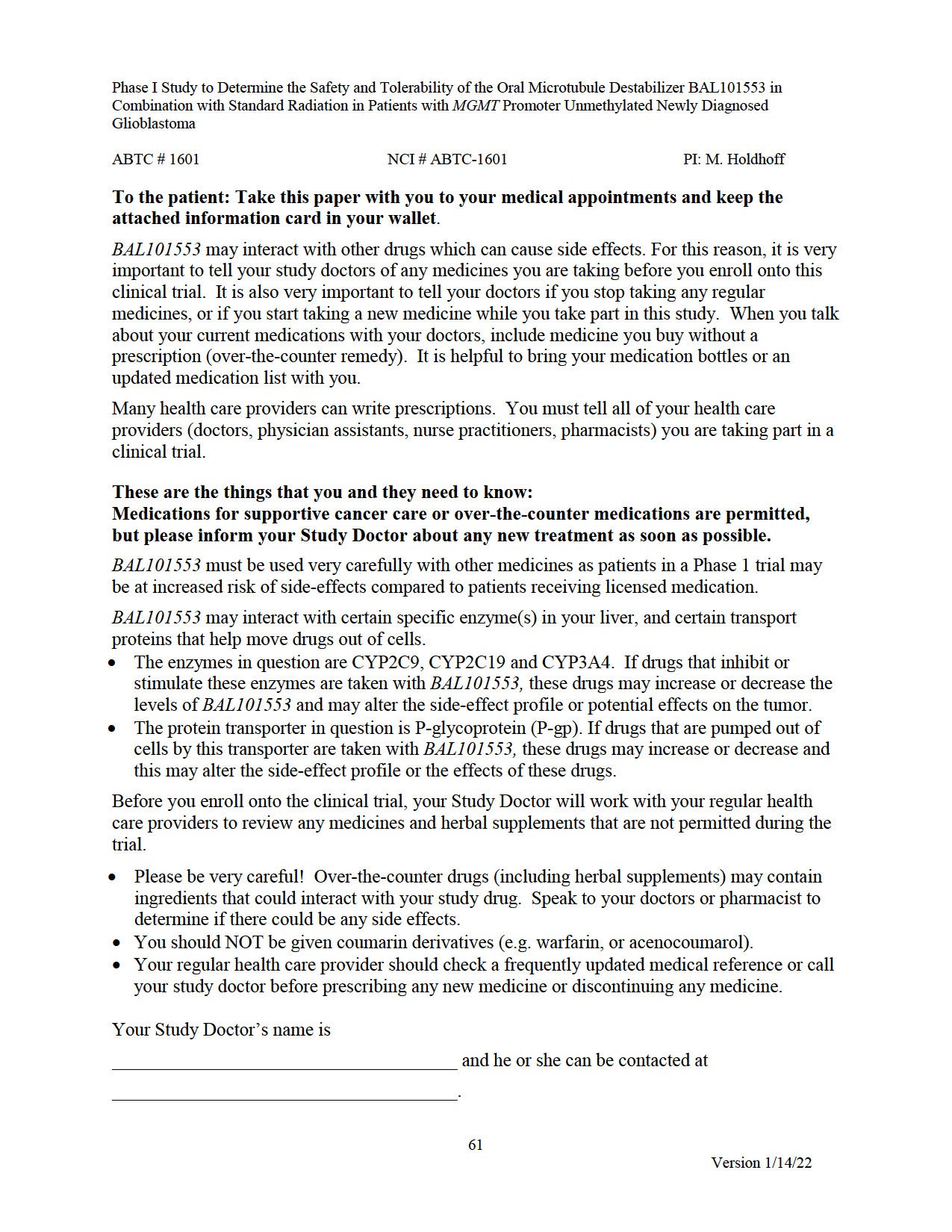


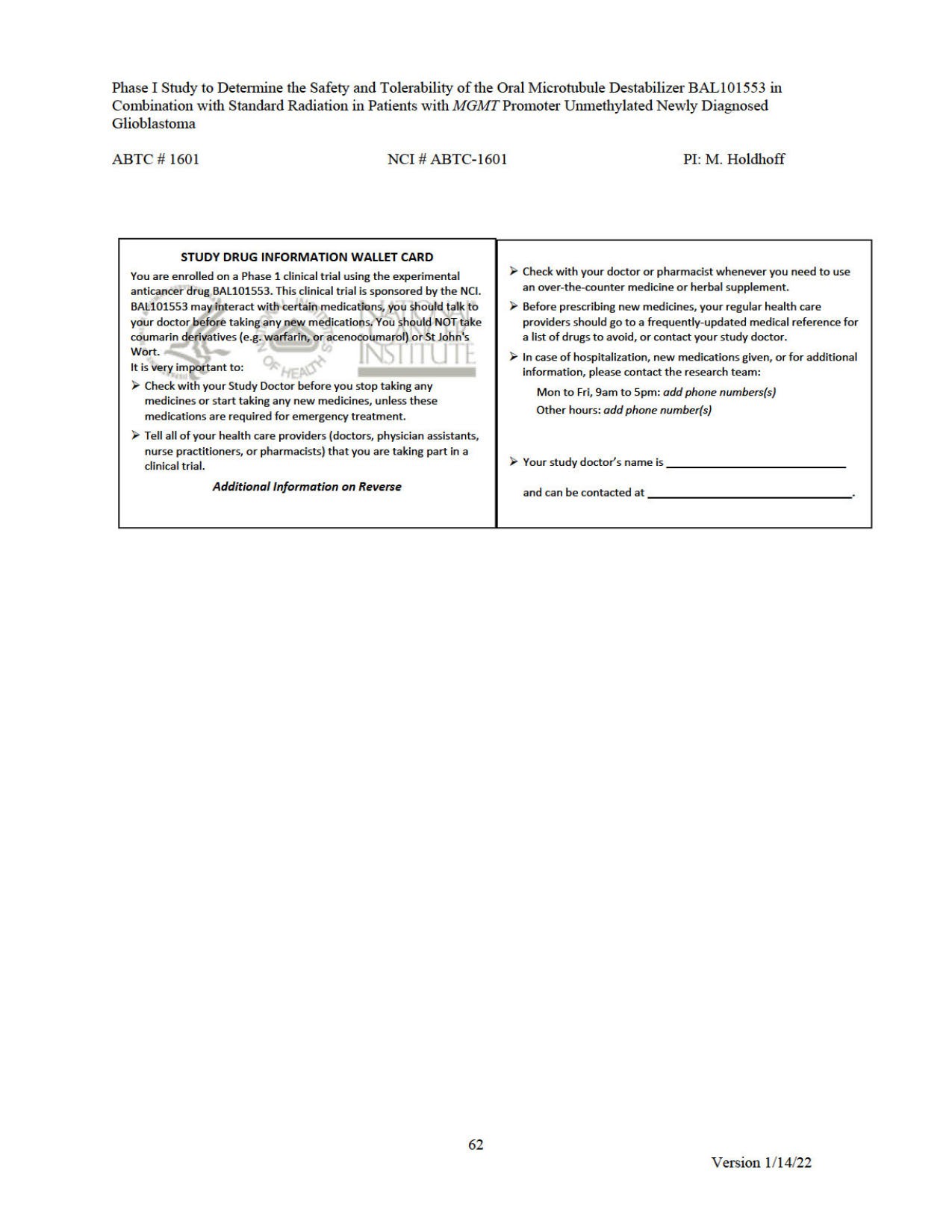


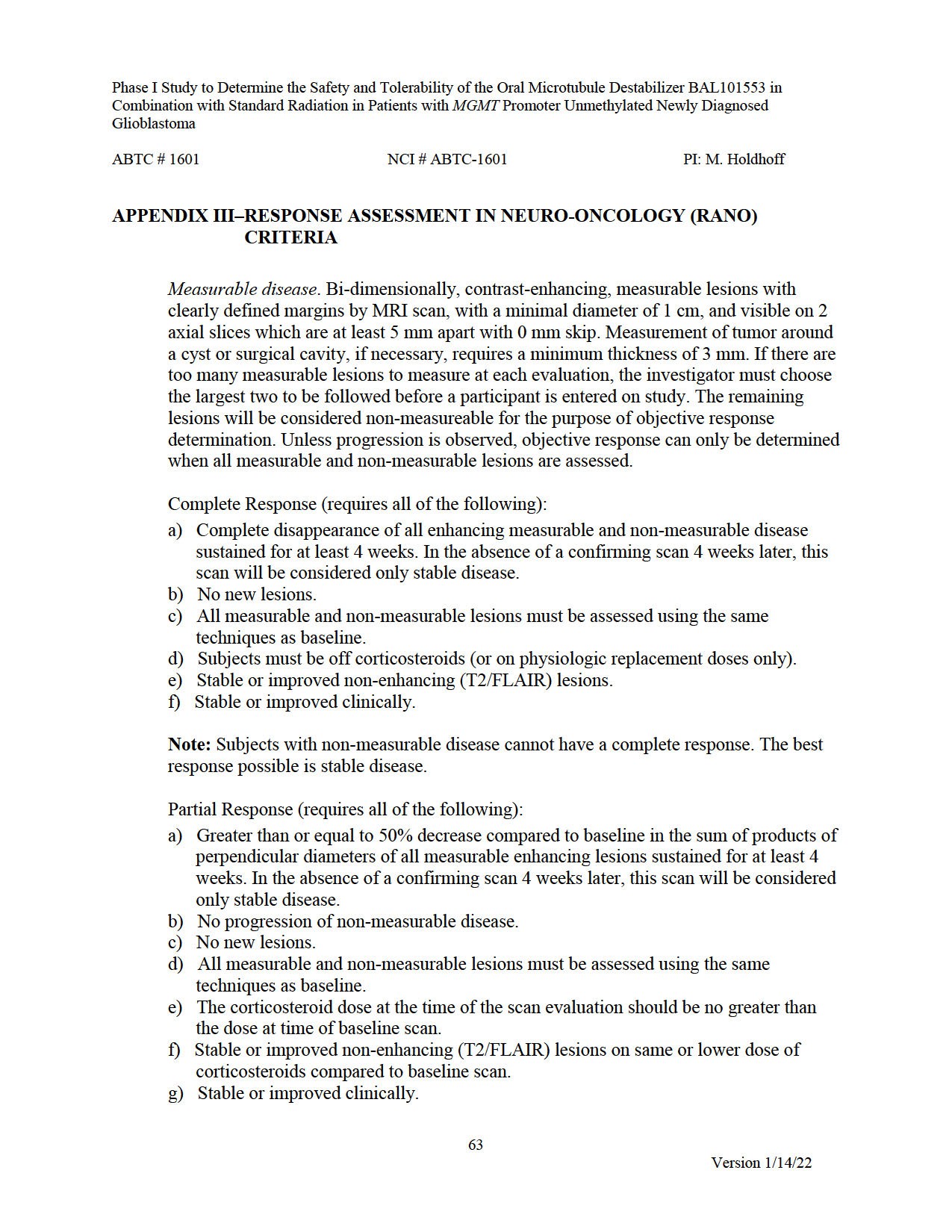


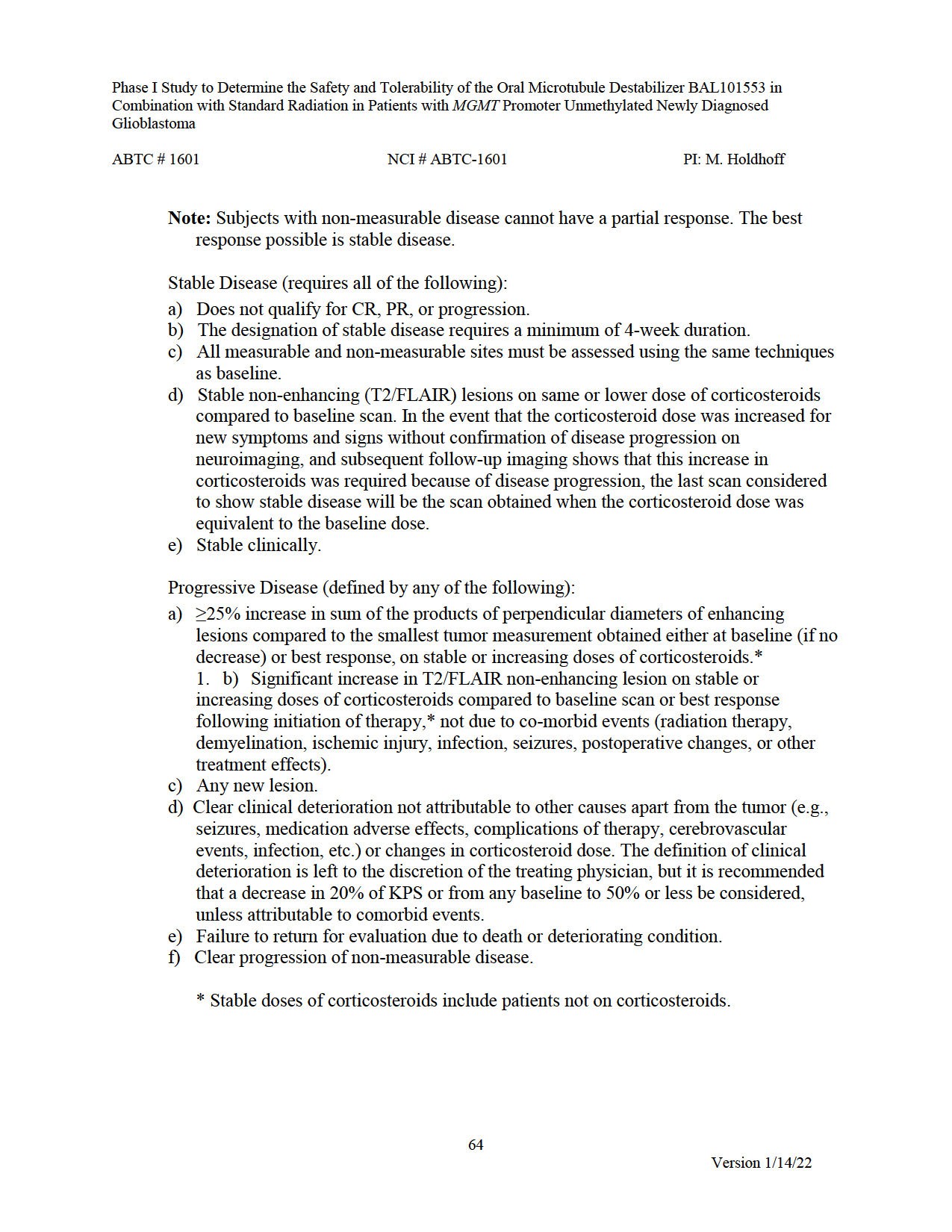


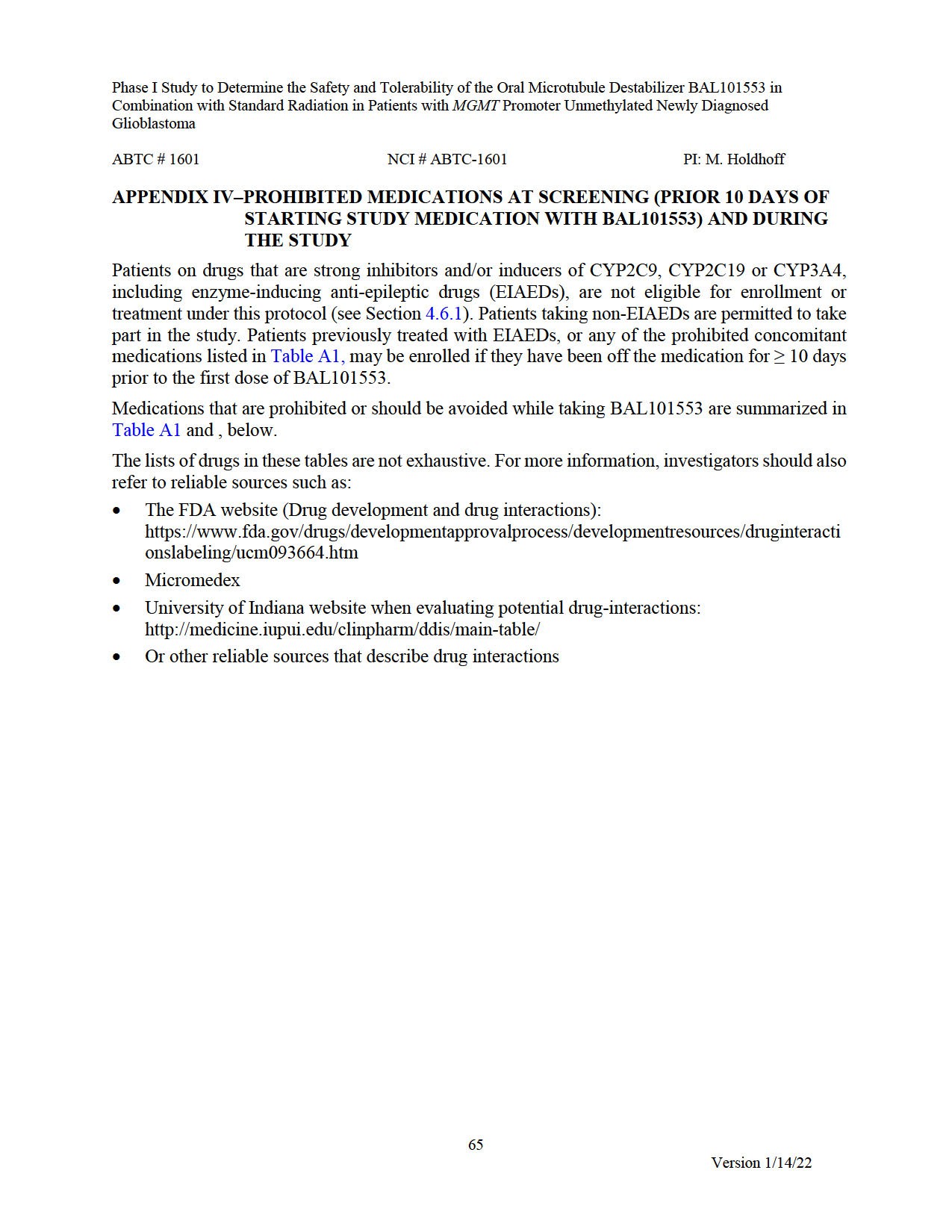


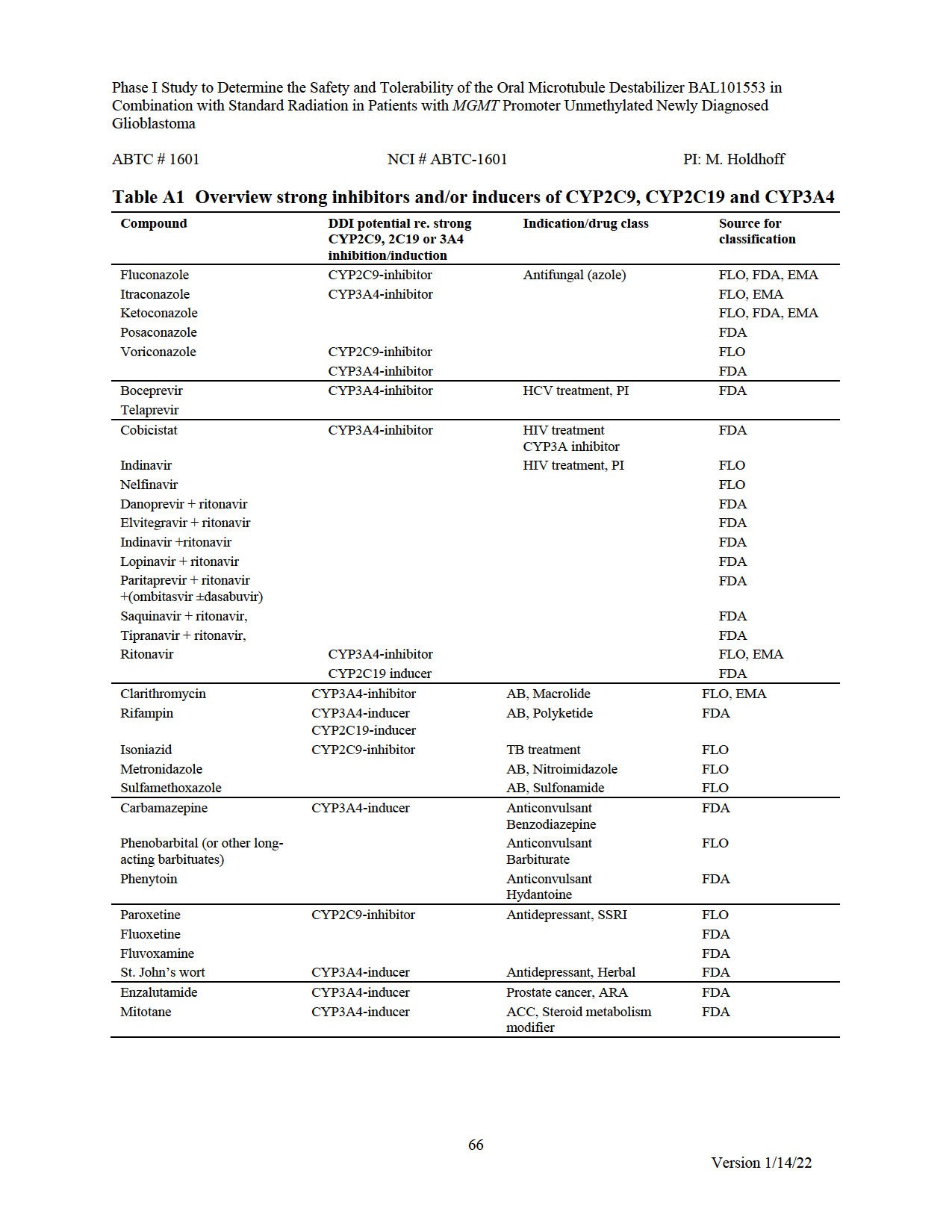


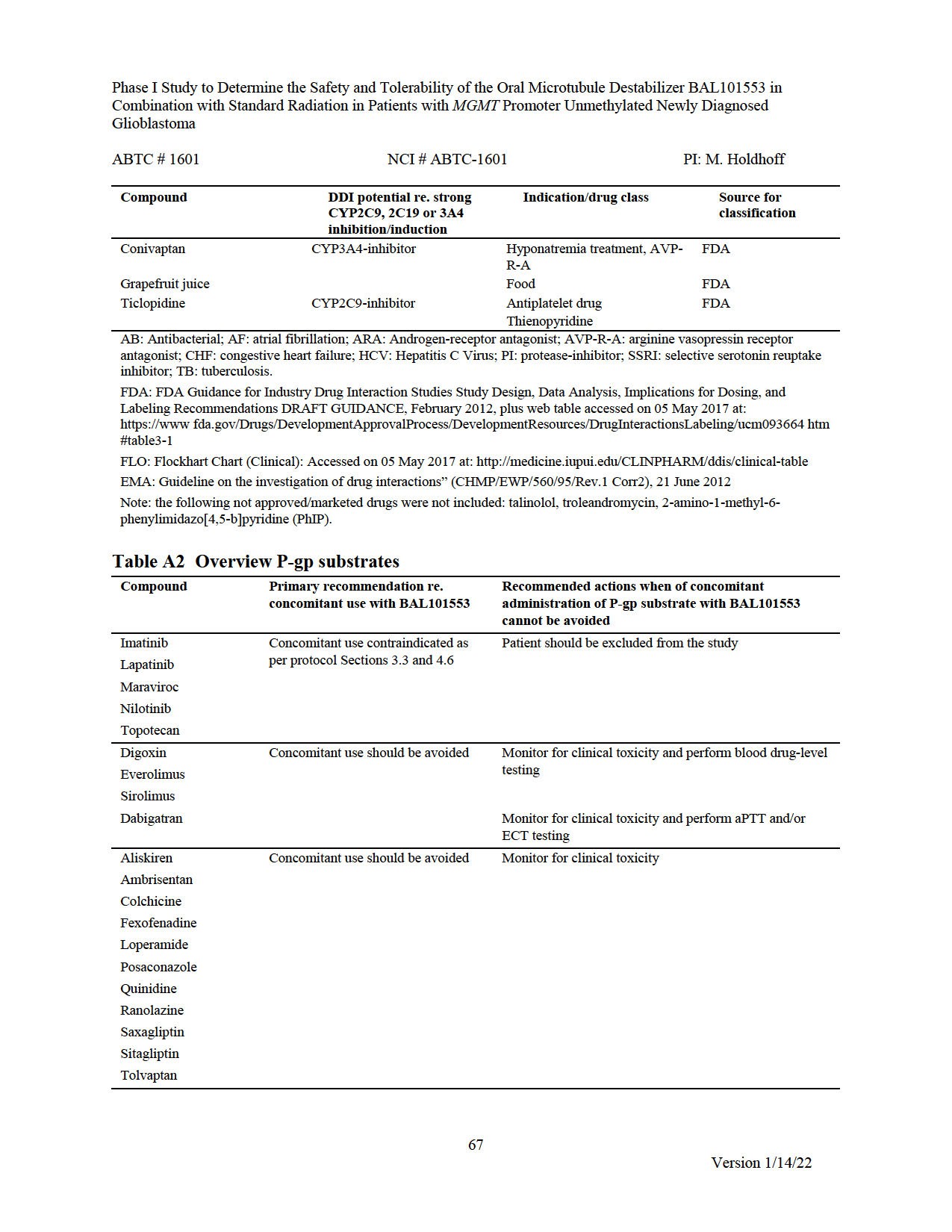

Supplement: vdae150_suppl_Supplementary_Materials [file vdae150_suppl_Supplementary_Materials.zip › Supplementary File_Protocol (redacted).docx]
